# Ten previously undescribed norsesquiterpenoids from the fungus *Marasmiellus candidus* and their anti-inflammatory activities

**DOI:** 10.1080/21501203.2025.2551650

**Published:** 2025-09-12

**Authors:** Lan Yao, Shiyu Li, Jinxiu Zhang, Zhuang Li, Jianhua Lv

**Affiliations:** a College of Life Sciences, Hebei Normal University, Shijiazhuang, China; b Institute of Biology, Hebei Academy of Science, Shijiazhuang, China; c Engineering Research Center of Chinese Ministry of Education for Edible and Medicinal Fungi, Jilin Agricultural University, Changchun, China

**Keywords:** *Marasmiellus candidus*, secondary metabolite, sesquiterpenoids, anti-inflammatory activity

## Abstract

In this study, ten undescribed norsesquiterpenoids, named marasmiellactones R−U (**1**−**4**) and marasmielolic acids A−F (**5**−**10**), were isolated from the cultures of the fungus *Marasmiellus candidus*. Their planar structures were characterised using comprehensive spectroscopic techniques, including 1D/2D NMR and HRESIMS, while the absolute configurations were determined by comparing experimental and calculated ECD spectra. In addition, the anti-inflammatory activities of isolated compounds were evaluated by using an LPS-induce RAW 264.7 cell model. Compounds **1**−**4** demonstrated good anti-inflammatory activity, with IC_50_ values of 18.2, 16.7, 22.1, and 15.5 μmol/L, respectively.

## GRAPHICAL ABSTRACT

**Figure:**
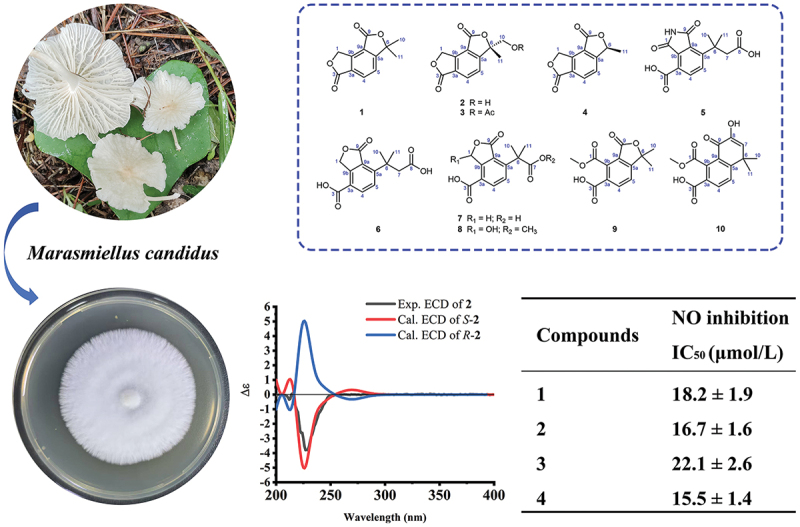

## 1. Introduction

Nitric oxide (NO), a small lipid-soluble radical signaling molecule, serves as a pivotal regulator in the modulation of diverse physiological processes through its diffusible intercellular communication capacity (Cuong et al. 2012). There is evidence that exposure to high levels of NO can result in tissue damage and necrosis, thereby facilitating the development of inflammatory diseases (Li et al. 2018; Lu et al. 2019; Hong et al. 2020). Consequently, the inhibition of NO production represents a viable therapeutic strategy for the treatment of inflammatory diseases. Various natural monomers or extracts that have been isolated from plants or fungi have been demonstrated to effective NO inhibitory activity with relatively low toxicity (Mastrogiovanni et al. 2019; Yao et al. 2019; Wu et al. 2020).

Basidiomycetous fungi are renowned for producing structurally diverse terpenoids with potent biological activities, including anti-inflammatory, anticancer, and antimicrobial properties. The basidiomycetous fungus *Marasmiellus candidus* (Bolt.) Sing. (Agaricales: Omphalotaceae) represents a taxonomically significant species within the cosmopolitan genus *Marasmiellus*, which comprises over 400 described species and subspecies (Datta et al. 2019; Petersen and Hughes 2024). The majority of studies on this genus have concentrated on bio-morphological analysis and species classification (Ichikawa et al. 2001; Kim et al. 2022; Lu et al. 2024). To date, only a few reports have documented secondary metabolites from *Marasmiellus* genus: Yang et al. (2014) found two sesquiterpenoids of the eudesmane type and an androstane derivative in *M. ramealis* that exhibit acetylcholinesterase inhibition; Two novel hirsutane sesquiterpenes were isolated by Isaka et al. (2019) from *Marasmiellus* sp. BCC 22389; Evans et al. (2010) discovered three novel tricyclic cis-caryophyllane sesquiterpenes in *M. troyanus*. These limited findings suggest that the *Marasmiellus* genus may harbour a wealth of terpenoids, particularly sesquiterpenoids, with potential bioactivities.

**Figure 1.**
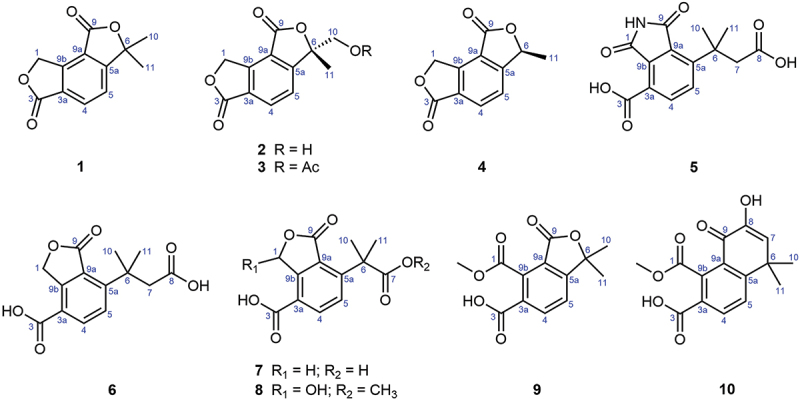
The chemical structures of compounds **1**−**10.**

Our previous investigation on the metabolites and biological activities of the rice fermentation product of *M. candidus* led to the isolation of three new monoterpenoids and seven new sesquiterpenoids, all of which exhibit anti-inflammatory activities (Yao et al. 2025). To further explore the potential of this fungus for discovering novel terpenoids compounds, we implemented the OSMAC (One Strain Many Compounds) methodology through changing the culture conditions of *M. candidus*. As a result, ten new sesquiterpenoids (**1**−**10**) were isolated when cultured in potato dextrose liquid medium (Figure 1). The anti-inflammatory activities demonstrated that compounds **1**−**4** exhibited significant inhibitory effects with IC_50_ values of 18.2, 16.7, 22.1, and 15.5 μmol/L, respectively.

## 2. Materials and methods

### 2.1. General experimental procedures

Infra-red (IR), Ultraviolet (UV), NMR, HR-ESI-MS, and ECD data were obtained as previously reported (Lv et al. 2022; Wang et al. 2025). The fractionation of the extracts and their subsequent fractions were utilised by silica gel, Sephadex LH-20, and ODS. Compounds were purified using an Agilent 1260 HPLC system fitted with a diode array detector and a Silgreen C18 column (250 × 10 mm, 5 µm). HPLC-grade solvents such as acetonitrile (MeCN) and water were obtained from Thermo Fisher Scientific. And all analytical-grade solvents including ethyl acetate (EtOAc), petroleum ether (PE), and methanol (MeOH) for the extraction and purification were obtained from Beijing Chemical Factory.

### 2.2. Fungal material and fermentation

The *M. candidus* strain was obtained from fresh fruiting bodies of *M. candidus*, which were collected from Chengde City, Hebei Province, China, in August 2022. And it was identified by morphological characteristics and ITS sequence analysis. The key morphological features include: the pileus with a diameter of 2–3 cm, flattened shape, wavy margin, and sparse sulcate striations. The obtained ITS sequence (GenBank accession no. PV490809) has been submitted to the GenBank database.

The fungus was grown on potato dextrose agar medium maintained under axenic conditions at 25 ± 2 °C for 10 d in darkness. Standardised inoculation procedures involved transferring two 0.6 × 0.6 cm^2^ mycelial agar plugs to 500 mL Erlenmeyer flasks which contained 200 mL potato dextrose broth. Liquid cultures were placed on an orbital shaker and incubated at 25 °C with constant illumination and 140 r/min agitation for 30 d.

### 2.3. Extraction and isolation

The fermentation product (25 L) was centrifuged at 6,000 × *g* for 15 min at 4 °C using a Beckman J-26XPI centrifuge (Beckman Coulter, USA) to separate the mycelia and supernatant. The supernatant (liquid phase) was concentrated under reduced pressure at 55 °C (vacuum degree: −0.09 MPa) using a RE-52A rotary evaporator (Yarong, Shanghai) to a final volume of 1 L. The concentrated solution was partitioned with EtOAc (1 L × 3) in a 2 L separatory funnel; the combined ethyl acetate phases were dried over anhydrous Na₂SO₄ (100 g) for 12 h, filtered through qualitative filter paper, and concentrated under reduced pressure (45 °C, −0.09 MPa) to afford a crude extract (18.2 g).

The crude extract was processed using silica gel column chromatography (500 g, 200–300 mesh; 70 cm × 6 cm column) eluted with a gradient system of PE and EtOAc (*v*/*v*: 10:1, 8:1, 5:1, 3:1, 1:1, 1:3, 1:5, 1:8, 1:10). And fractions were collected in 200 mL glass flasks. All fractions were monitored by thin-layer chromatography (TLC, GF₂₅₄ silica gel plates; visualisation under UV₂₅₄ and 10% H₂SO₄ ethanol spray followed by heating at 105 °C for 5 min). Twelve distinct fractions (Fr.1–Fr.12) were combined based on TLC.

Fr.7 (3.1 g) was further separated by ODS column chromatography (40 cm × 3 cm column) eluted with a gradient of MeOH-H₂O (*v*/*v*: 10:90, 20:80, 30:70, 40:60, 50:50, 60:40, 70:30, 80:20). Each gradient was eluted with 300 mL of solvent and 10 subfractions (Fr.7.1–Fr.7.10) were collected based on TLC analysis. Fr.7.5 (280 mg) was purified by semi-preparative HPLC (Agilent 1260, USA) using a Silgreen C18 column (250 × 10 mm, 5 µm) with a mobile phase of MeCN-H₂O (25:75, *v*/*v*) at a flow rate of 3.0 mL/min (detection wavelength: 210 nm), yielding compounds **2** (3.7 mg, t_R_ = 8.5 min) and **1** (4.8 mg, t_R_ = 10.5 min). Fr.8 (2.5 g) was loaded onto a Sephadex LH-20 column (120 cm × 2 cm column) and eluted with MeOH; fractions (10 mL each) were collected and combined based on TLC. And compounds **3** (3.5 mg) and **4** (2.1 mg) were obtained. Fr.9 (3.2 g) was fractionated by ODS column chromatography (same as above) using a MeOH-H₂O gradient (*v*/*v*: 10:90, 20:80, 30:70, 40:60, 50:50, 60:40, 70:30, 80:20, 90:10), yielding 6 subfractions (Fr.9.1–Fr.9.6). Fr.9.3 (320 mg) was purified by semi-preparative HPLC (Agilent 1260) with a MeCN-H₂O (10:90, *v*/*v*) mobile phase (3.0 mL/min, 210 nm), giving compound **5** (3.2 mg, t_R_ = 25.5 min), compound **7** (2.2 mg, t_R_ = 28.3 min), and compound **8** (4.4 mg, t_R_ = 32.5 min). Fr.9.6 (290 mg) was purified by semi-preparative HPLC using MeCN-H₂O (5:95, *v*/*v*) at 3.0 mL/min (210 nm), affording compound **9** (2.6 mg, t_R_ = 9.7 min), compound **10** (2.5 mg, t_R_ = 10.7 min), and compound **6** (3.7 mg, t_R_ = 15.3 min).

Marasmiellactone R (**1**): white amorphous powder; IR*v*_max_ 3,439, 2,983, 1,775, 1,399, 1,295, 1,082 cm^−1^; UV (MeOH) *λ*_max_ 212, 296 nm; ^1^H and ^13^C NMR data (Table 1); HR-ESI-MS (*m/z* 219.0651 [M+H]^+^, calcd. 219.0652).

**Table 1.** The ^1^H and ^13^C NMR spectral data for compounds **1**‒**4**.

No.	1 ^a)^		2 ^b)^		3 ^a)^		4 ^b)^	
	^1^H NMR	^13^C NMR	^1^H NMR	^13^C NMR	^1^H NMR	^13^C NMR	^1^H NMR	^13^C NMR
1	5.65 (2 H, s)	70.4	5.67 (1 H, d, 16.7) 5.72 (1 H, d, 16.7)	68.9	5.66 (1 H, d, 16.9) 5.71 (1 H, d, 16.9)	70.4	5.67 (1 H, d, 16.8) 5.71 (1 H, d, 16.8)	68.9
3	―	171.5	―	169.3	―	171.3	―	169.3
3a	―	128.5	―	126.6	―	129.2	―	126.5
4	8.18 (1 H, d, 7.9)	132.1	8.22 (1 H, d, 7.9)	130.5	8.21 (1 H, d, 7.9)	132.1	8.23 (1 H, d, 7.9)	130.7
5	7.83 (1 H, d, 7.9)	123.8	7.93 (1 H, d, 7.9)	123.4	7.88 (1 H, d, 7.9)	124.5	7.92 (1 H, d, 7.9)	123.7
5a	―	162.5	―	158.1	―	158.2	―	157.5
6	―	88.9	―	89.9	―	88.5	5.92 (1 H, q, 6.7)	79.3
9	―	169.2	―	167.5	―	168.8	―	167.9
9a	―	121.9	―	121.6	―	123.0	―	120.5
9b	―	147.3	―	145.2	―	147.1	―	145.6
10	1.73 (3 H, s)	27.1	3.80 (1 H, dd, 11.8, 3.4) 3.84 (1 H, dd, 11.8, 4.2)	65.4	4.51 (1 H, d, 12.0) 4.57 (1 H, d, 12.0)	67.7	―	―
11	1.73 (3 H, s)	27.1	1.62 (3 H, s)	21.2	1.76 (3 H, s)	22.2	1.63 (3 H, d, 6.7)	19.7
1’	―	―	―	―	―	171.5	―	―
2’	―	―	―	―	1.88 (3 H, s)	20.2	―	―

^a), b)^ measured in CD_3_OD and DMSO-*d*_6_, respectively. 10-OH: 5.31 (1 H, dd, 4.2, 3.4) for **2**.

Marasmiellactone S (**2**): white amorphous powder; IR*v*_max_ 3,438, 2,979, 1,639, 1,384, 1,233, 1,064, 1,025 cm^−1^; UV (MeOH) *λ*_max_ 210, 295 nm; CD (MeOH) *λ*_max_ (∆*ε*): 228 (−3.92) nm; ${\left[\alpha \right]}_{D}^{21}$ + 19.5 (c 0.1, MeOH); ^1^H and ^13^C NMR data (Table 1); HR-ESI-MS (*m/z* 235.0603 [M+H]^+^, calcd. 235.0601).

Marasmiellactone T (**3**): white amorphous powder; IR*v*_max_ 3,439, 2,929, 1,436, 1,399, 1,233, 1,076 cm^−1^; UV (MeOH) *λ*_max_ 210, 289 nm; CD (MeOH) *λ*_max_ (∆*ε*): 226 (−3.08) nm; ${\left[\alpha \right]}_{D}^{21}$+ 9.8 (c 0.1, MeOH); ^1^H and ^13^C NMR data (Table 1); HR-ESI-MS (*m/z* 277.0709 [M+H]^+^, calcd. 277.0707).

Marasmiellactone U (**4**): white amorphous powder; IR*v*_max_ 3,439, 2,968, 1,754, 1,384, 1,239, 1,042 cm^−1^; UV (MeOH) *λ*_max_ 208, 290 nm; CD (MeOH) *λ*_max_ (∆*ε*): 223 (−2.04) nm; ${\left[\alpha \right]}_{D}^{21}$+ 27.5 (c 0.1, MeOH); ^1^H and ^13^C NMR data (Table 1); HR-ESI-MS (*m/z* 205.0506 [M+H]^+^, calcd. 205.0495).

Marasmielolic acid A (**5**): white amorphous powder; IR*v*_max_ 3,424, 2,972, 1,720, 1,433, 1,202, 1,046, 1,025 cm^−1^; UV (MeOH) *λ*_max_ 218, 296 nm; ^1^H and ^13^C NMR data (Table 2); HR-ESI-MS (*m/z* 290.0670 [M‒H]^‒^, calcd. 290.0670).

**Table 2.** The ^1^H and ^13^C NMR spectral data for compounds **5** and **6**.

No.	5 ^a)^		5 ^b)^		6 ^a)^	
	^1^H NMR	^13^C NMR	^1^H NMR	^13^C NMR	^1^H NMR	^13^C NMR
1	―	171.4	―	169.2	5.59 (2 H, s)	71.7
3	―	168.0	―	167.3	―	167.7
3a	―	129.8	―	129.6	―	125.2
4	8.09 (1 H, d, 8.3)	136.2	7.78 (1 H, s)	132.2	8.24 (1 H, d, 8.2)	136.5
5	7.93 (1 H, d, 8.3)	135.1	7.78 (1 H, s)	133.4	7.71 (1 H, d, 8.2)	129.3
5a	―	153.5	―	149.9	―	156.2
6	―	38.7	―	37.1	―	39.0
7	3.25 (2 H, s)	45.7	3.10 (2 H, s)	44.4	3.29 (2 H, s)	45.6
8	―	175.4	―	172.8	―	175.6
9	―	170.1	―	167.6	―	172.4
9a	―	131.2	―	129.3	―	125.2
9b	―	133.2	―	130.3	―	152.8
10	1.61 (3 H, s)	29.0	1.51 (3 H, s)	28.3	1.62 (3 H, s)	29.0
11	1.61 (3 H, s)	29.0	1.51 (3 H, s)	28.3	1.62 (3 H, s)	29.0

^a), b)^ measured in CD_3_OD and DMSO-*d*_6_, respectively. NH: 11.59 (1 H, s); 2 × COOH: 11.86, 13.55 (each 1 H, vbr s) for **5** in DMSO-*d*_6_.

Marasmielolic acid B (**6**): white amorphous powder; IR*v*_max_ 3,439, 2,969, 1,715, 1,398, 1,202, 1,045, 1,002 cm^−1^; UV (MeOH) *λ*_max_ 208, 293 nm; ^1^H and ^13^C NMR (Table 2); HR-ESI-MS (*m/z* 277.0717 [M‒H]^‒^, calcd. 277.0718).

Marasmielolic acid C (**7**): white amorphous powder; IR*v*_max_ 3,431, 3,172, 2,924, 1,400, 1,290, 1,008 cm^−1^; UV (MeOH) *λ*_max_ 210, 296 nm; ^1^H and ^13^C NMR data (Table 3); HR-ESI-MS (*m/z* 287.0523 [M+Na]^+^, calcd. 287.0526).

**Table 3.** The ^1^H and ^13^C NMR spectral data for compounds **7** and **8**.

No.	7 ^a)^		8 ^b)^	
	^1^H NMR	^13^C NMR	^1^H NMR	^13^C NMR
1	5.58 (2 H, s)	70.4	6.95 (1 H, s)	98.8
3	―	165.9	―	167.2
3a	―	123.8	―	126.1
4	8.19 (1 H, d, 8.1)	135.3	8.27 (1 H, d, 8.1)	137.3
5	7.68 (1 H, d, 8.1)	126.6	7.78 (1 H, d, 8.1)	129.1
5a	―	150.3	―	150.5
6	―	45.4	―	47.2
7	―	176.8	―	178.3
9	―	169.6	―	170.1
9a	―	124.5	―	127.7
9b	―	149.9	―	150.6
10	1.57 (3 H, s)	26.4	1.65 (3 H, s)	26.9
11	1.57 (3 H, s)	26.4	1.67 (3 H, s)	26.9
OCH_3_	―	―	3.63 (3 H, s)	52.7

^a), b)^ measured in DMSO-*d*_6_ and CD_3_OD, respectively. 2 × COOH: 12.94 (2 H, vbr s) for **7**.

(±)-Marasmielolic acid D (**8**): white amorphous powder; IR*v*_max_ 3,434, 1,763, 1,290, 1,026 cm^−1^; UV (MeOH) *λ*_max_ 212, 297 nm; ^1^H and ^13^C NMR data (Table 3); HR-ESI-MS (*m/z* 293.0666 [M‒H]^‒^, calcd. 293.0667).

Marasmielolic acid E (**9**): white amorphous powder; IR*v*_max_ 3,435, 3,138, 1,735, 1,101, 1,009 cm^−1^; UV (MeOH) *λ*_max_ 210, 288 nm; ^1^H and ^13^C NMR data (Table 4); HR-ESI-MS (*m/z* 287.0524 [M+Na]^+^, calcd. 287.0526).

**Table 4.** The ^1^H and ^13^C NMR spectral data for compounds **9** and **10** in DMSO-*d*_6._

No.	9		10	
	^1^H NMR	^13^C NMR	^1^H NMR	^13^C NMR
1	―	165.8	―	168.6
3	―	165.2	―	165.8
3a	―	128.8	―	127.6
4	8.33 (1 H, d, 8.0)	136.1	8.17 (1 H, d, 8.4)	133.4
5	7.96 (1 H, d, 8.0)	123.1	8.00 (1 H, d, 8.4)	128.5
5a	―	158.6	―	155.3
6	―	85.6	―	37.6
7	―	―	6.23 (1 H, s)	128.1
8	―	―	―	145.6
9	―	166.3	―	179.3
9a	―	121.7	―	126.7
9b	―	133.1	―	135.1
10	1.66 (3 H, s)	26.5	1.48 (3 H, s)	30.0
11	1.66 (3 H, s)	26.5	1.48 (3 H, s)	30.0
OCH_3_	3.85 (3 H, s)	52.6	3.82 (3 H, s)	52.1
CO*OH*	13.77 (1 H, vbr s)	―	13.50 (1 H, vbr s)	―
*OH*	―	―	8.96 (1 H, vbr s)	―

Marasmielolic acid F (**10**): white amorphous powder; IR*v*_max_ 3,420, 2,971, 1,728, 1,201, 1,046 cm^−1^; UV (MeOH) *λ*_max_ 212, 288 nm; ^1^H and ^13^C NMR data (Table 4); HR-ESI-MS (*m/z* 313.0680 [M+Na]^+^, calcd. 313.0683).

### 2.4. Anti-inflammatory activity assay

#### 2.4.1. Cell viability assay

RAW264.7 macrophages were seeded in 96-well plates (5 × 10^4^ cells/well) and cultured at 37 °C with 5% CO₂ for 24 h. After adhesion, test samples (20, 40, 80 μmol/L) or medium (blank) were added and incubated for 24 h. Subsequently, 10 μL of CCK-8 solution was added, and after 2 h of incubation, the absorbance was measured at 450 nm.

Cell viability (%) = (OD_sample_/OD_blank_) × 100%

#### 2.4.2. Determination of NO production

RAW 264.7 cells were cultured as above, then divided into blank control, LPS-induced (10 μg/mL, model), and sample-treated groups. After 24 h, supernatants were centrifuged (12,000 r/min, 5 min) and collected. NO production was assessed using the standard Griess reagent method (Fan et al. 2023).

Inhibition rate (%) = [(NO_model_ – NO_sample_)/(NO_model_ – NO_blank_)] × 100%.

### 2.5. Quantum chemical ECD calculations

The method used for ECD calculation has been previously reported (Ukwatta et al. 2019). Monte Carlo conformational searches were done with Spartan’14 (MMFF force field). And conformers with a Boltzmann population > 5% were selected, initially optimised at B3LYP/6-31 G in gas. ECD calculations in MeOH used TD-DFT at B3LYP/6–31 + G (d,p) for 30 excited states. Spectra were generated with SpecDis 1.6 and GraphPad Prism 5 using Gaussian bands (sigma = 0.3 eV).

## 3. Results and discussion

**Figure 2.**
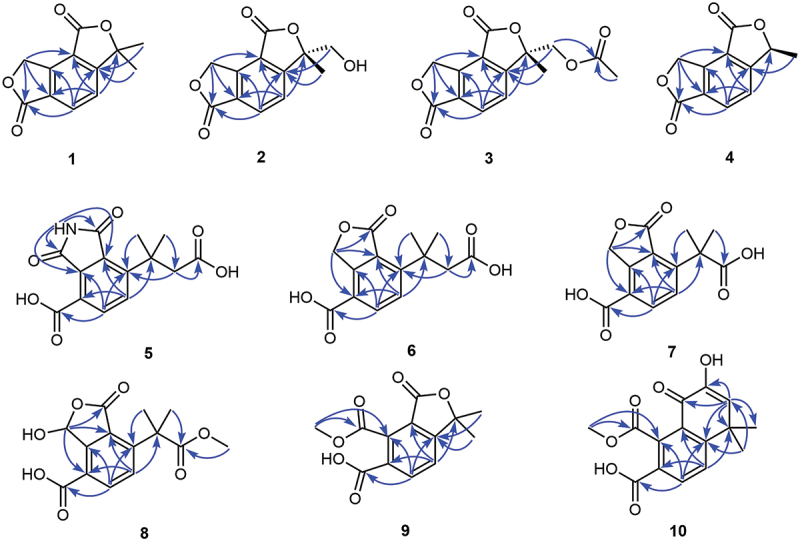
The significant HMBC correlations of compounds **1**−**10**.

Compound **1** was found to have the molecular formula C_12_H_10_O_4_, as evidenced by positive HR-ESI-MS data (*m/z* 219.0651 [M+H]^+^, calcd. 219.0652), which implies an unsaturation degree of eight. In the ^1^H NMR spectrum of compound **1** (recorded in CD₃OD, Table 1), two aromatic doublets with mutual coupling were observed: one at *δ*_H_ 8.18 (1 H, d, *J* = 7.9 Hz, H-4) and the other at 7.83 (1 H, d, *J* = 7.9 Hz, H-5). Additionally, a low-field singlet from the oxygenated methylene group was observed at *δ*_H_ 5.65 (2 H, s, H-1), along with two methyl singlets appearing at *δ*_H_ 1.73 (6 H, s). The ^13^C NMR spectrum (Table 1) revealed 12 carbon signals. Among them, two lactone carbonyl carbons were identified at *δ*_C_ 171.5 (C-3) and 169.2 (C-9), while six aromatic carbons arose from a benzene ring structure. An oxygen-containing carbon was detected at *δ*_C_ 88.9 (C-6), and an oxygenated methylene carbon appeared at *δ*_C_ 70.4 (C-1). Additionally, two methyl carbons were observed at *δ*_C_ 27.1. The benzene ring and two carbonyls led to six degrees of unsaturation, signifying the presence of two aliphatic rings in the structure. The HMBC correlations (Figure 2) of H-1 [*δ*_H_ 5.65 (2 H, s)] and H-4 [*δ*_H_ 8.18 (1 H, d, *J* = 7.9 Hz)] with the lactone carbonyl carbon at *δ*_C_ 171.5 (C-3) revealed the existence of an isobenzofuran-3(1 H)-one moiety. While the HMBC correlation observed from H-5 [*δ*_H_ 7.83 (1 H, d, *J* = 7.9 Hz)] to the low-field oxygenated quaternary carbon at *δ*_C_ 88.9 (C-6), along with the correlations from the two methyl proton signals to C-5a [*δ*_C_ 162.5], when considered together with the degrees of unsaturation, allowed to draw a conclusion that the other *γ*-lactone ring was fused to the benzene ring. The structure was completely confirmed using 2D NMR analysis and named marasmiellactone R, which belongs to an unusual class of aromatic trinorsesquiterpenoids.

**Figure 3.**
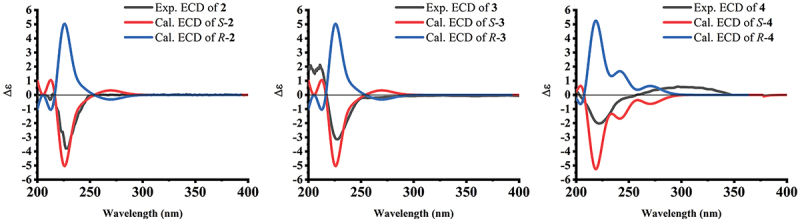
Comparison of experimental and calculated ECD spectra of **2**−**4**.

Compound **2** had a molecular formula of C_12_H_10_O_5_ (positive HR-ESI-MS: *m/z* 235.0603 [M+H]^+^, calcd. 235.0601), containing one more oxygen atom than compound **1**. The ^1^H and ^13^C NMR spectroscopic data (Table 1) in DMSO-*d*_6_ of **2** showed a high degree of similarity to those of compound **1**. The most notable distinction was the absence of one methyl signal, which was substituted by a hydroxymethyl resonance signal [*δ*_H_ 3.80 (1 H, dd, *J* = 11.8, 3.4 Hz, H-10a) and 3.84 (1 H, dd, *J* = 11.8, 4.2 Hz, H-10b); *δ*_C_ 65.4 (C-10)], indicating that **2** is an oxygen-containing derivative of **1**. The HMBC correlations between the remaining methyl singlet [*δ*_H_ 1.62 (3 H, s, H-11)] and both C-5a (*δ*_C_ 158.1) and C-10 (*δ*_C_ 65.4) confirmed hydroxylation of a methyl group at C-6, forming a chiral center. The proposed planar structure was completely confirmed using 2D NMR analysis. Moreover, through comparison of experimental and calculated ECD spectra (Figure 3), the absolute configuration at the C-6 position was determined to be the *S*-form. On this basis, the structure of compound 2 was identified and given the name marasmiellactone S.

Compound **3** was found to have the molecular formula C_14_H_12_O_6_, as evidenced by positive HR-ESI-MS data (*m/z* 277.0709 [M+H]^+^, calcd. 277.0707). The ^1^H and ^13^C NMR features (Table 1) in CD_3_OD of **3** showed a high degree of similarity to those of compound **2**, with the addition of an *O*-acetyl group [*δ*_H_ 1.88 (3 H, s); *δ*_C_ 171.5 (s) and 20.2 (q)]. A significant downfield shift of H_2_-10 [*δ*_H_ 4.51, 4.57 (each 1 H, d, *J* = 12.0 Hz)] suggests that the primary hydroxy in **2** should be acetylated in **3**. The inference was validated through HMBC correlations from H_2_-10 to C-5a (*δ*_C_ 158.2), C-11 (*δ*_C_ 22.2), and the *O*-acetyl carbonyl carbon [*δ*_C_ 171.5 (C-1’)]. And the absolute configuration of C-6 was found to align with that of **2** by the high consistency of the experimental CD curves (Figure 3). Thus, compound **3** was determined to be 10-*O*-acetylmarasmiellactone S, shown in Figure 1 and given the name marasmiellactone T.

Compound **4** was found to have the molecular formula C_11_H_8_O_4_, as evidenced by positive HR-ESI-MS data (*m/z* 205.0506 [M+H]^+^, calcd. 205.0495). The ^1^H and ^13^C NMR data (Table 1) in DMSO-*d*_6_ of compound **4** showed a high degree of similarity to those of compound **1**, suggesting that it also possessed an isobenzofuran-3(1*H*)-one moiety. The primary distinction arose solely from the other lactone ring, in which the ^1^H NMR signals were as follows: a low-field oxygenated methine quartet at *δ*_H_ 5.92 (1 H, q, *J* = 6.7 Hz, H-6) and a methyl doublet at *δ*_H_ 1.63 (3 H, d, *J* = 6.7 Hz, H-11), indicating that the C-10 methyl group was absent. The HMBC correlations observed from methyl doublet to C-5a (*δ*_C_ 157.5), and from H-5 [*δ*_H_ 7.92 (1 H, d, *J* = 7.9 Hz)] to C-6 (*δ*_C_ 79.3), further confirmed the above inference. Moreover, through comparison of experimental and calculated ECD spectra (Figure 3), the absolute configuration at the C-6 position was determined to be the *S*-form. The structure of **4** was determined as 10-normarasmiellactone R and given the name marasmiellactone U.

Compound **5** was found to have the molecular formula C₁₄H₁₃NO₆, as evidenced by negative HR-ESI-MS data (*m/z* 290.0670 [M−H]^−^, calcd. 290.0670), which implies an unsaturation degree of nine. Its ^1^H NMR spectrum (Table 2) in CD₃OD showed aromatic doublets *δ*_H_ 8.09 (1 H, d, *J* = 8.3 Hz, H-4) and 7.93 (1 H, d, *J* = 8.3 Hz, H-5), an aliphatic methylene singlet at *δ*_H_ 3.25 (2 H, s, H-7), and two methyl singlets at *δ*_H_ 1.61 (6 H, s). The ^1^H and ^13^C NMR spectrum (Table 2) revealed 14 carbon signals. These included four carbonyl carbons at *δ*_C_ 175.4 (C-8), 171.4 (C-1), 170.1 (C-9), and 168.0 (C-3), six aromatic carbons, and four aliphatic carbons. The benzene ring and four carbonyls led to eight degrees of unsaturation, signifying the presence of one aliphatic ring in the structure. The HMBC correlations (Figure 2) from H-4 to the carbonyl carbon at *δ*_C_ 168.0 (C-3), from H-5 to the aliphatic quaternary carbon at *δ*_C_ 38.7 (C-6), from the two methyl singlets to C-5a (*δ*_C_ 157.5) and C-7 (*δ*_C_ 45.7), and from H-7 [*δ*_H_ 3.25 (2 H, s)] to C-5a and C-8 (*δ*_C_ 175.4), revealed the presence of a 4-(1-carboxy-2-methylpropan-2-yl)benzoic acid moiety. Taking into account the remaining two unresolved carbonyl signals, as well as the existence of a nitrogen atom and an additional ring, allowed to infer the fusion of a pyrrolidine-2,5-dione ring to the benzene ring. It was further determined by the observable HMBC correlations of the sharp NH singlet at *δ*_H_ 11.59 (1 H, s) with C-1, C-9, C-9a, and C-9b using DMSO-*d*_6_ as a solvent. The proposed structure was fully confirmed using 2D NMR analysis in two different deuterated solvents, CD_3_OD and DMSO-*d*_6_. Finally, the structure of **5** was determined and given the name marasmielolic acid A. It was a highly modified derivative derived from marasmiellactones.

Compound **6** was determined to have a molecular formula C_14_H_14_O_6_, as evidenced by negative HR-ESI-MS data (*m/z* 277.0717 [M‒H]^−^, calculated as 277.0718), which implies an unsaturation degree of eight. In the ^1^H NMR spectrum of compound **6** (recorded in CD₃OD, Table 2), two aromatic doublets with mutual coupling were observed at *δ*_H_ 8.24 (1 H, d, *J* = 8.2 Hz, H-4) and 7.71 (1 H, d, *J* = 8.2 Hz, H-5). Additionally, there was a low-field singlet for an oxygenated methylene group *δ*_H_ 5.59 (2 H, s, H-1), an aliphatic methylene singlet at *δ*_H_ 3.29 (2 H, s, H-7), and two methyl singlets at *δ*_H_ 1.62 (6 H, s). The ^13^C NMR spectrum (Table 2) revealed 14 carbon signals. These comprised three carbonyl carbons at *δ*_C_ 175.6 (C-8), 172.4 (C-9), and 167.7 (C-3), six aromatic carbons originating from a benzene ring, an oxygen-containing methylene carbon at *δ*_C_ 71.7 (C-1), and four aliphatic carbons. The above NMR features closely resembled those of **5**. And the most distinct variation was the lack of one carbonyl signal, which was substituted by an oxygenated methylene signal, suggesting that the pyrrolidine-2,5-dione moiety in **5** should be changed to a *γ*-lactone ring in **6**. The HMBC correlations (Figure 2) from the low-field oxygenated methylene singlet at *δ*_H_ 5.59 (2 H, s) to C-3a (*δ*_C_ 125.2), C-9 (*δ*_C_ 172.4), and C-9a (*δ*_C_ 125.2), and from H-5 to C-3a, C-6, and C-9a, confirmed that the oxygenated methylene was positioned at C-1 and formed a *γ*-lactone with the C-9 carbonyl group. The proposed structure was further confirmed through 2D NMR analysis. Thus, the structure of **6** was confirmed and given the name marasmielolic acid B.

Compound **7** was determined to have the molecular formula C_13_H_12_O_6_, as evidenced by HR-ESI-MS data (*m/z* 287.0523 [M+Na]^+^, calcd. 287.0526), with the same degrees of unsaturation as that of **6**. The ^1^H and ^13^C NMR spectral features (Table 3) were highly similar to those of 6, with the notable distinction being the absence of the aliphatic methylene signal present in **6**. HMBC correlations were detected from two methyl singlets at *δ*_H_ 1.57 (6 H, s) to C-5a (*δ*_C_ 150.3) and the carboxyl carbon at *δ*_C_ 176.8 (C-7), which revealed that a 2-carboxypropan-2-yl moiety was substituted at the C-5a position. While the other parts of the structure were shown to be consistent with those of **6** by 2D NMR analysis. Thus, the structure of **7** was determined as normarasmielolic acid B and given the name marasmielolic acid C.

Compound **8** was determined to have the molecular formula C_14_H_14_O_7_, as evidenced by negative HR-ESI-MS data (*m/z* 293.0666 [M‒H]^‒^, calcd. 293.0667), with the same degrees of unsaturation as that of **7**. The ^1^H and ^13^C NMR data (Table 3) were closely resembled to those of **7**, and the most distinct variation was that the oxygenated methylene signal disappeared and was replaced by a newly emerging hemiacetal signal [*δ*_H_ 6.95 (1 H, s, H-1); *δ*_C_ 98.8 (C-1)], in addition, a methyl ester signal [*δ*_H_ 3.63 (3 H, s); *δ*_C_ 52.7] was also detected, which indicated that **8** was a further hemiacetalized derivative of **7**. HMBC correlations were detected from the hemiacetal proton signal to C-3a (*δ*_C_ 126.1) and C-9 (*δ*_C_ 170.1), which positioned the hemiacetal group at C-1. Moreover, the carboxylic acid group connected to C-6 was shown to be methylated according to the HMBC correlations from the two methyl singlets and the methoxy signal to C-7 (*δ*_C_ 178.3). The obtained structure was completely confirmed using 2D NMR analysis. Owing to the nature of hemiacetal tautomerism, this compound was isolated as racemates. As a result, the structure of **8** was confirmed and given the name (±)-marasmielolic acid D.

Compound **9** was found to have the molecular formula of C_13_H_12_O_6_, as evidenced by the positive HR-ESI-MS data (*m/z* 287.0524 [M+Na]^+^, calcd. 287.0526), which implies an unsaturation degree of eight. In the ^1^H NMR spectrum of compound **9** (Table 4), two aromatic doublets with mutual coupling were observed at *δ*_H_ 8.33 (1 H, d, *J* = 8.0 Hz, H-4) and 7.96 (1 H, d, *J* = 8.0 Hz, H-5). Additionally, there was a very broad singlet at *δ*_H_ 13.77 (1 H, vbr s, COOH), a methoxy signal at *δ*_H_ 3.85 (3 H, s), and two methyl singlets at *δ*_H_ 1.66 (6 H, s). The ^13^C NMR spectrum (Table 4) revealed a total of 13 carbon resonance signals. These included three aromatic carboxyl or ester carbonyl carbons at *δ*_C_ 166.3 (C-9), 165.8 (C-1), and 165.2 (C-3), six aromatic carbons derived from a benzene ring, an oxygen-containing quaternary carbon at *δ*_C_ 85.6 (C-6), a methyl ester carbon at *δ*_C_ 52.6, and two methyl carbons at *δ*_C_ 26.5. With three carbonyl groups and one benzene ring contributing seven degrees of unsaturation, it can be deduced that the structure contains one aliphatic ring. Comparison of the NMR data with those of **1**, especially the significant low-field shift of C-6 (*δ*_C_ 85.6), revealed the fusion of a *γ*-lactone ring to the benzene moiety via C-5a and C-9a. HMBC correlations between H-4 and the carbonyl carbon at *δ*_C_ 165.2 (C-3), as well as between the methoxy signal and another carbonyl carbon at *δ*_C_ 165.8 (C-1) were observed. These correlations indicated that a carboxylic acid group is located at C-3a, with a methoxycarbonyl group accordingly attached to C-9b. The complete structure was fully confirmed through 2D NMR analysis. On this basis, the structure of compound **9** was identified and given the name marasmielolic acid E.

Compound **10** was found to have the molecular formula of C_15_H_14_O_6_, as evidenced by the positive HR-ESI-MS data (*m/z* 313.0680 [M+Na]^+^, calcd. 313.0683), which implies an unsaturation degree of nine. In the ^1^H NMR spectrum of compound **10** (measured in DMSO-*d*₆, Table 4), two extremely broad singlets were detected at *δ*_H_ 13.50 (1 H, vbr s, COOH) and 8.96 (1 H, vbr s, 8-OH). Additionally, there were two aromatic doublets with mutual coupling at *δ*_H_ 8.17 (1 H, d, *J* = 8.4 Hz, H-4) and 8.00 (1 H, d, *J* = 8.4 Hz, H-5), a singlet for an olefinic proton at *δ*_H_ 6.23 (1 H, s, H-7), a methoxy signal at *δ*_H_ 3.82 (3 H, s), and two methyl singlets at *δ*_H_ 1.48 (6 H, s). The ^13^C NMR spectrum (Table 4) displayed 15 carbon resonance signals, including an aromatic ketone carbonyl carbon at *δ*_C_ 179.3 (s, C-9), two aromatic carboxyl or ester carbonyl carbons at *δ*_*C*_ 168.6 (C-1) and 165.8 (C-3), and eight aromatic or olefinic carbons that could be attributed to a benzene ring and a double bond group. Also present were a methyl ester carbon at *δ*_C_ 52.1, an aliphatic quaternary carbon at *δ*_C_ 37.6 (C-6), and two methyl carbons at *δ*_C_ 30.0. Analysis of the degrees of unsaturation revealed the existence of one aliphatic ring in the structure. The HMBC correlations (Figure 2) from H-5 to the aliphatic quaternary carbon at *δ*_C_ 37.6 (C-6), from the two methyl singlets to C-5a (*δ*_C_ 155.3) and the olefinic carbon at *δ*_C_ 128.1 (C-7), and from the olefinic proton singlet at *δ*_H_ 6.23 (1 H, s, H-7) to C-5a, C-6, C-8, and the aromatic ketone carbon at *δ*_C_ 179.3 (C-9), confirmed the presence of a 2-hydroxy-4,4-dimethylnaphthalen-1(4*H*)-one moiety. Moreover, the substituents of a carboxylic acid at C-3a and a methoxycarbonyl at C-9b were established through analysis of HMBC correlations, especially the weak but valuable four-bond correlation from the methoxy signal to C-9b. Finally, the structure of **10** was confirmed and given the name marasmielolic acid F. Given the presence of the cyclohexanone moiety, this structure may be the precursor of **1**‒**9**.

**Figure 4.**
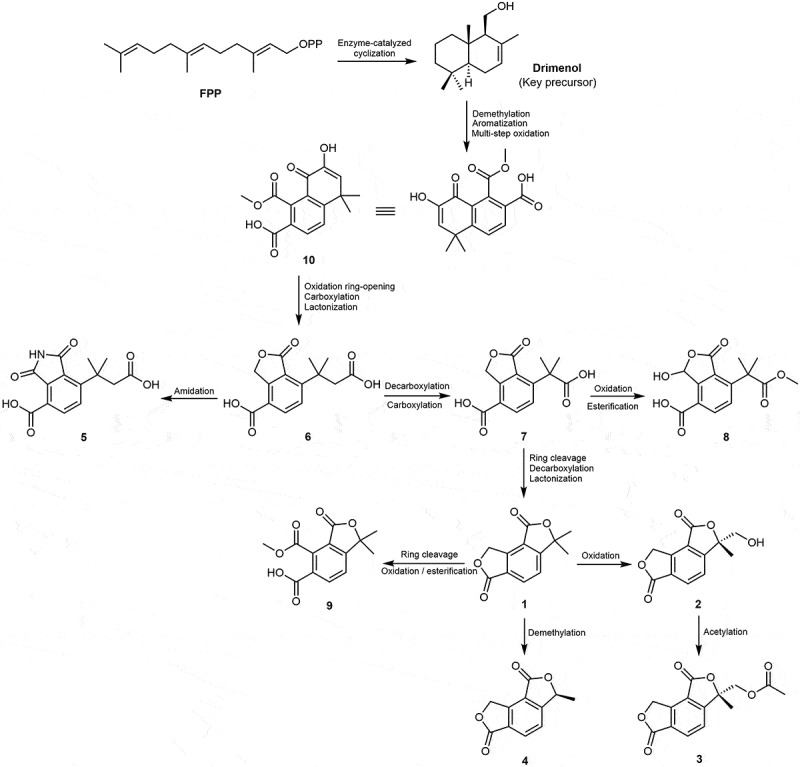
The possible biosynthetic pathways of **1**−**10**.

After searching the similar literature and the SciFinder database, compounds **1**–**10** were identified as new compounds. The biosynthetic pathways of compounds **1**–**10** are proposed to start from farnesyl pyrophosphate (FPP), which undergoes enzyme-catalyzed cyclization to form the key sesquiterpene precursor drimenol. Followed by sequential transformations including demethylation, aromatization, oxidation, lactonization, ring cleavage, and carboxylation, which collectively generate their diverse structures (Figure 4). These diverse modifications reflect the metabolic versatility of *M. candidus* in producing structurally varied secondary metabolites from a common precursor.

The isolated compounds were assessed for their anti-inflammatory properties in RAW264.7 macrophages through the measurement of NO production. The results shown in Table 5, all isolated compounds do not exhibit any cytotoxicity in RAW 264.7 cells at concentrations of 80 µmol/L. Compounds **1**−**4** exhibited good anti-inflammatory activity with IC_50_ values of 18.2, 16.7, 22.1, and 15.5 μmol/L, respectively. Compounds **5**−**8** also exhibited a certain inhibitory effect with IC_50_ values ranging from 50.8 to 69.2 μmol/L. In terms of structure-activity relationships, preliminary analysis reveals that compounds **1**−**4**, which exhibit the most significant anti-inflammatory activity, share a core isobenzofuran-3(1*H*)-one moiety and a fused *γ*-lactone ring, suggesting that this structural framework may be a key feature for their anti-inflammatory properties. In contrast, compounds **5**−**10**, which have undergone structural modifications such as lactone ring cleavage, show significantly decreased anti-inflammatory activity, implying that the integrity of the lactone ring system and the type of substituents at specific positions play important roles in modulating their biological activity. These findings provide valuable clues for further structural optimization of this class of compounds.

**Table 5.** NO inhibition activity of compounds **1**−**10**.

Compounds	IC_50_ (μmol/L)	Cytotoxicity (μmol/L)
1	18.2 ± 1.9	> 80
2	16.7 ± 1.6	> 80
3	22.1 ± 2.6	> 80
4	15.5 ± 1.4	> 80
5	53.5 ± 3.9	> 80
6	65.4 ± 4.6	> 80
7	69.2 ± 3.1	> 80
8	50.8 ± 3.4	> 80
9	＞100	> 80
10	＞100	> 80
Aminoguanidine^a^	12.5 ± 1.8	> 80

^a^ aminoguanidine was used as a positive control for NO inhibition activity. Values are shown as mean ± standard deviation (*n* = 3).

## 4. Conclusions

In conclusion, ten novel norsesquiterpenoids, named marasmiellactones R−U (**1**−**4**) and marasmielolic acids A−F (**5**−**10**) were extracted from the cultures of *M. candidus*. The structural identification of these compounds was accomplished via comprehensive spectroscopic analyses. Importantly, compounds **1**−**4** showed promising anti-inflammatory effects, making them potential candidates for further drug development and optimization. Besides, this study increases the variety of secondary metabolites in the fungus *M. candidus*, and lays the groundwork for the development and application of *M. candidus*.

## Supplementary Material

0818-Supplementary_material.docx
