# Supplementary material for: Ten previously undescribed norsesquiterpenoids from the fungus Marasmiellus candidus and their anti-inflammatory activities
Source: Mycology. 2025 Sep 12;17(2):531–42. doi: 10.1080/21501203.2025.2551650 (PMC13267044; doi:10.1080/21501203.2025.2551650)
Supplement: 0818-Supplementary_material.docx [file TMYC_A_2551650_SM3002.docx]

**Supplementary material**

Ten previously undescribed norsesquiterpenoids from the fungus *Marasmiellus candidus* and their anti-inflammatory activities

Lan Yao^a,b^, Shiyu Li^c^, Jinxiu Zhang^a^, Zhuang Li^a,*^ and Jianhua Lv^a,*^

^a^College of Life Sciences, Hebei Normal University, Shijiazhuang, China

^b^Institute of Biology, Hebei Academy of Science, Shijiazhuang, China

^c^Engineering Research Center of Chinese Ministry of Education for Edible and Medicinal Fungi, Jilin Agricultural University, Changchun, China

*Corresponding authors:

Jianhua Lv lvjianhua@hebtu.edu.cn

Zhuang Li lizhuang@hebtu.edu.cn

College of Life Sciences, Hebei Normal University, Shijiazhuang 050000, China


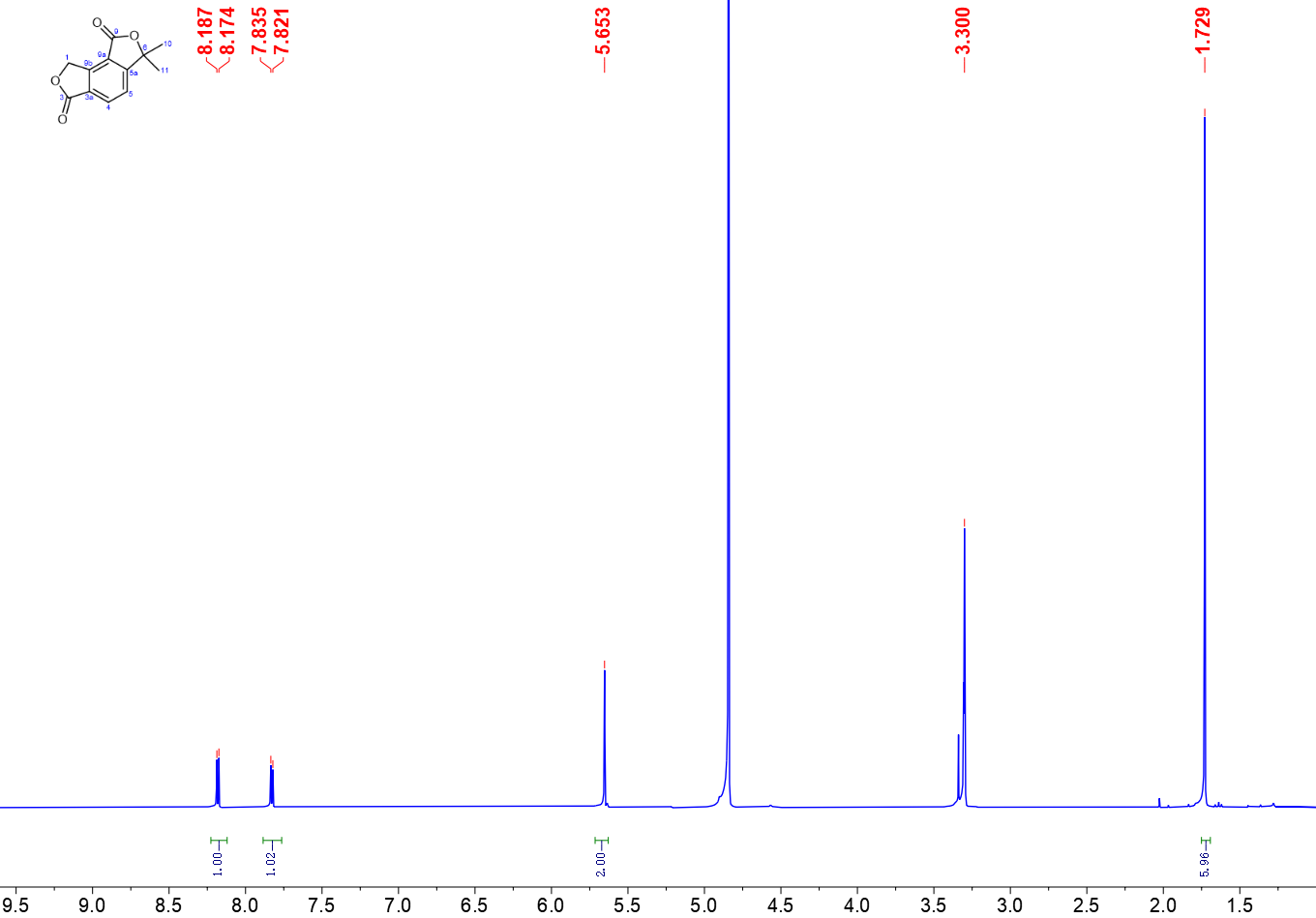


Figure S1. ^1^H NMR spectrum of **1.**


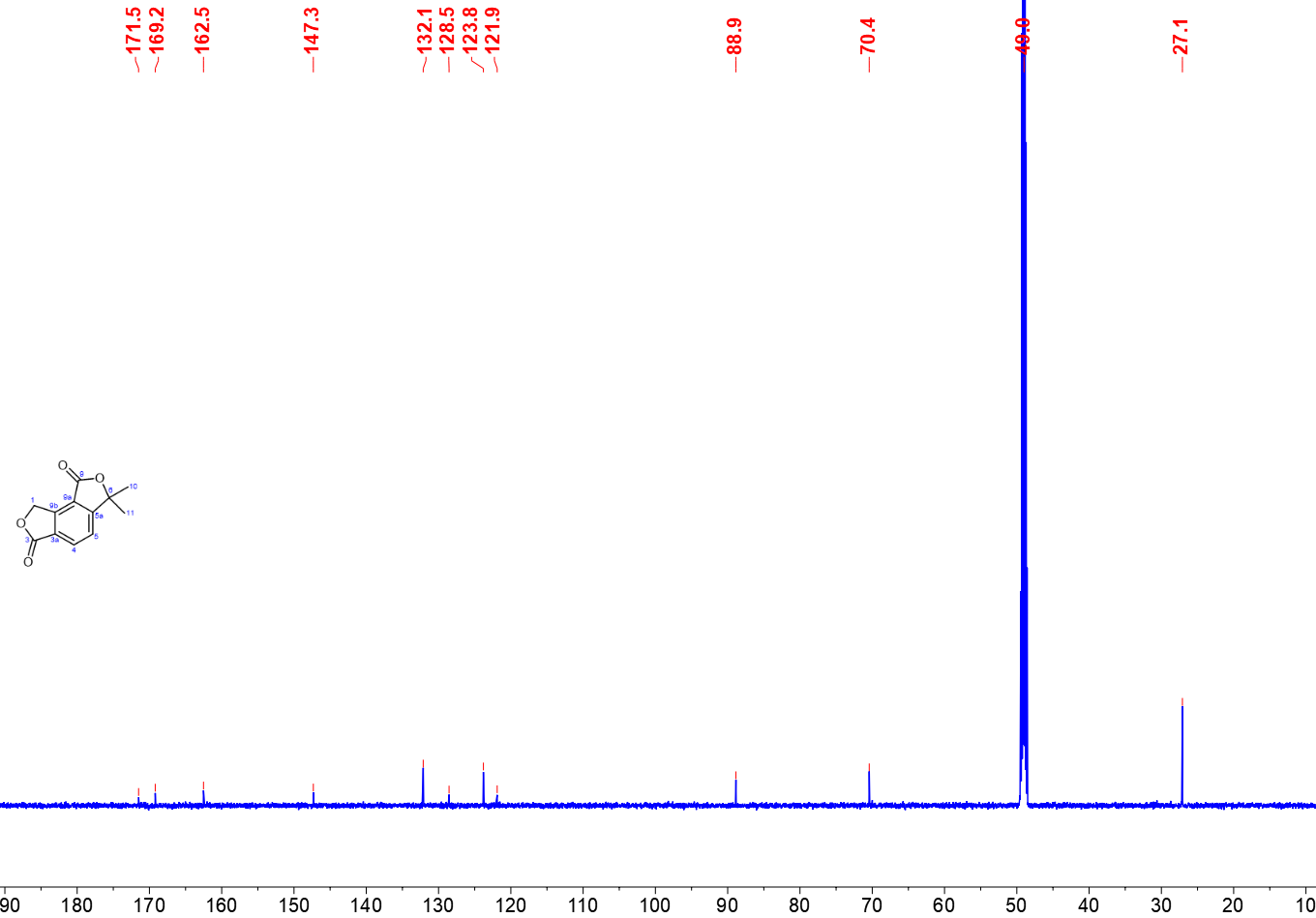
Figure S2. ^13^C NMR spectrum of **1.**


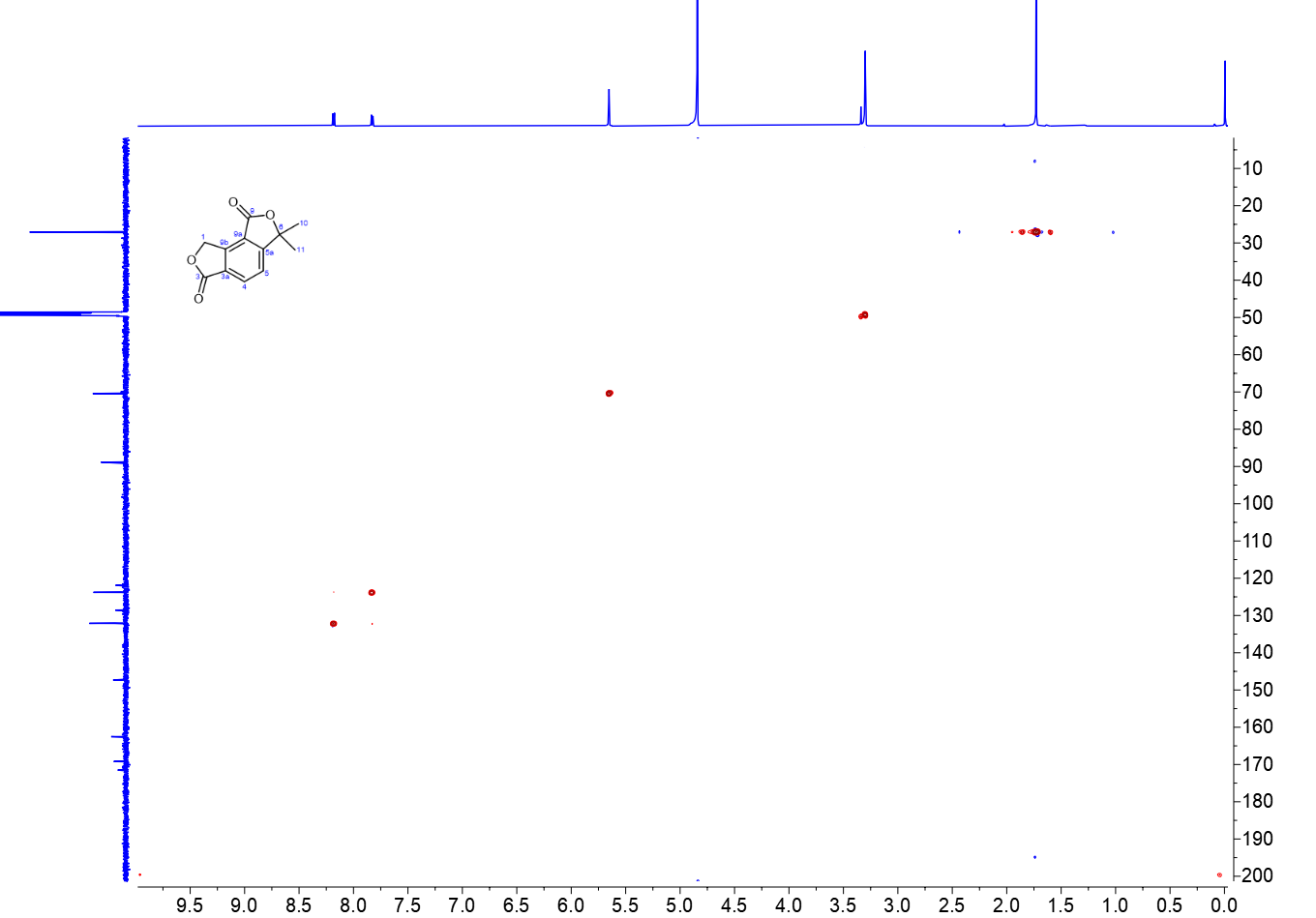
Figure S3. HSQC spectrum of **1.**


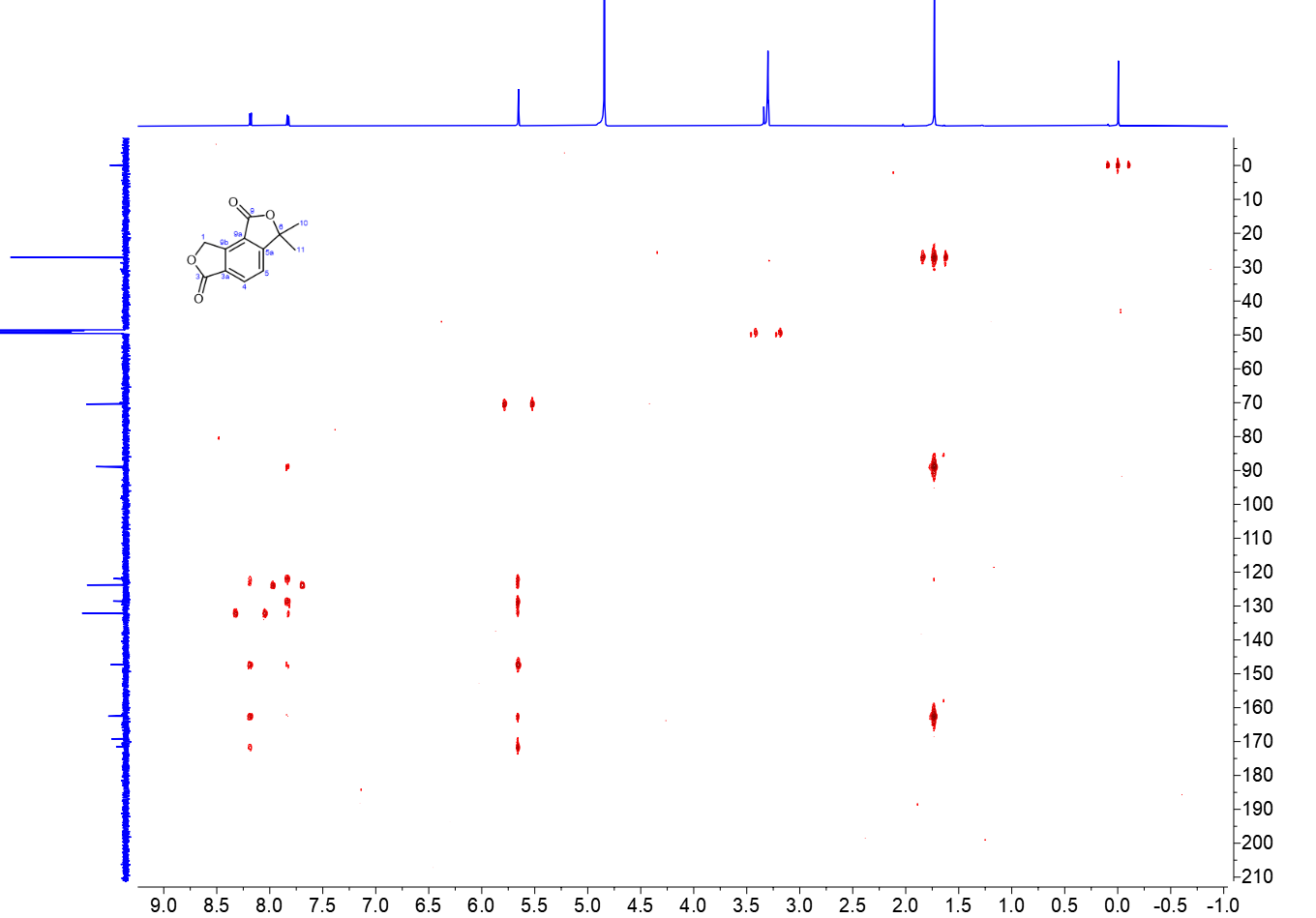
Figure S4. HMBC spectrum of **1.**


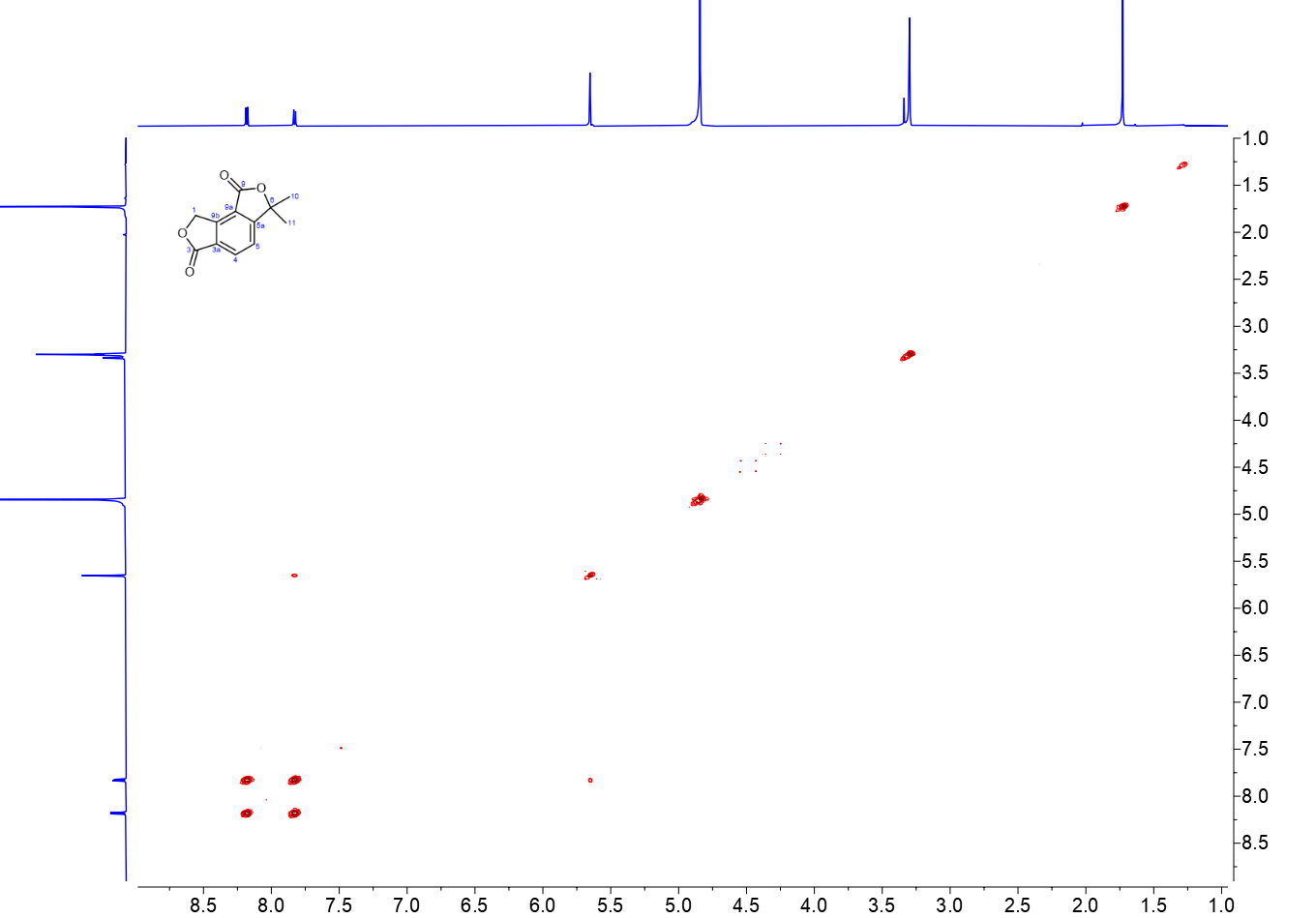
Figure S5. ^1^H-^1^H COSY spectrum of **1.**


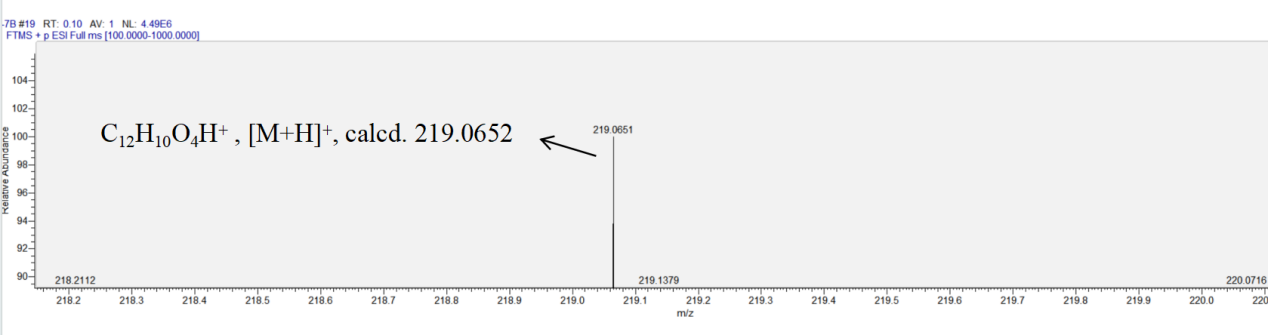


Figure S6. HRESIMS spectrum of **1.**





Figure S7. The UV spectrum of compound **1.**


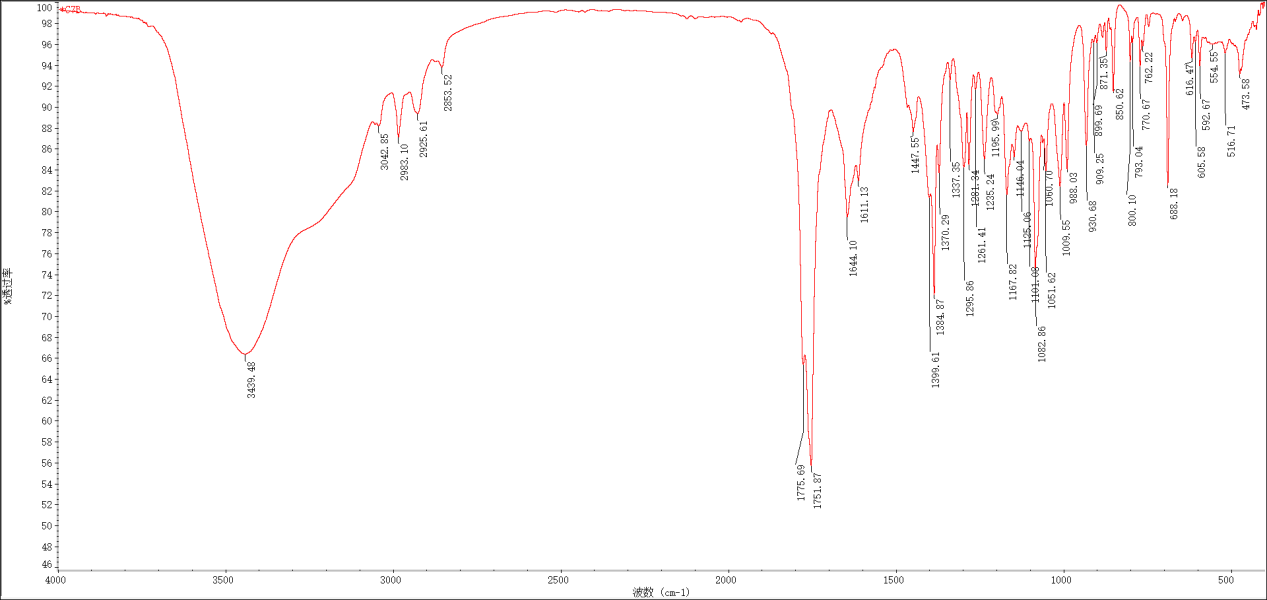
Figure S8. The IR spectrum of compound **1.**


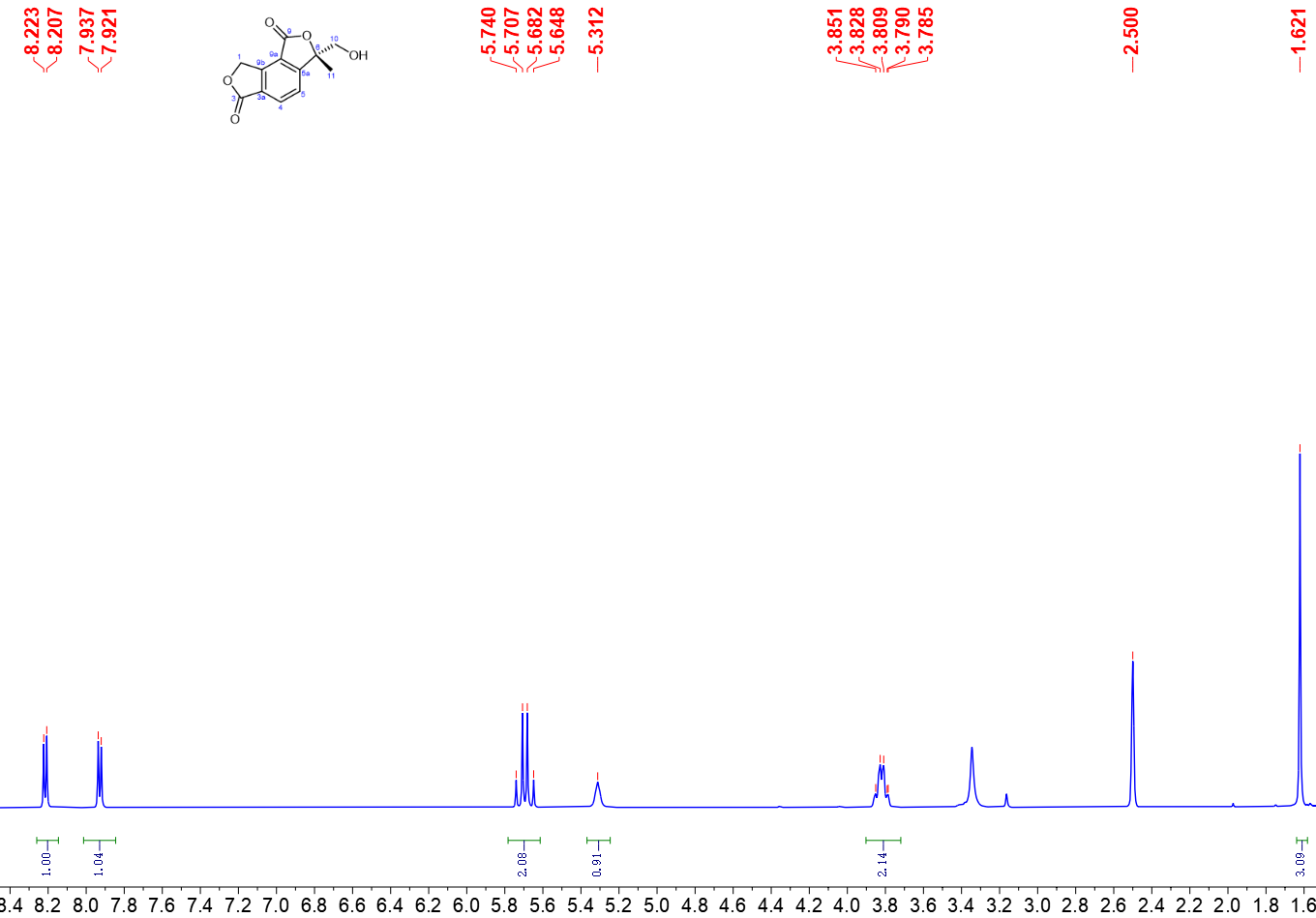


Figure S9. ^1^H NMR spectrum of **2.**


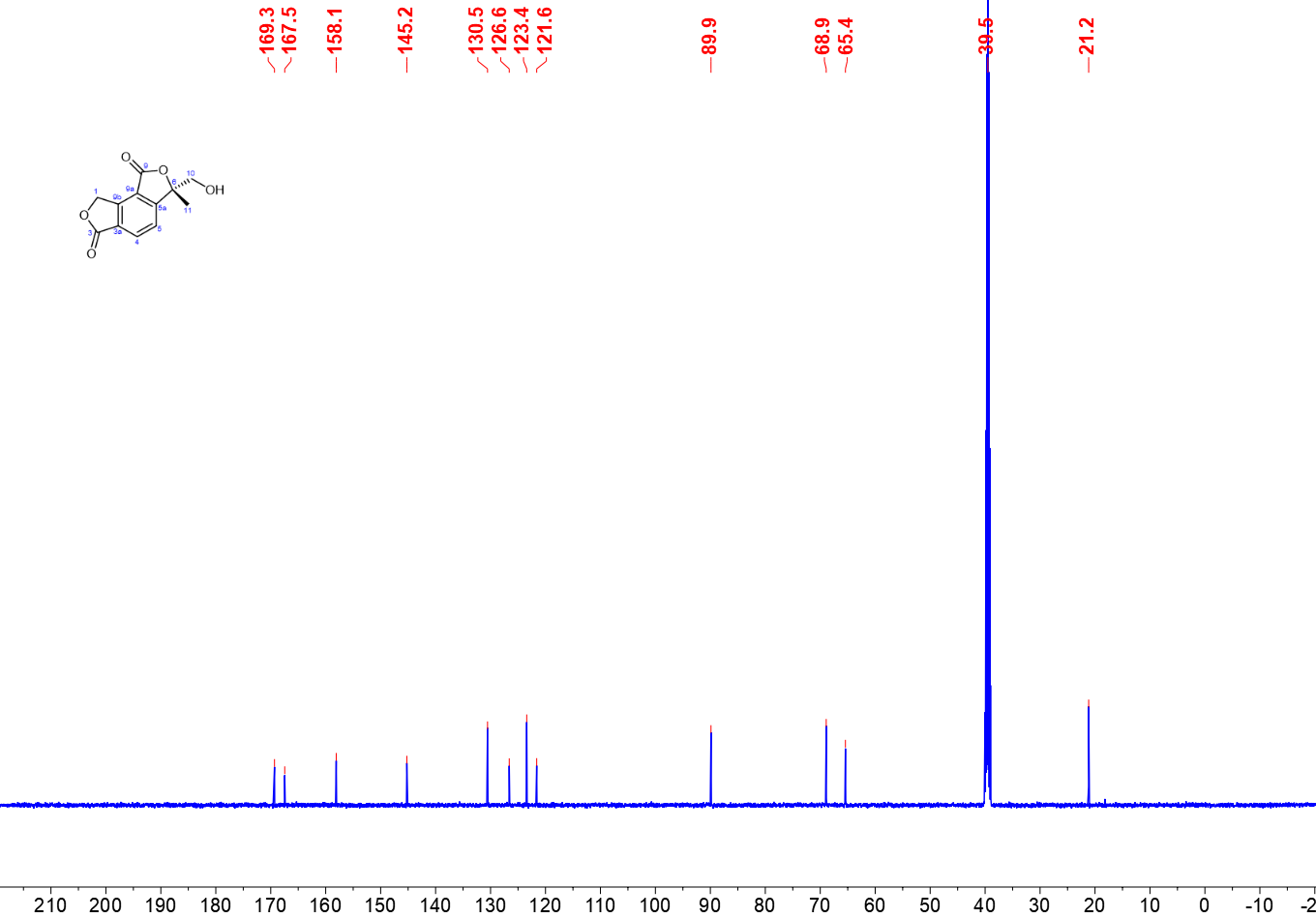
Figure S10. ^13^C NMR spectrum of **2.**


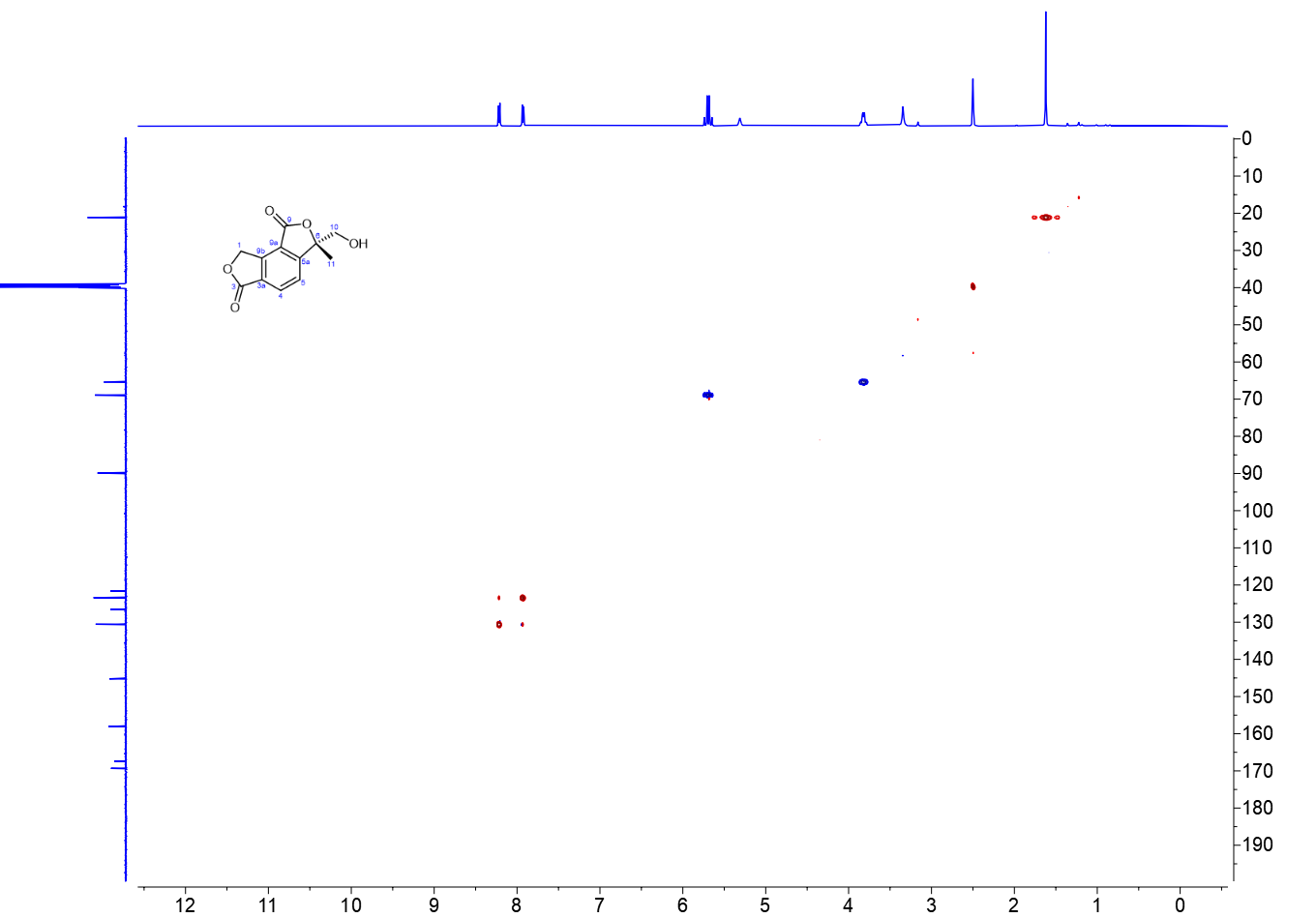
Figure S11. HSQC spectrum of **2.**


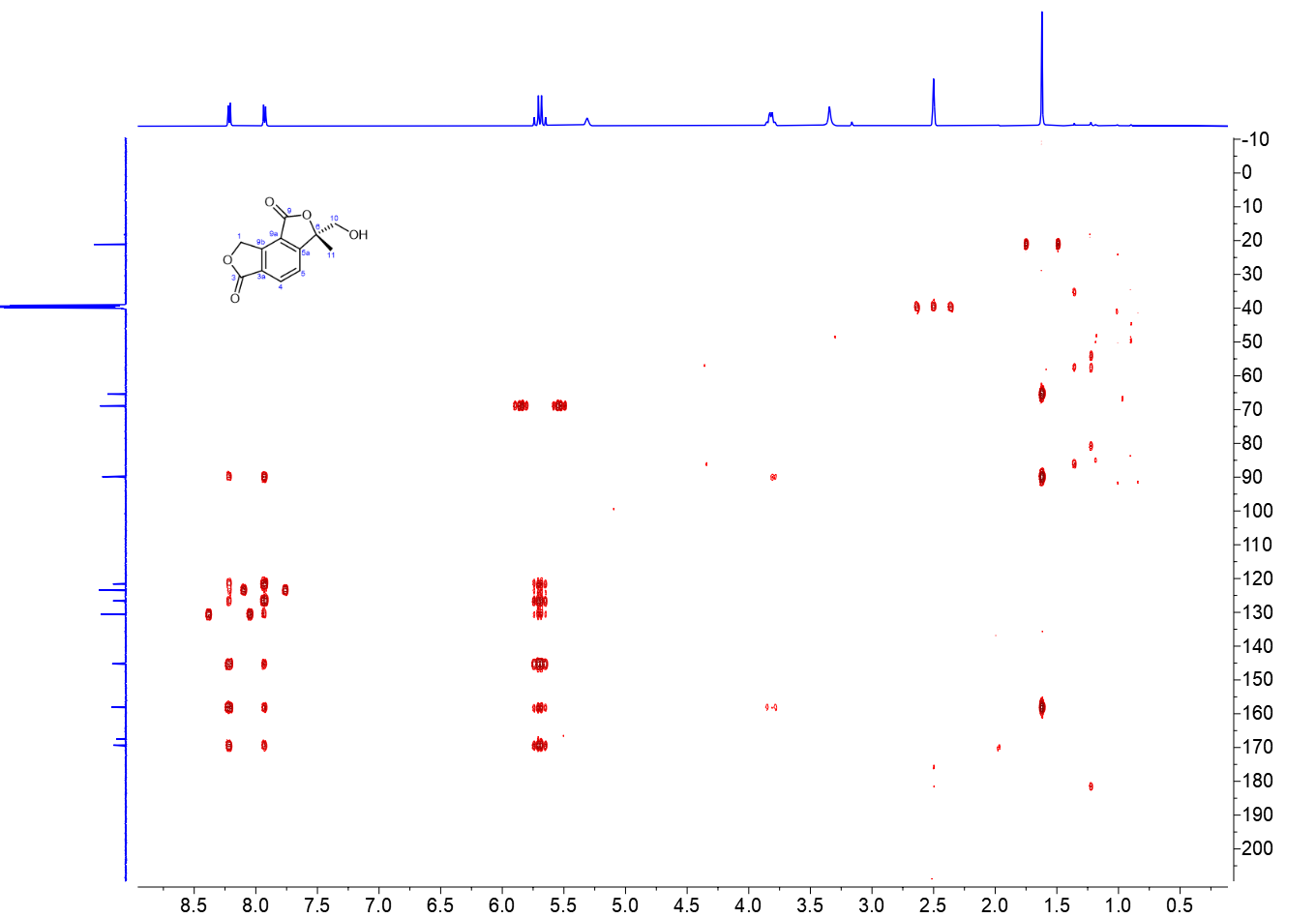
Figure S12. HMBC spectrum of **2.**


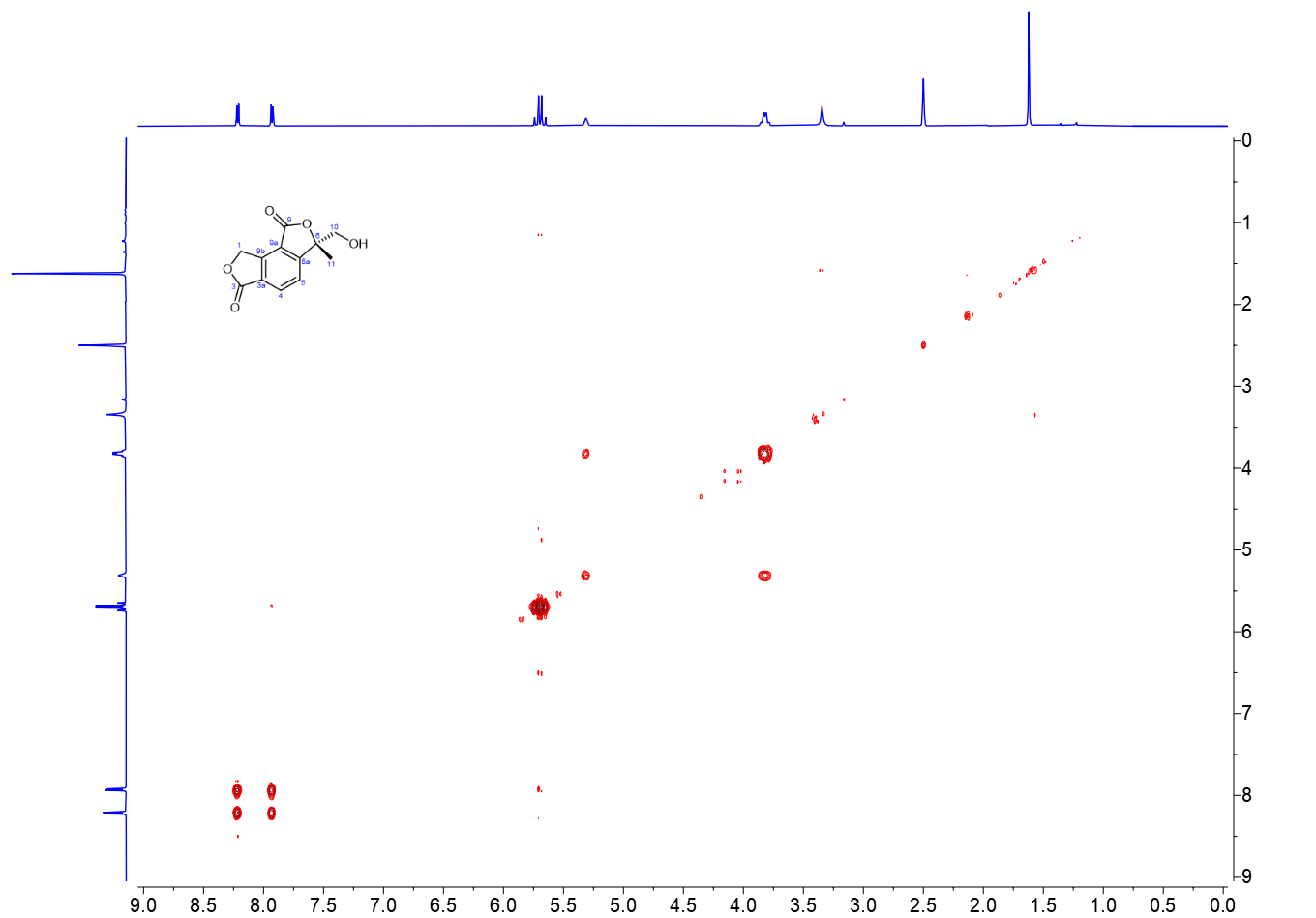
Figure S13. ^1^H-^1^H COSY spectrum of **2.**


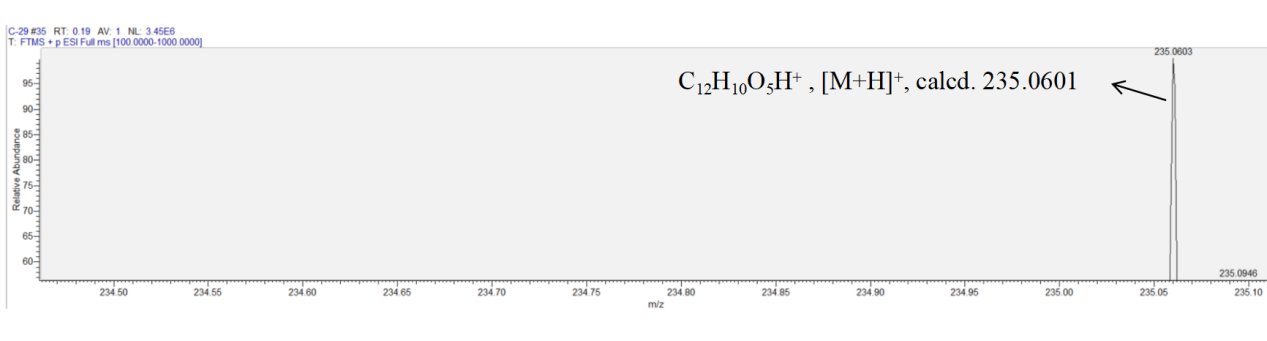
Figure S14. HRESIMS spectrum of **2.**





Figure S15. The UV spectrum of compound **2.**


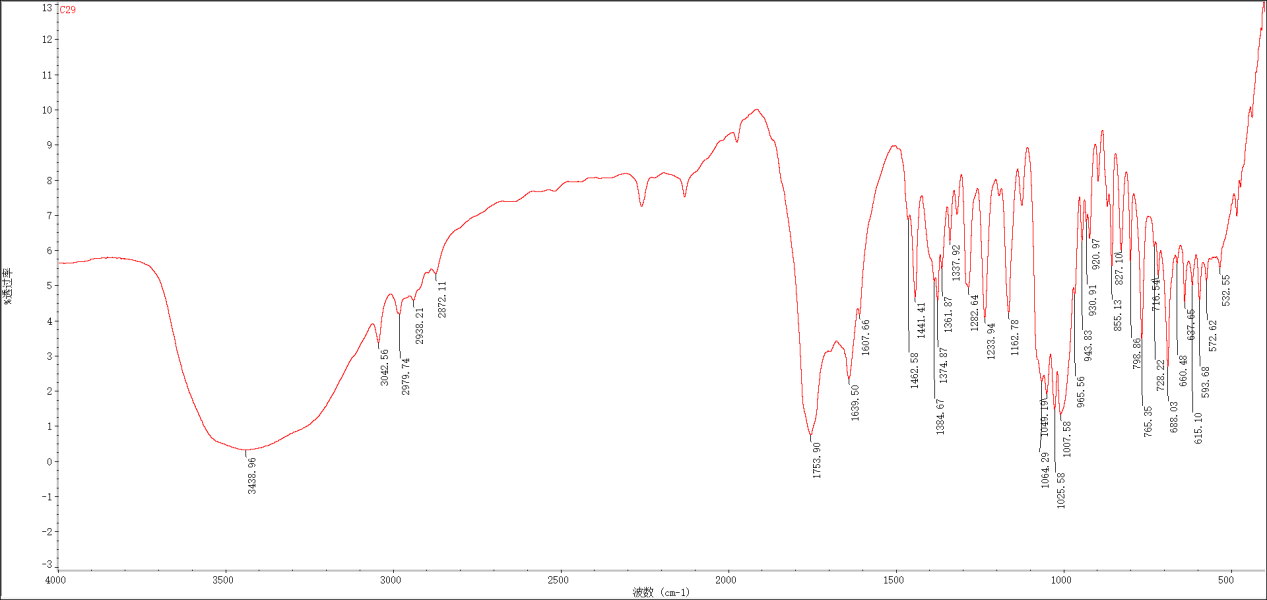
Figure S16. The IR spectrum of compound **2.**


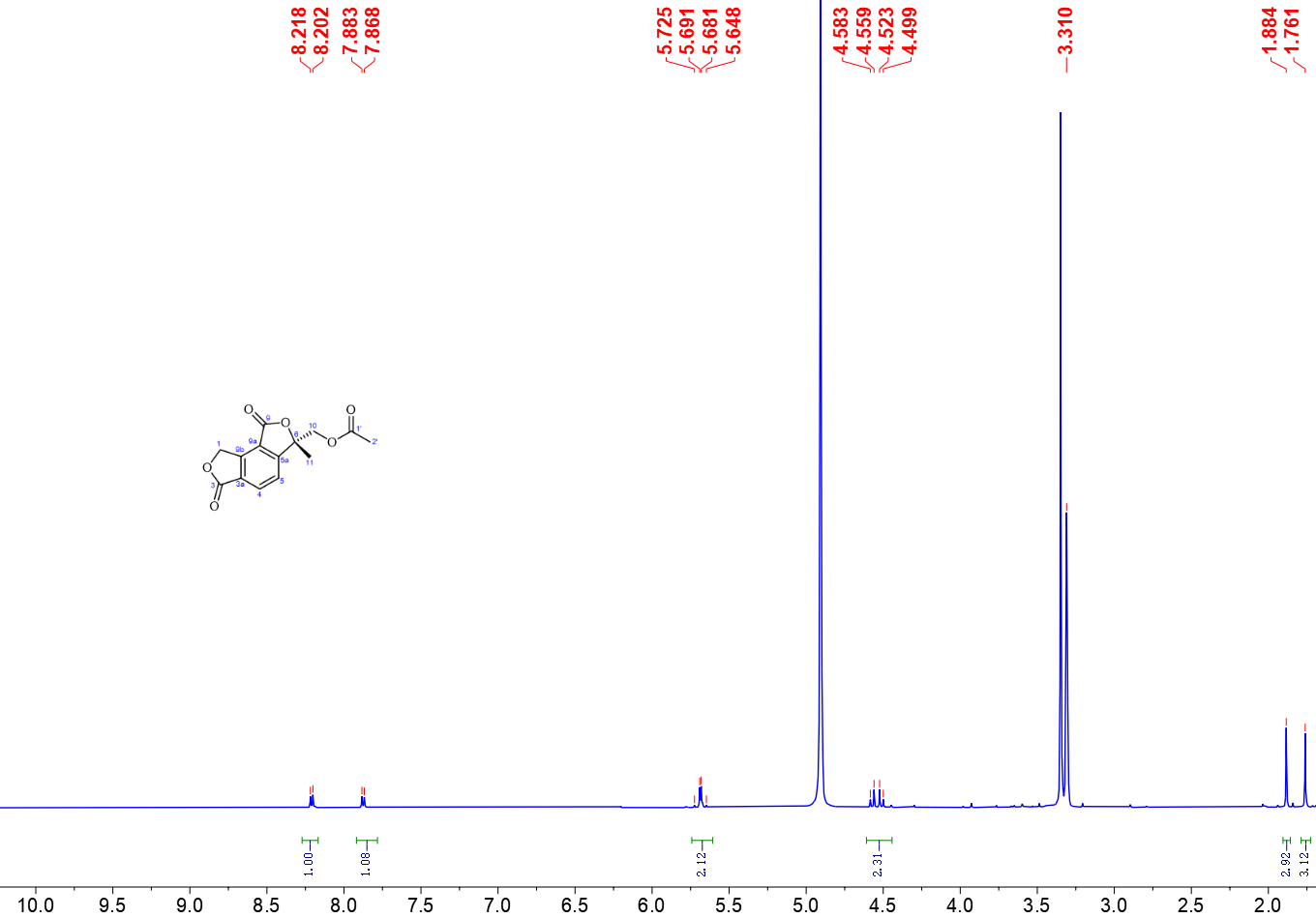
Figure S17. ^1^H NMR spectrum of **3.**


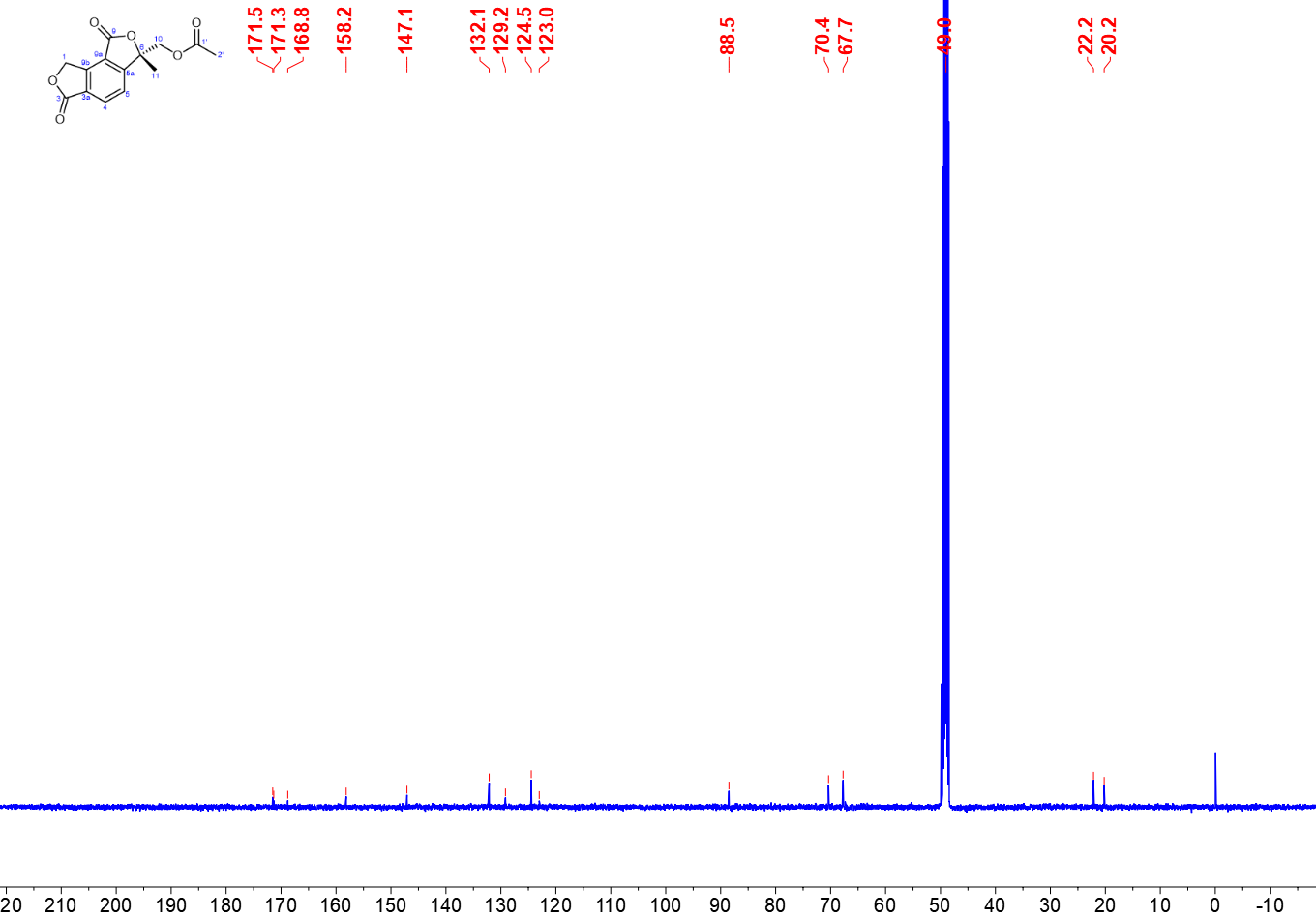
Figure S18. ^13^C NMR spectrum of **3.**


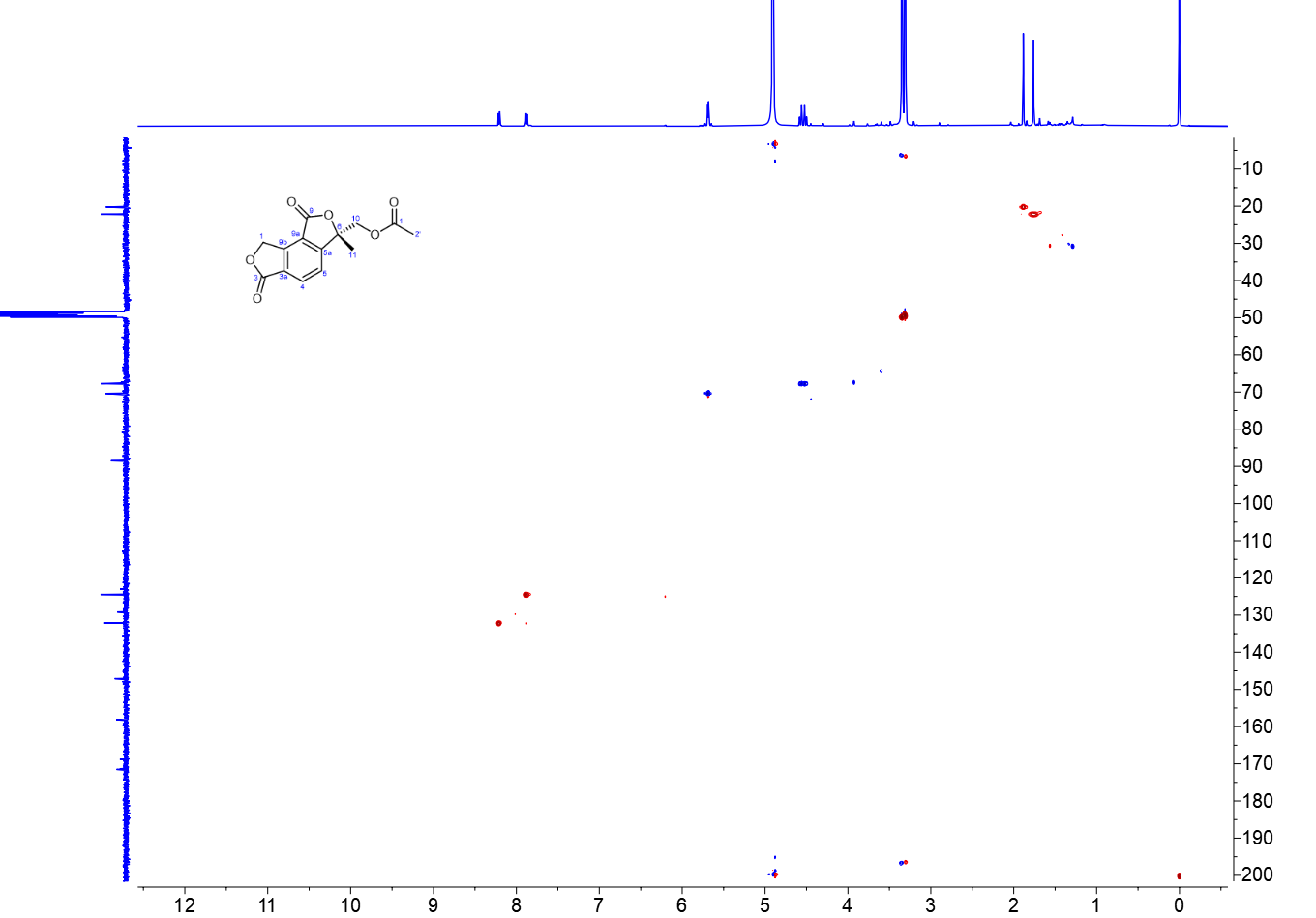
Figure S19. HSQC spectrum of **3.**


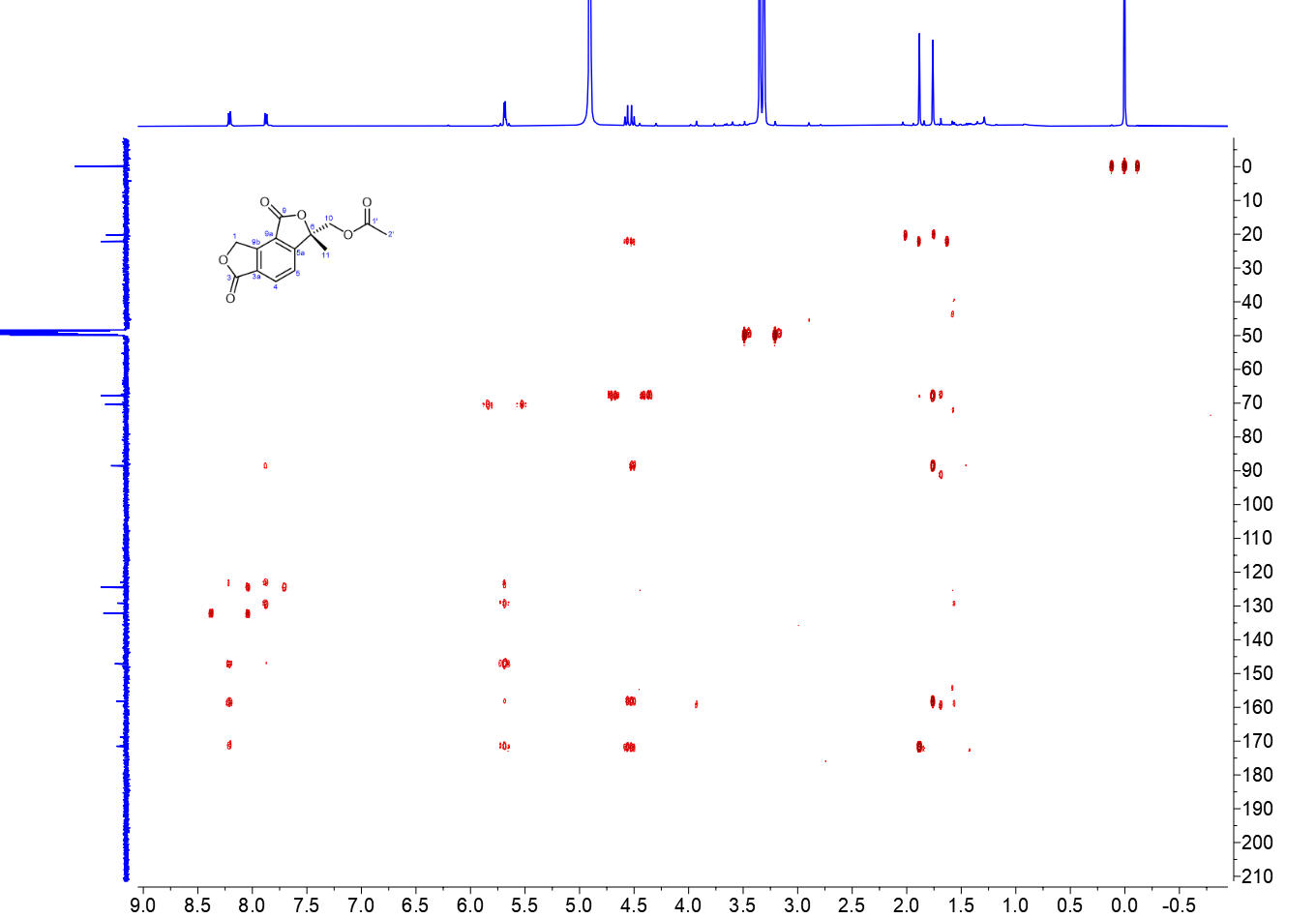
Figure S20. HMBC spectrum of **3.**


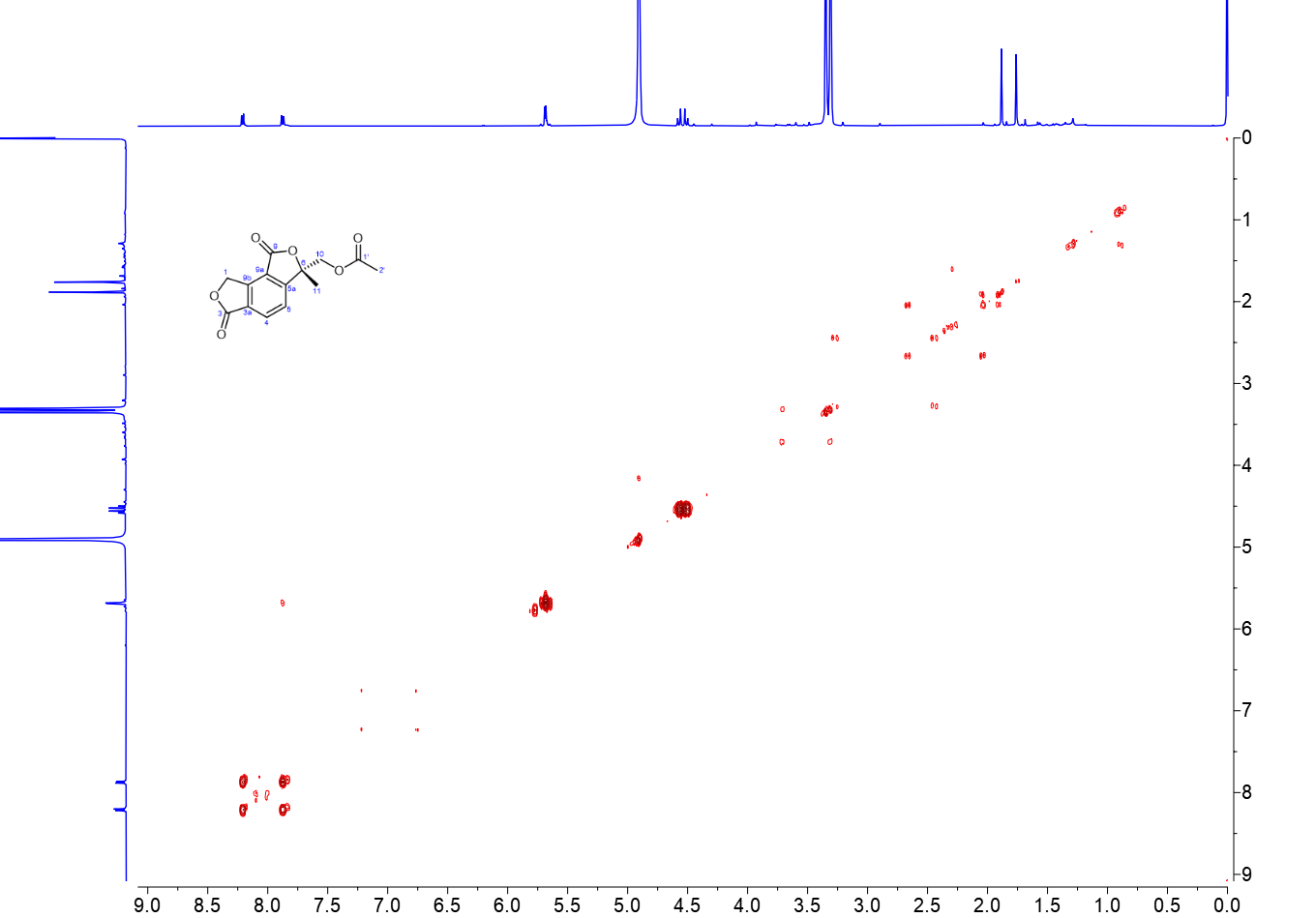
Figure S21. ^1^H-^1^H COSY spectrum of **3.**


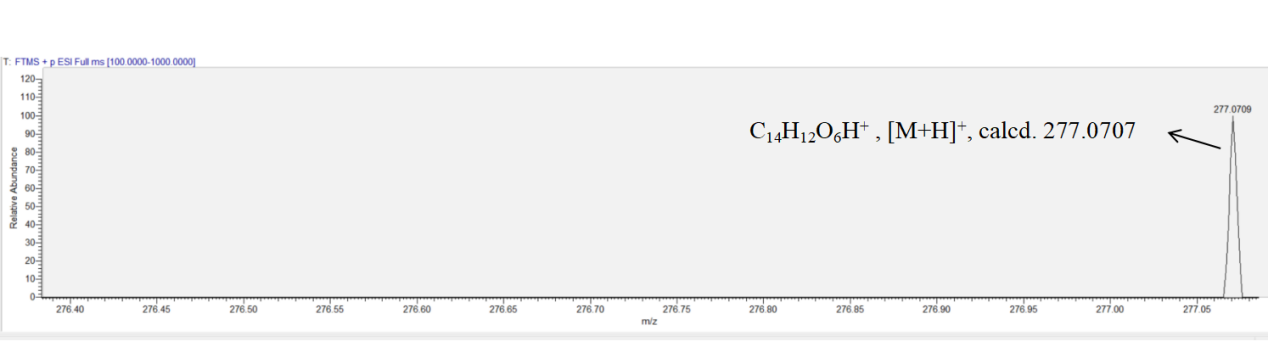


Figure S22. HRESIMS spectrum of **3.**





Figure S23. The UV spectrum of compound **3.**


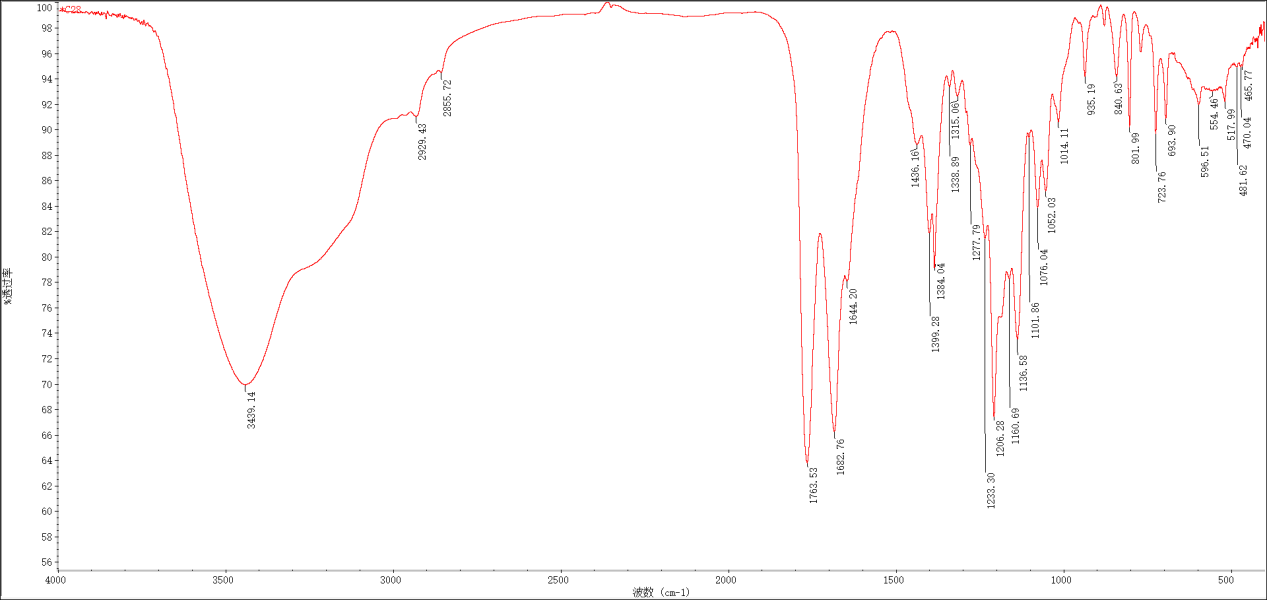
Figure S24. The IR spectrum of compound **3.**


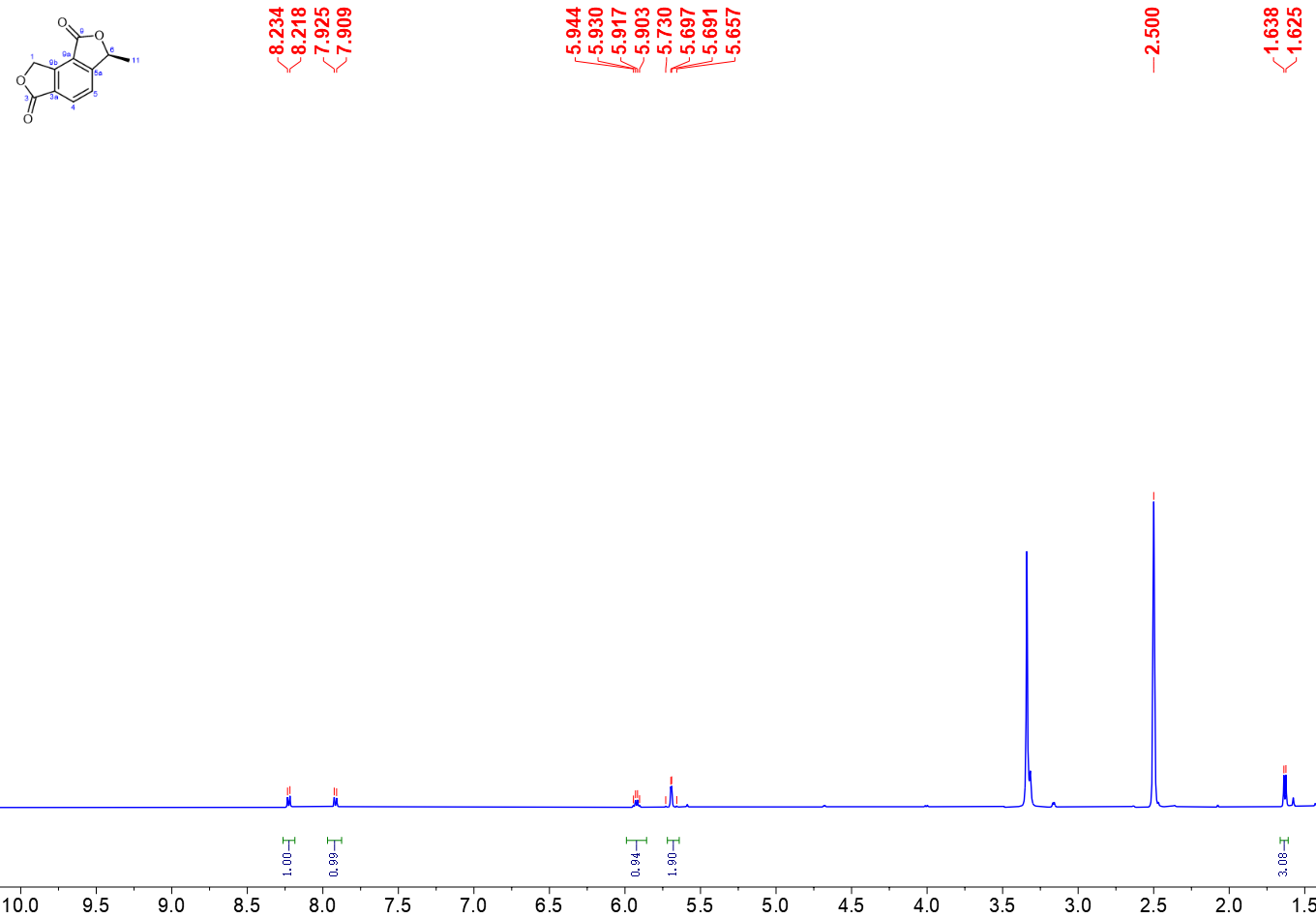


Figure S25. ^1^H NMR spectrum of **4.**


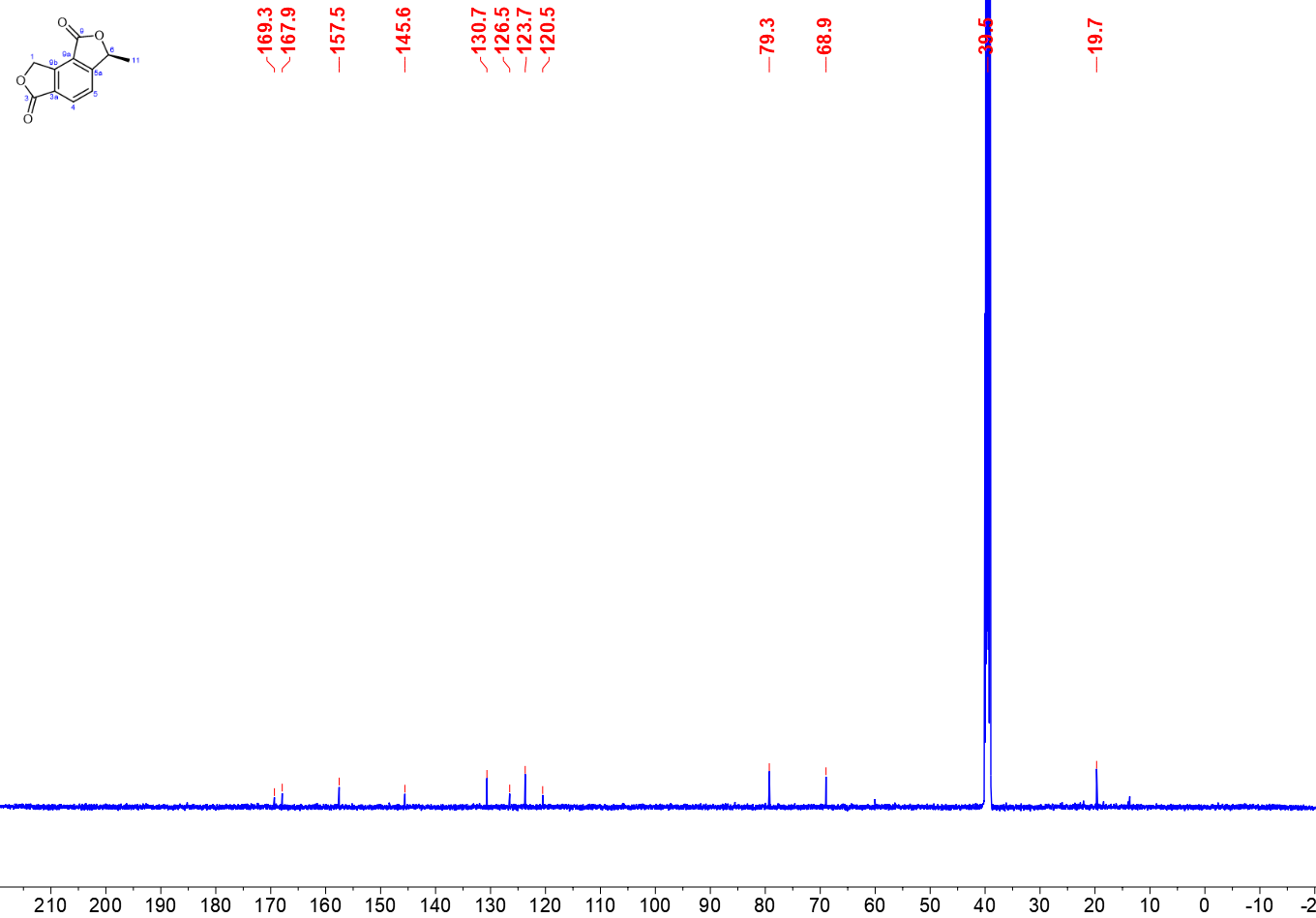
Figure S26. ^13^C NMR spectrum of **4.**


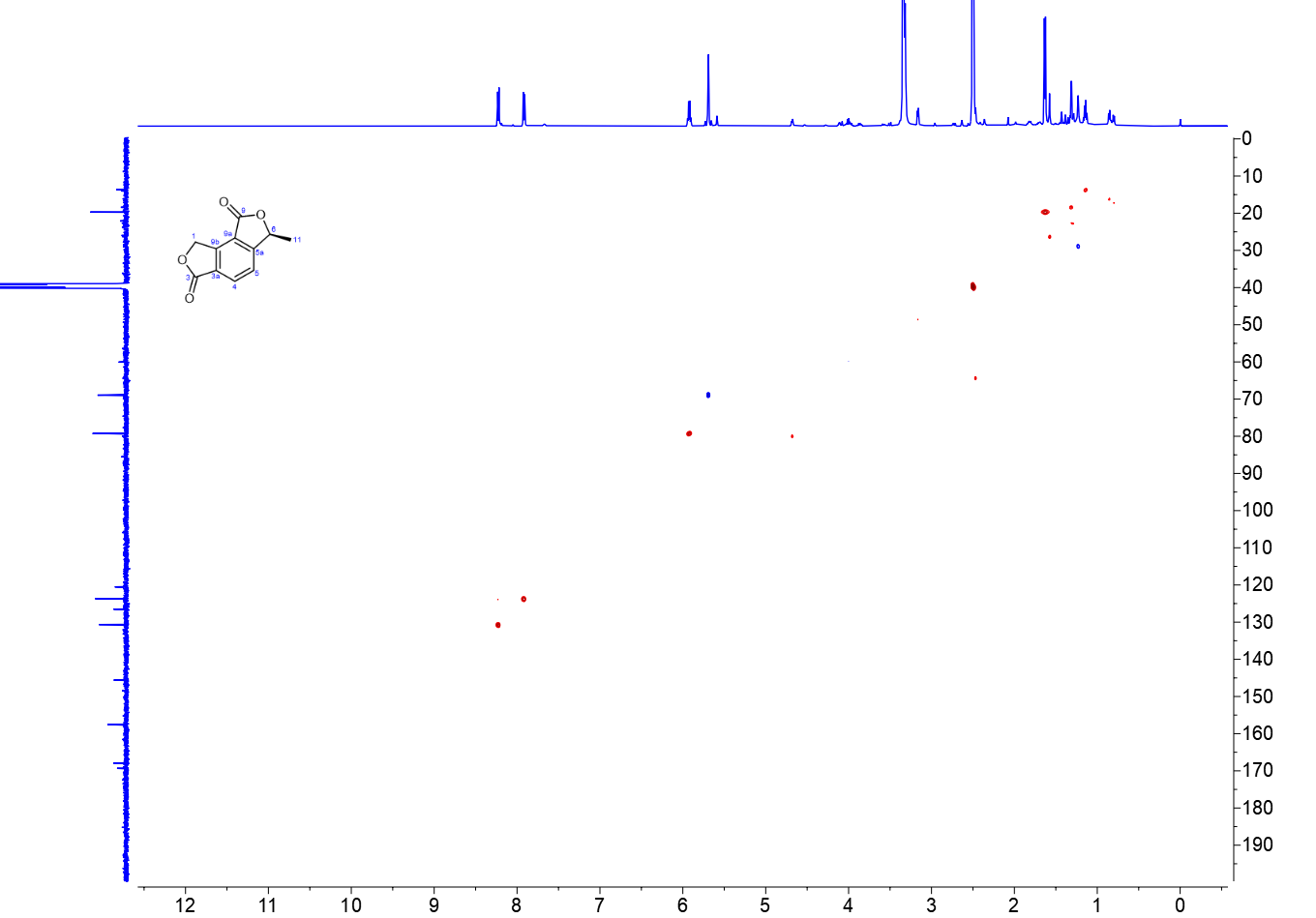
Figure S27. HSQC spectrum of **4.**


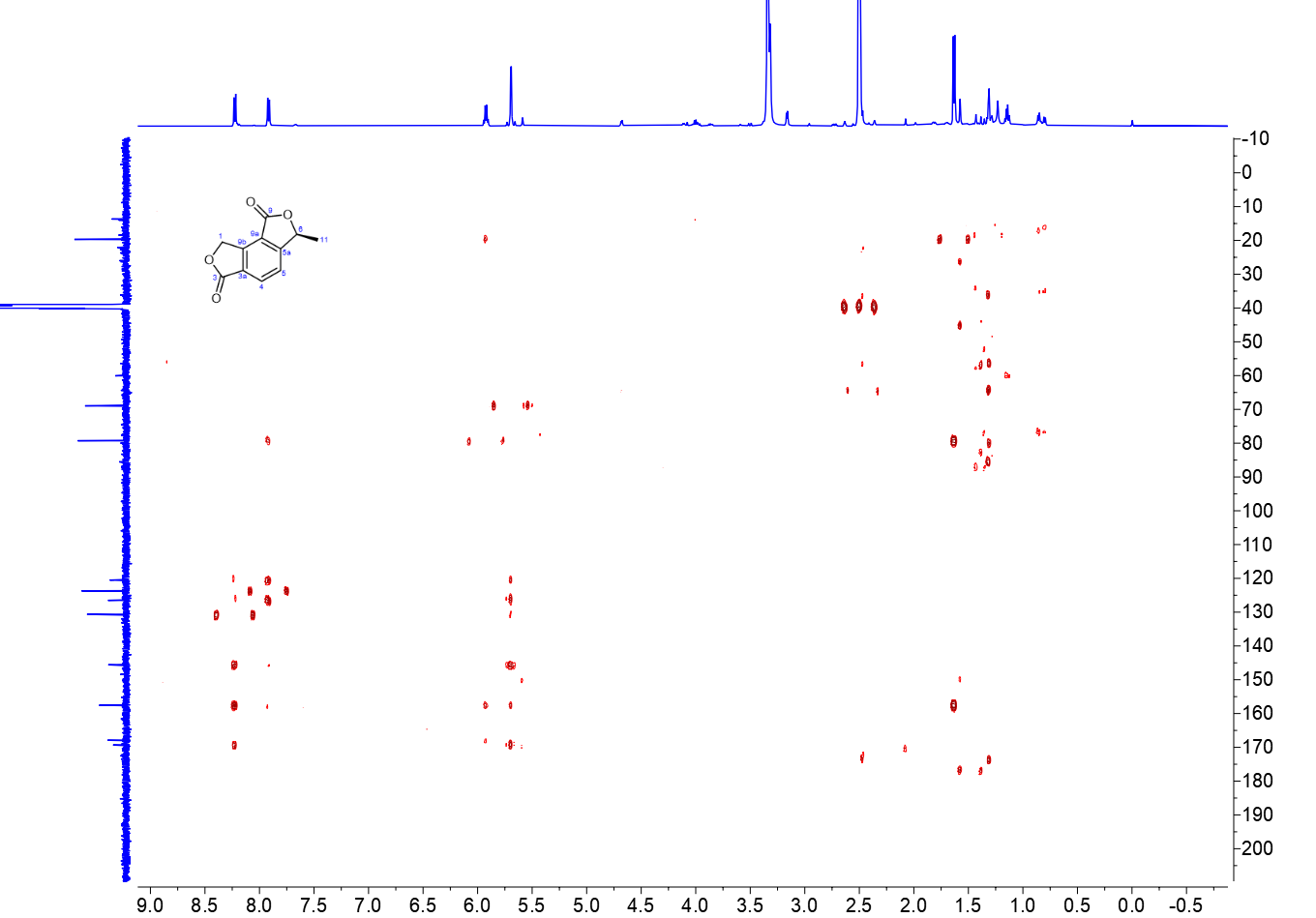
Figure S28. HMBC spectrum of **4.**


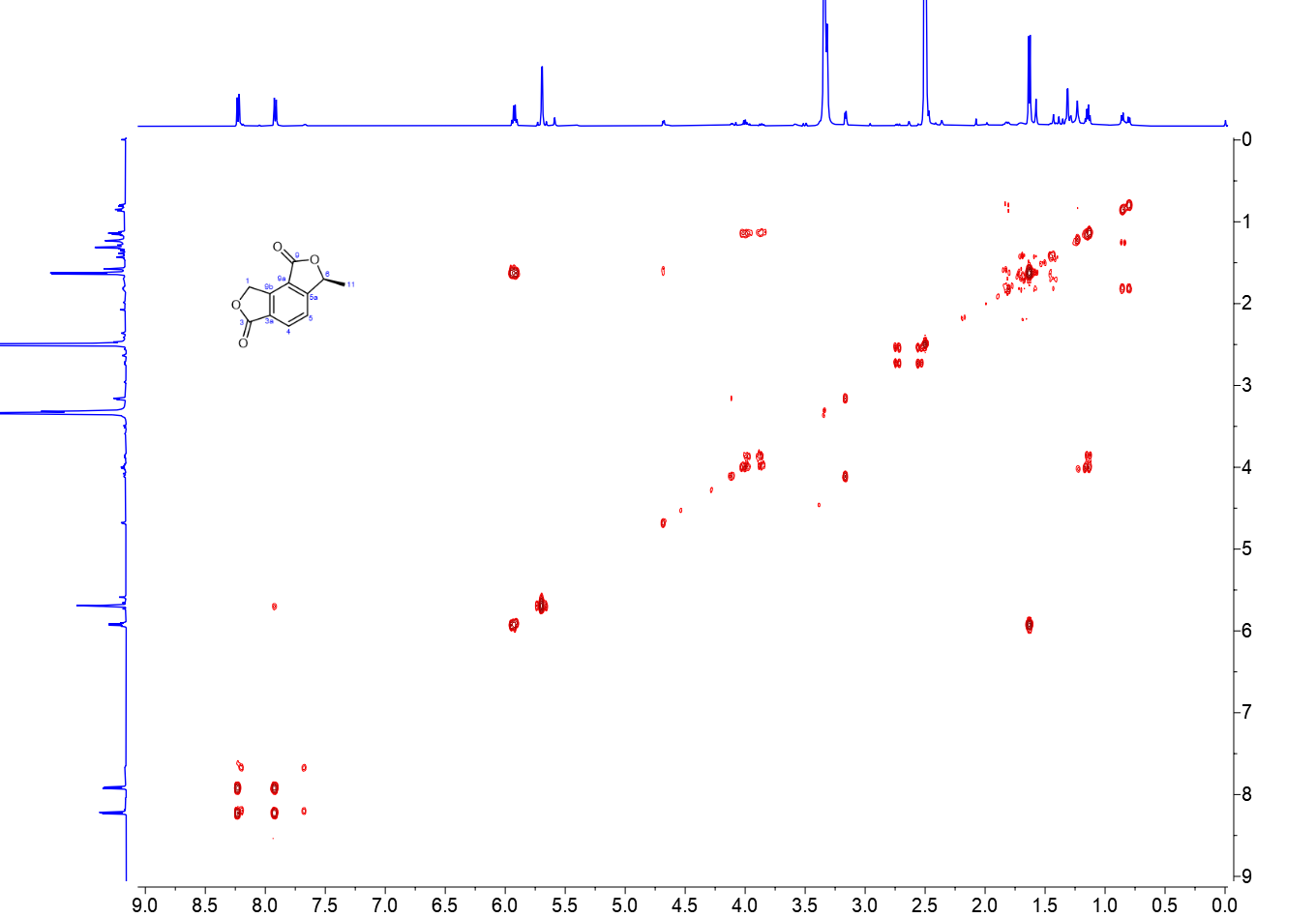
Figure S29. ^1^H-^1^H COSY spectrum of **4.**


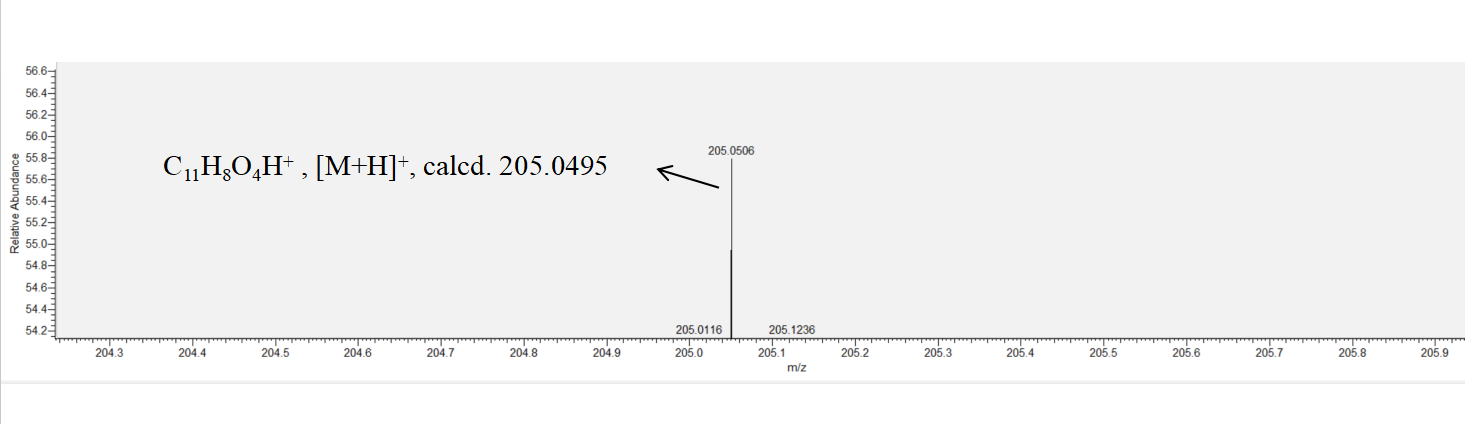
Figure S30. HRESIMS spectrum of **4.**





Figure S31. The UV spectrum of compound **4.**


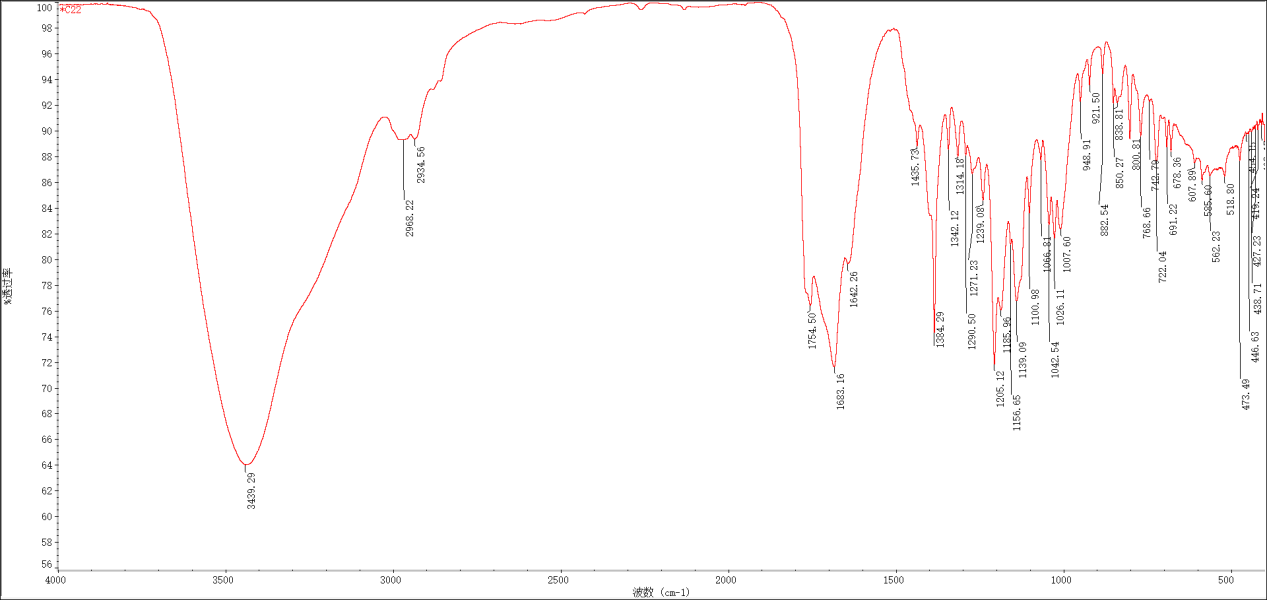
Figure S32. The IR spectrum of compound **4.**


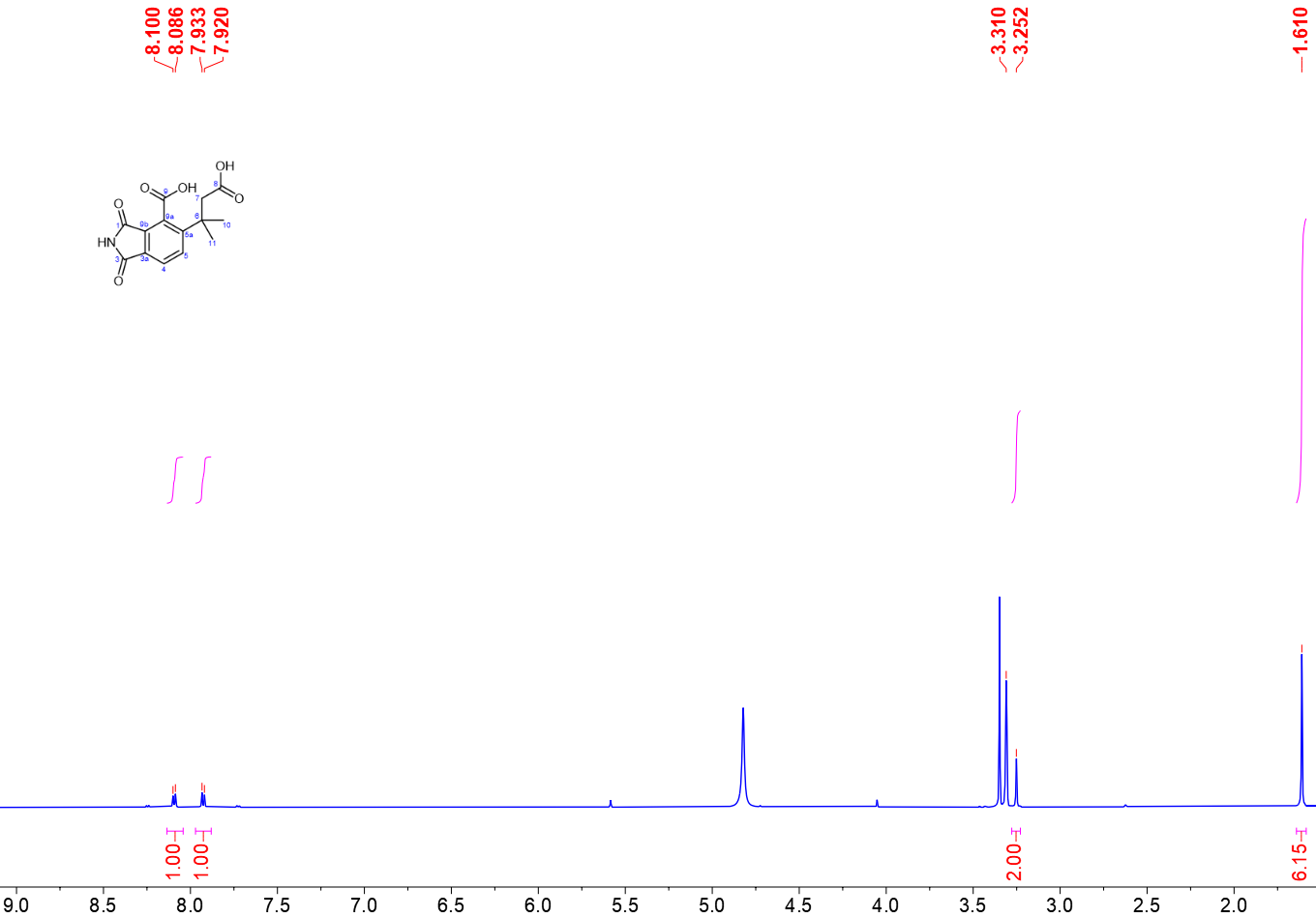


Figure S33. ^1^H NMR spectrum of **5** (CD_3_OD).


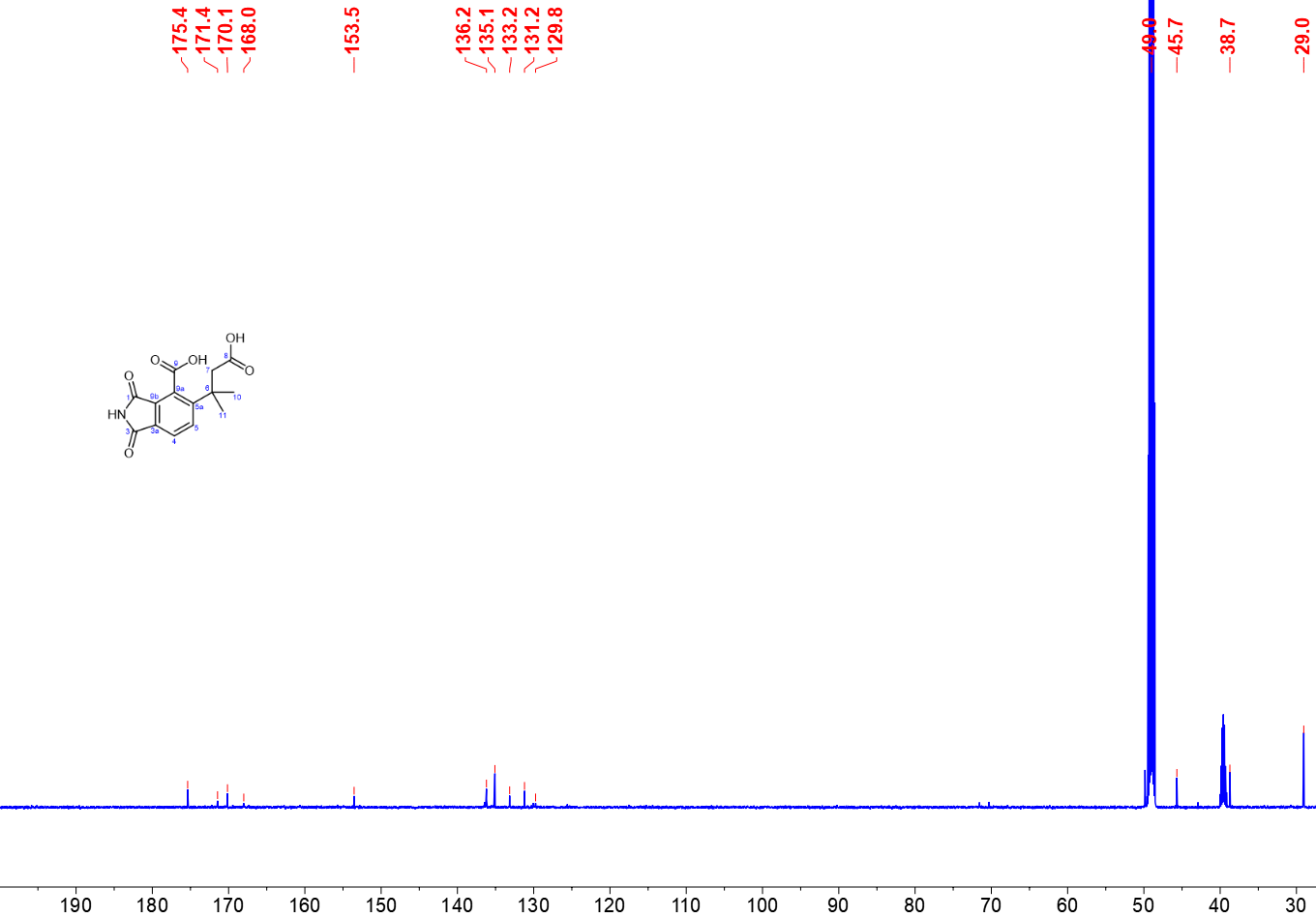
Figure S34. ^13^C NMR spectrum of **5** (CD_3_OD).


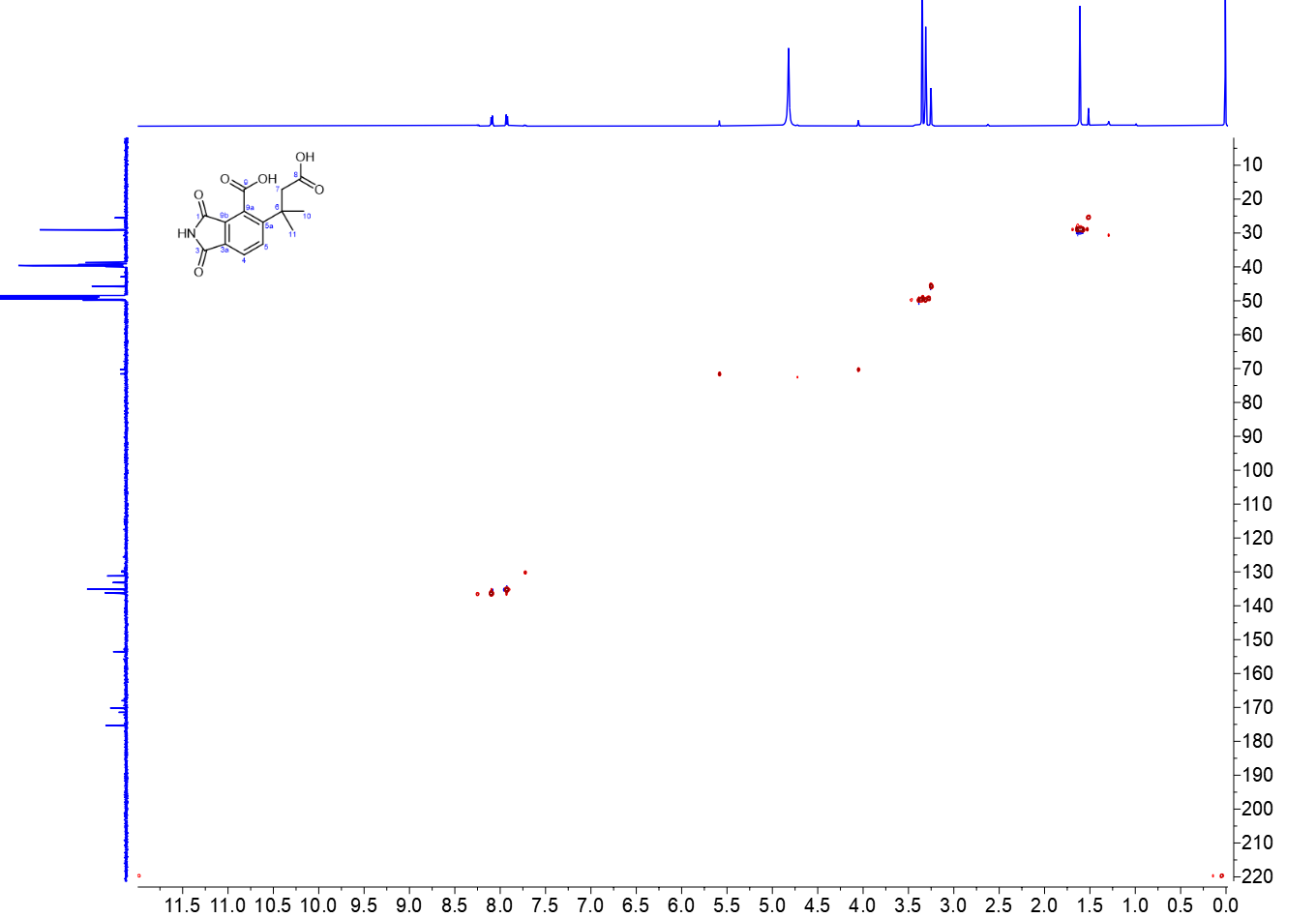
Figure S35. HSQC spectrum of **5** (CD_3_OD).


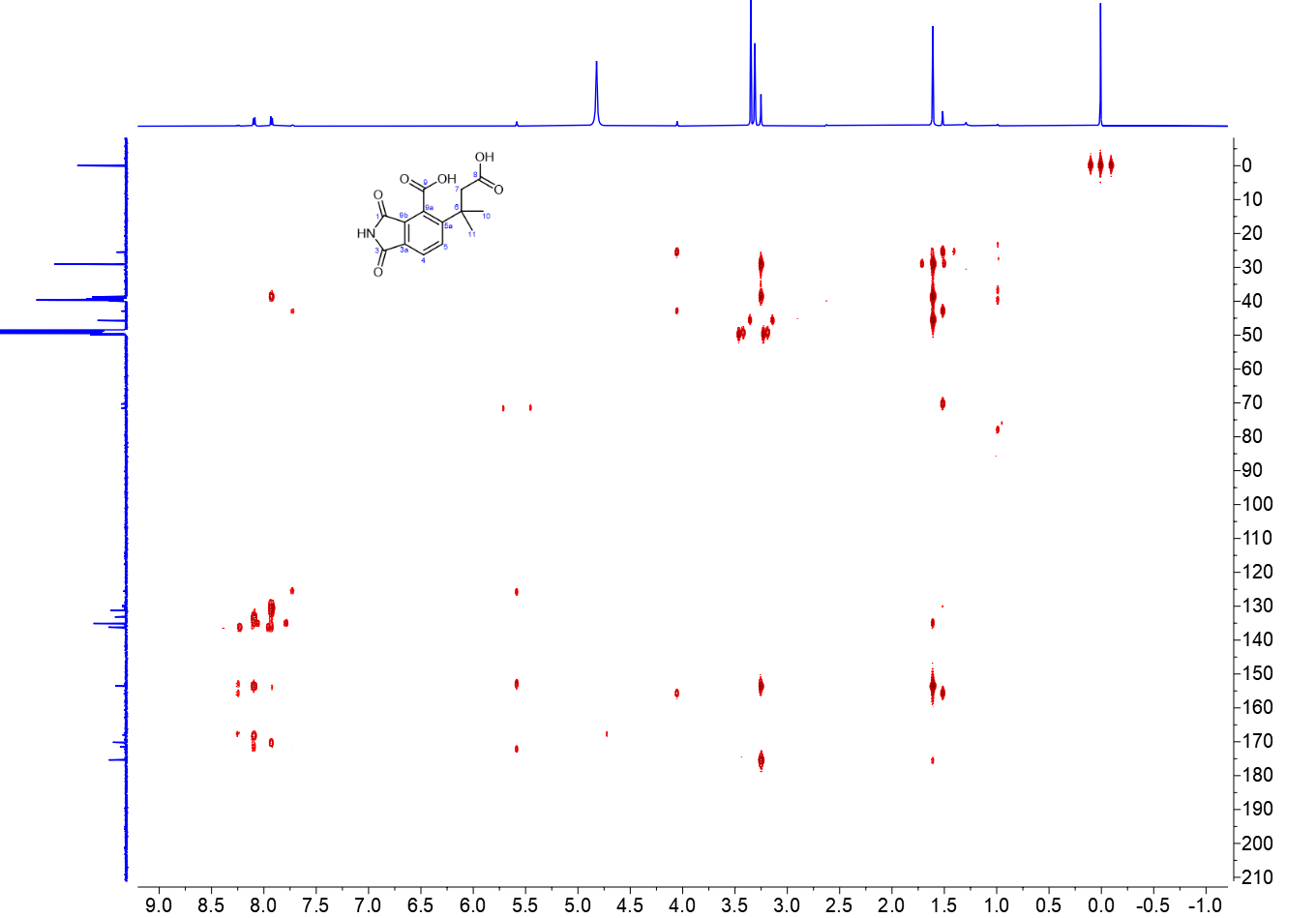
Figure S36. HMBC spectrum of **5** (CD_3_OD).


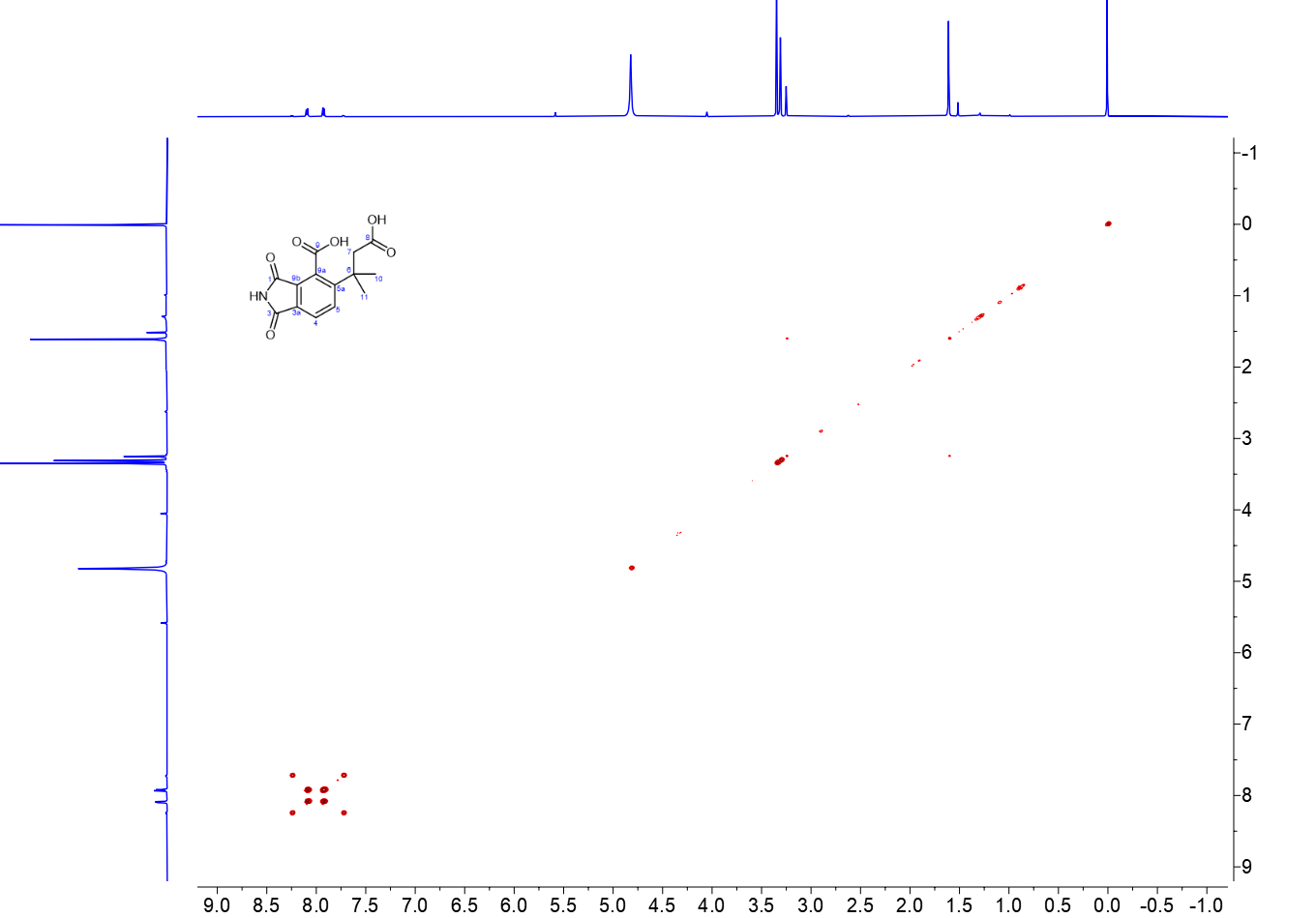
Figure S37. ^1^H-^1^H COSY spectrum of **5** (CD_3_OD).


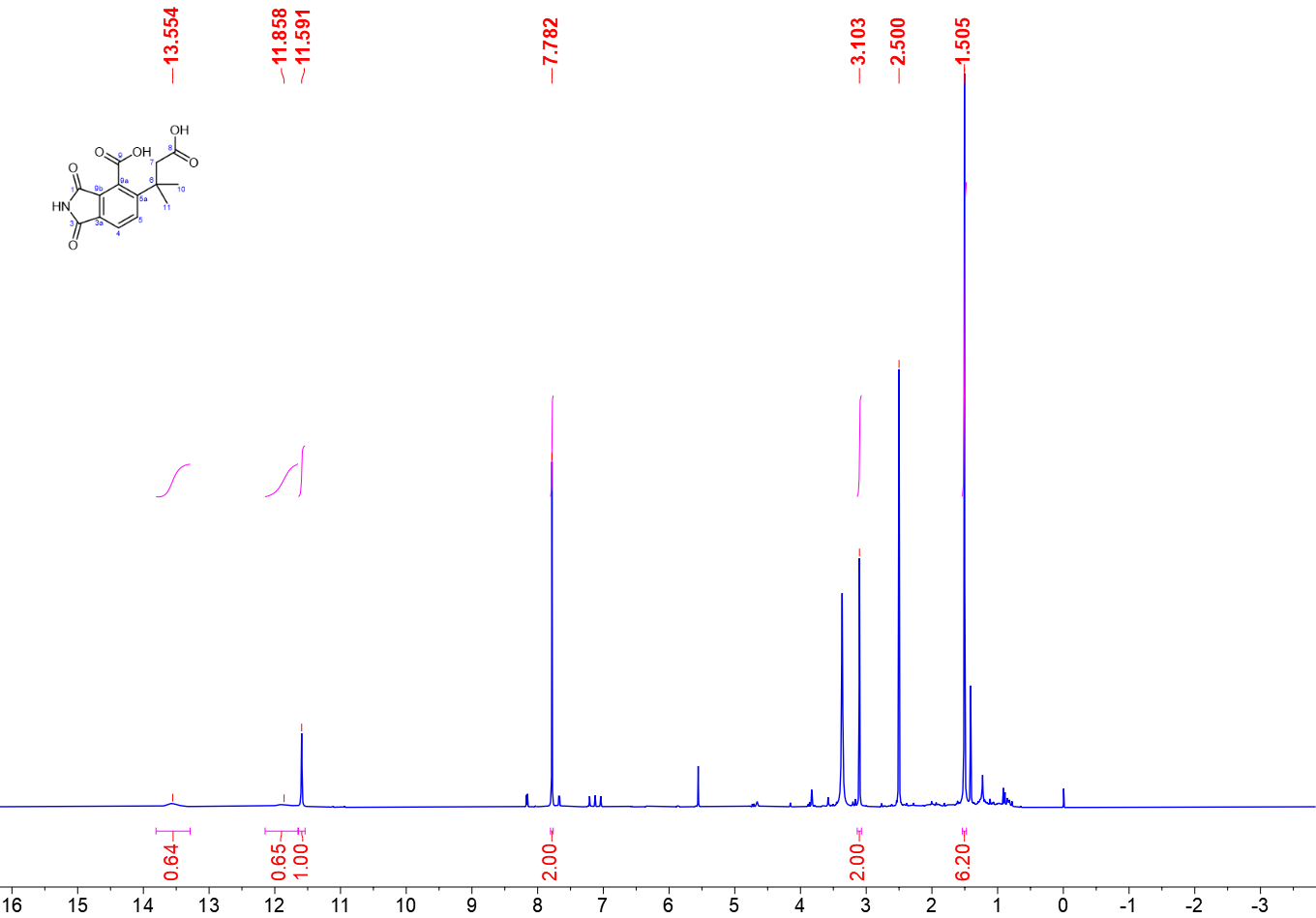


Figure S38. ^1^H NMR spectrum of **5** (DMSO-*d*_6_).


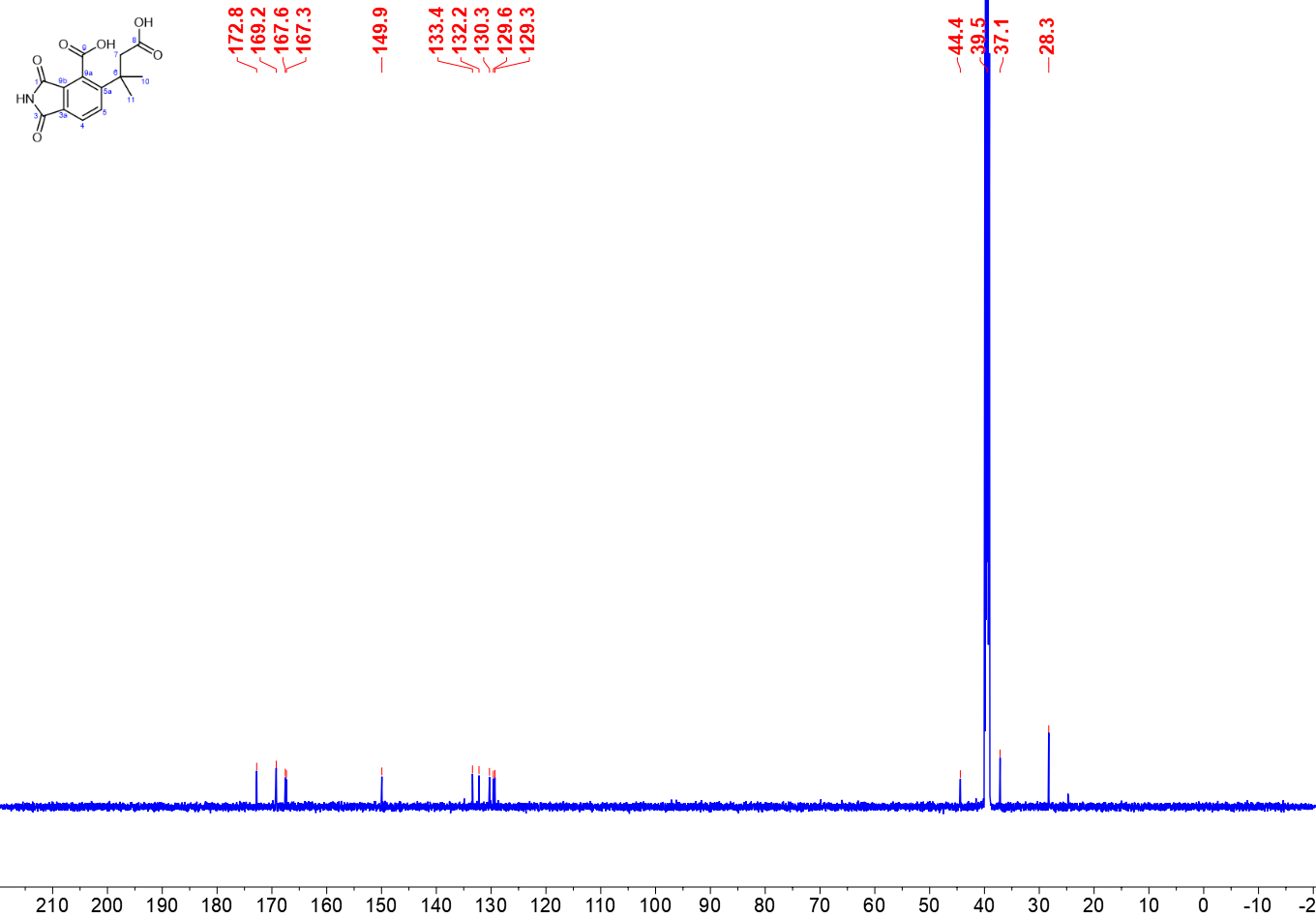
Figure S39. ^13^C NMR spectrum of **5** (DMSO-*d*_6_).


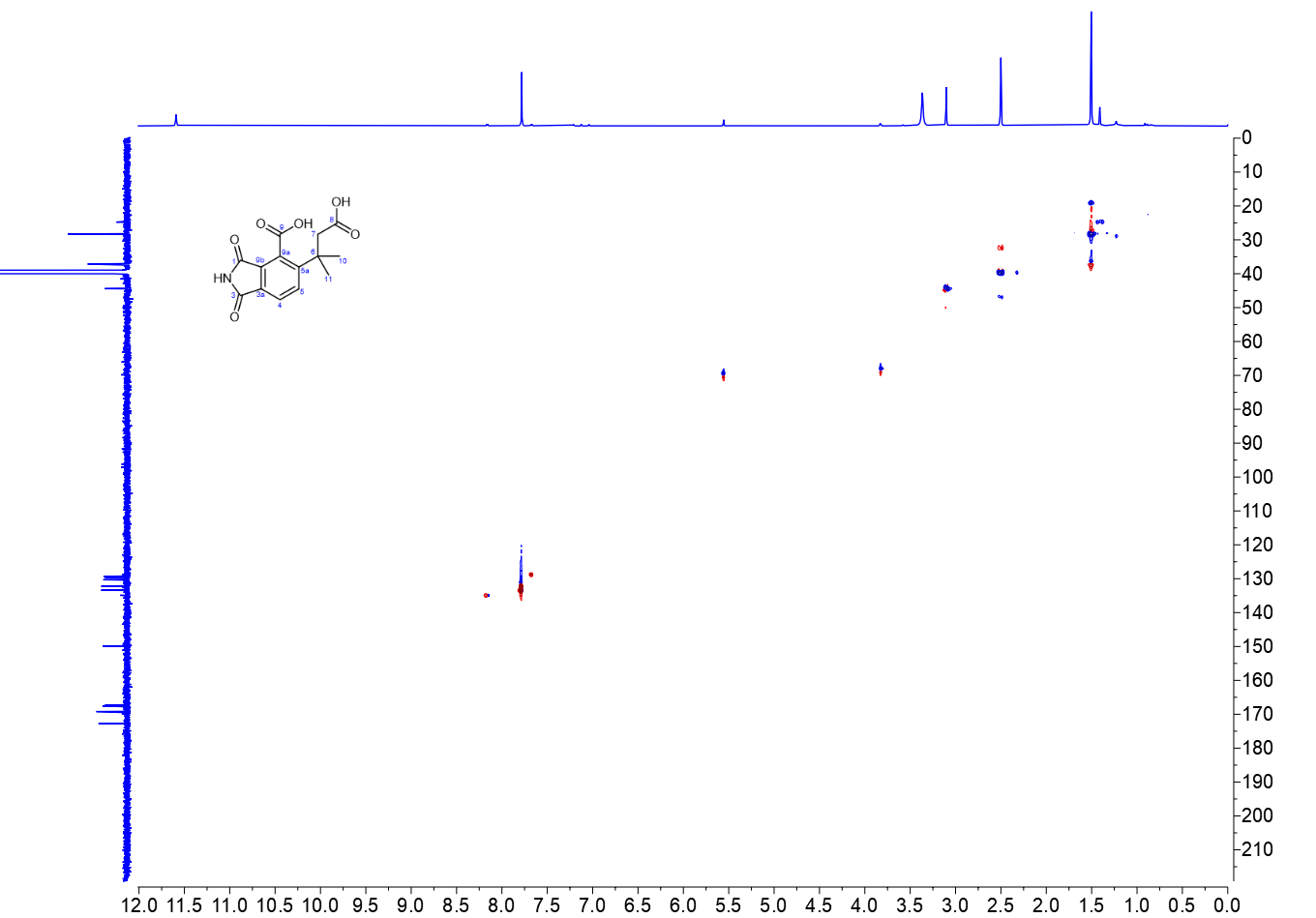
Figure S40. HSQC spectrum of **5** (DMSO-*d*_6_).


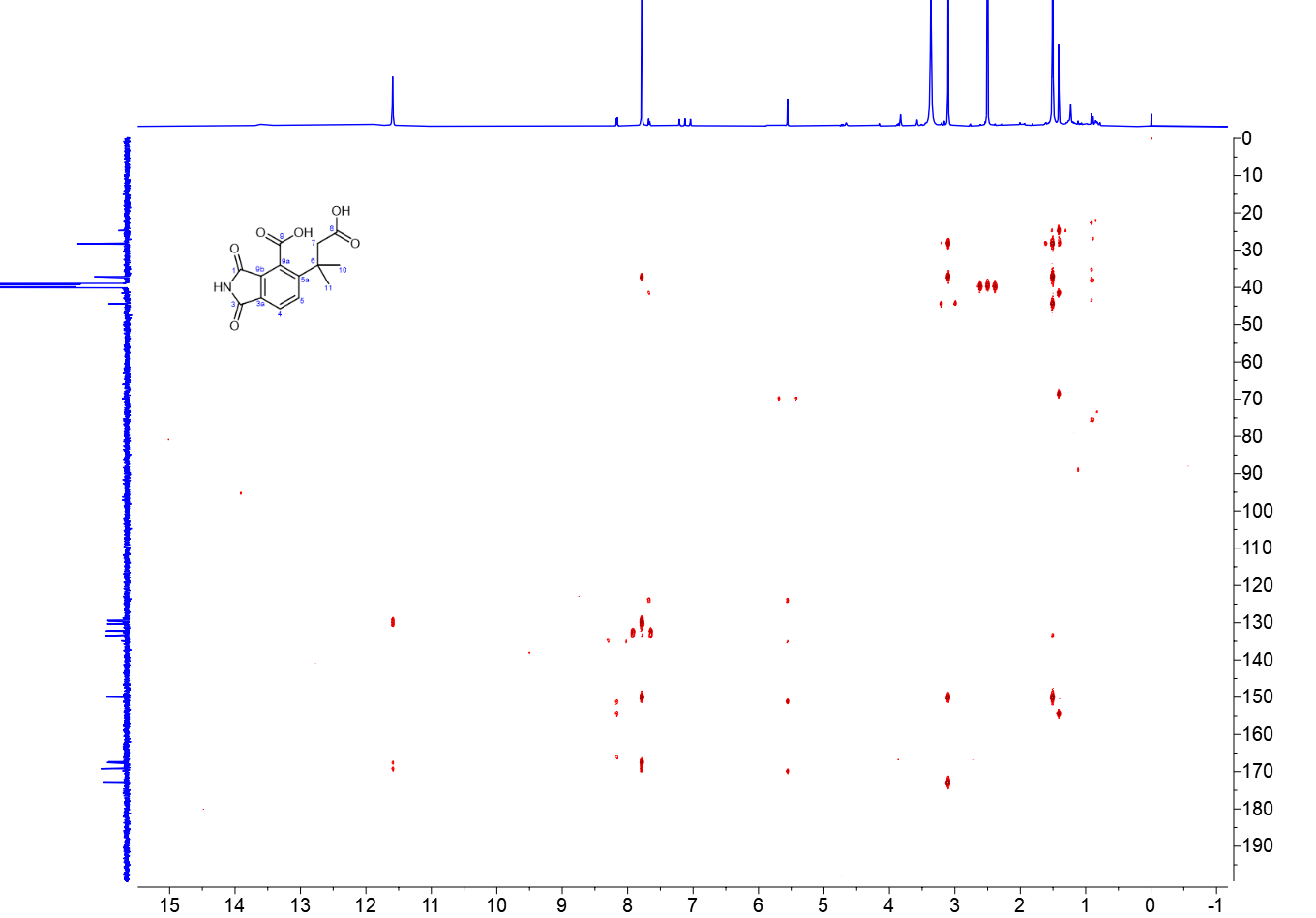
Figure S41. HMBC spectrum of **5** (DMSO-*d*_6_).


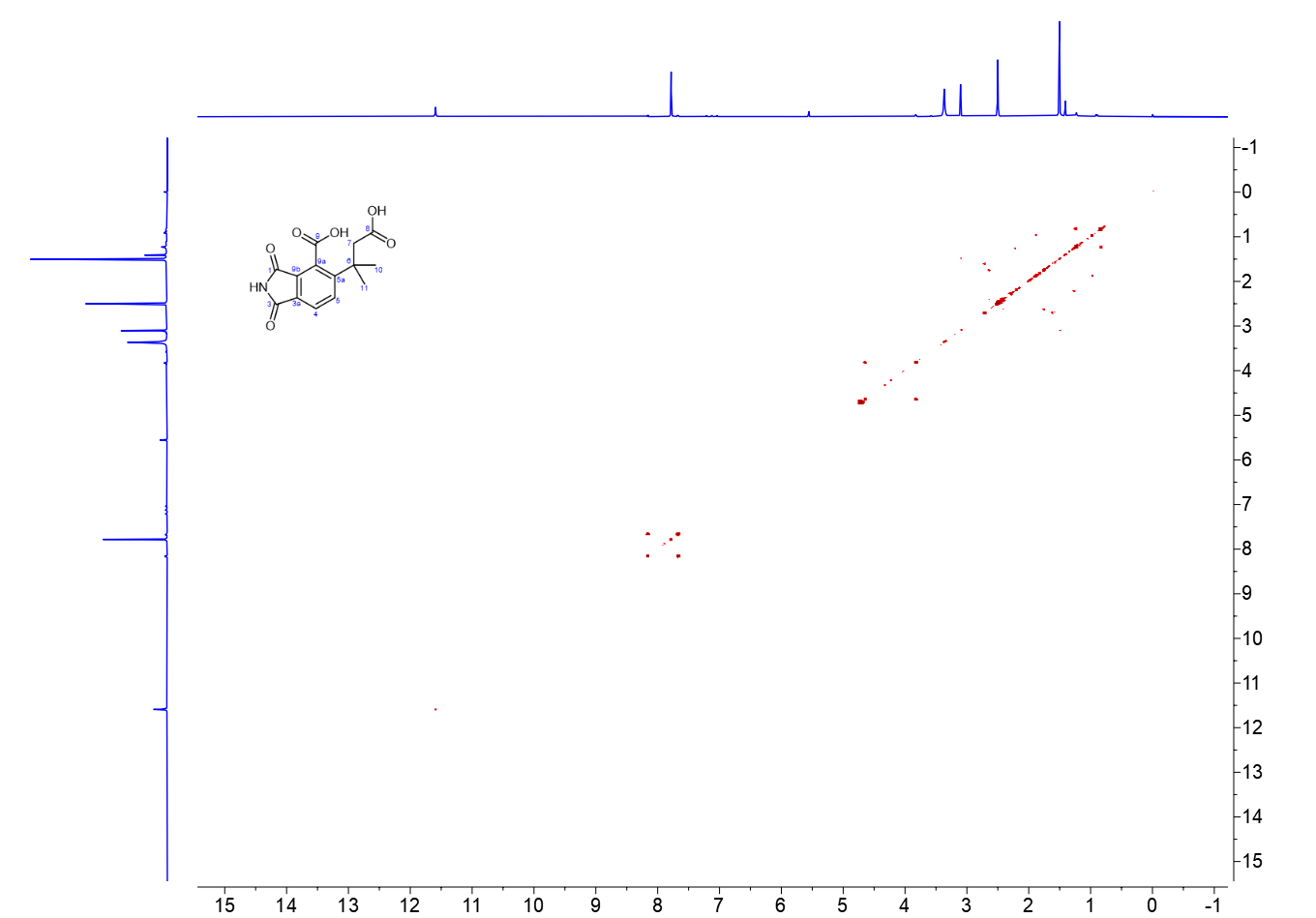
Figure S42. ^1^H-^1^H COSY spectrum of **5** (DMSO-*d*_6_).


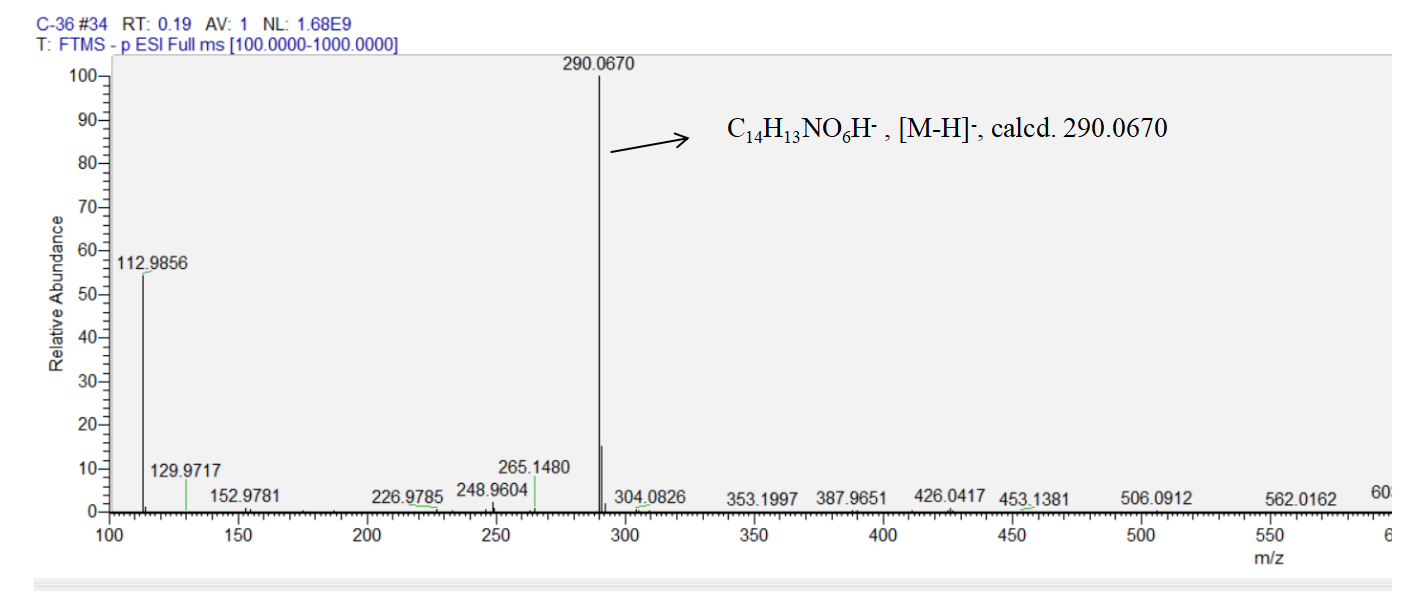
Figure S43. HRESIMS spectrum of **5.**





Figure S44. The UV spectrum of compound **5.**


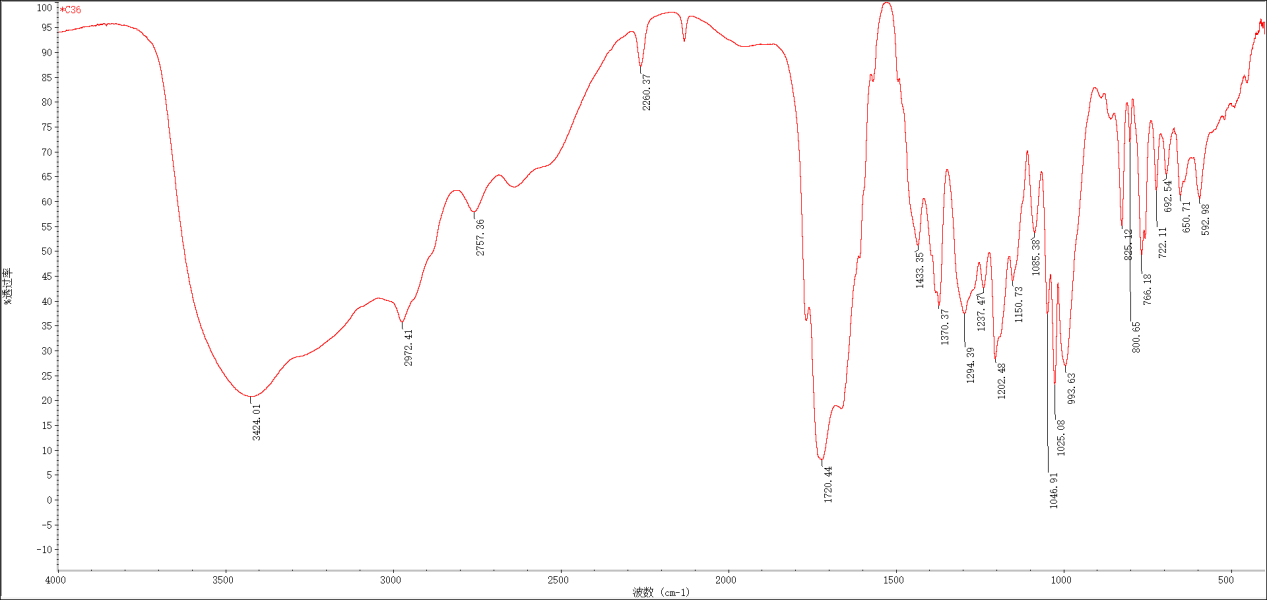
Figure S45. The IR spectrum of compound **5.**


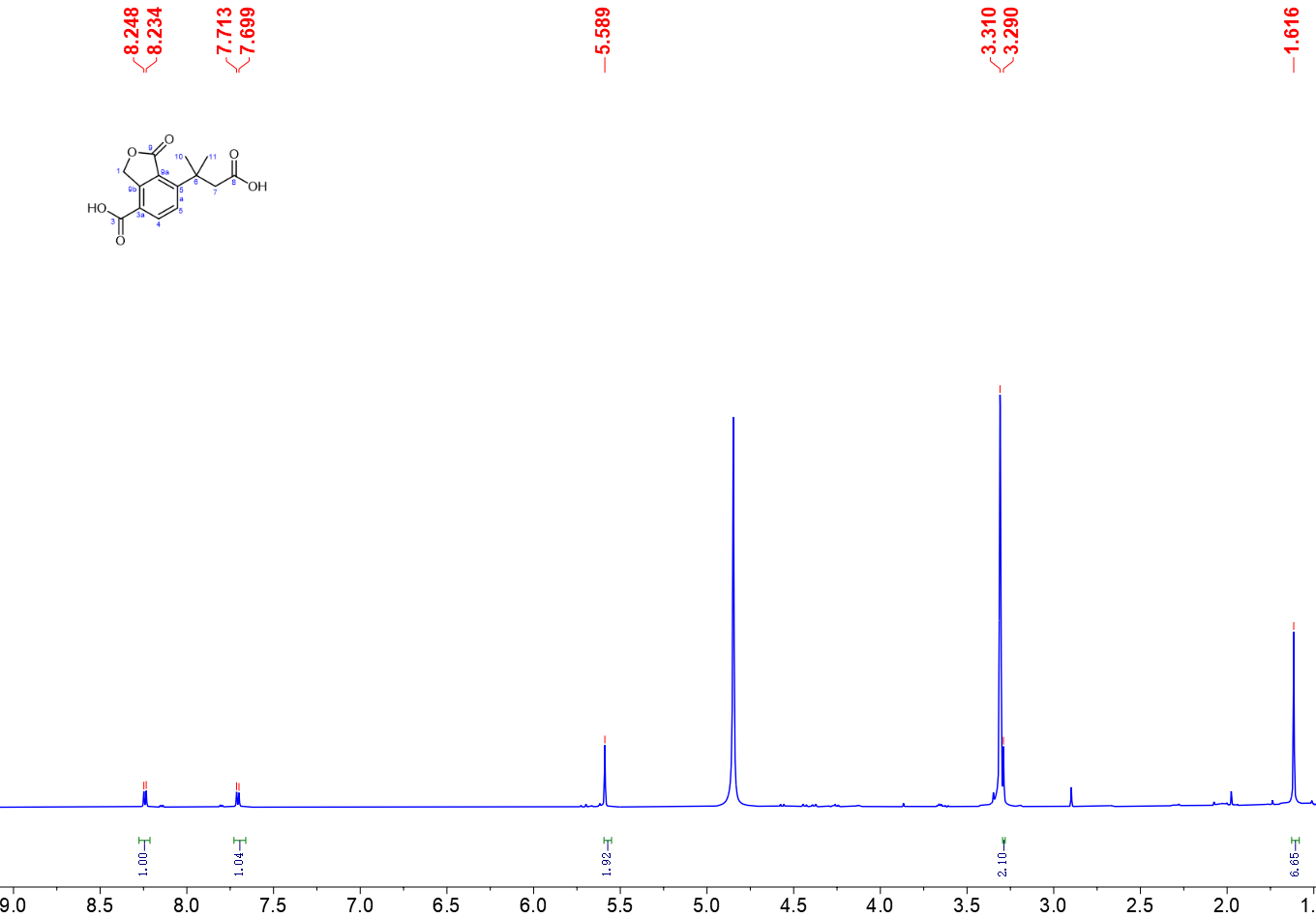


Figure S46. ^1^H NMR spectrum of **6**.


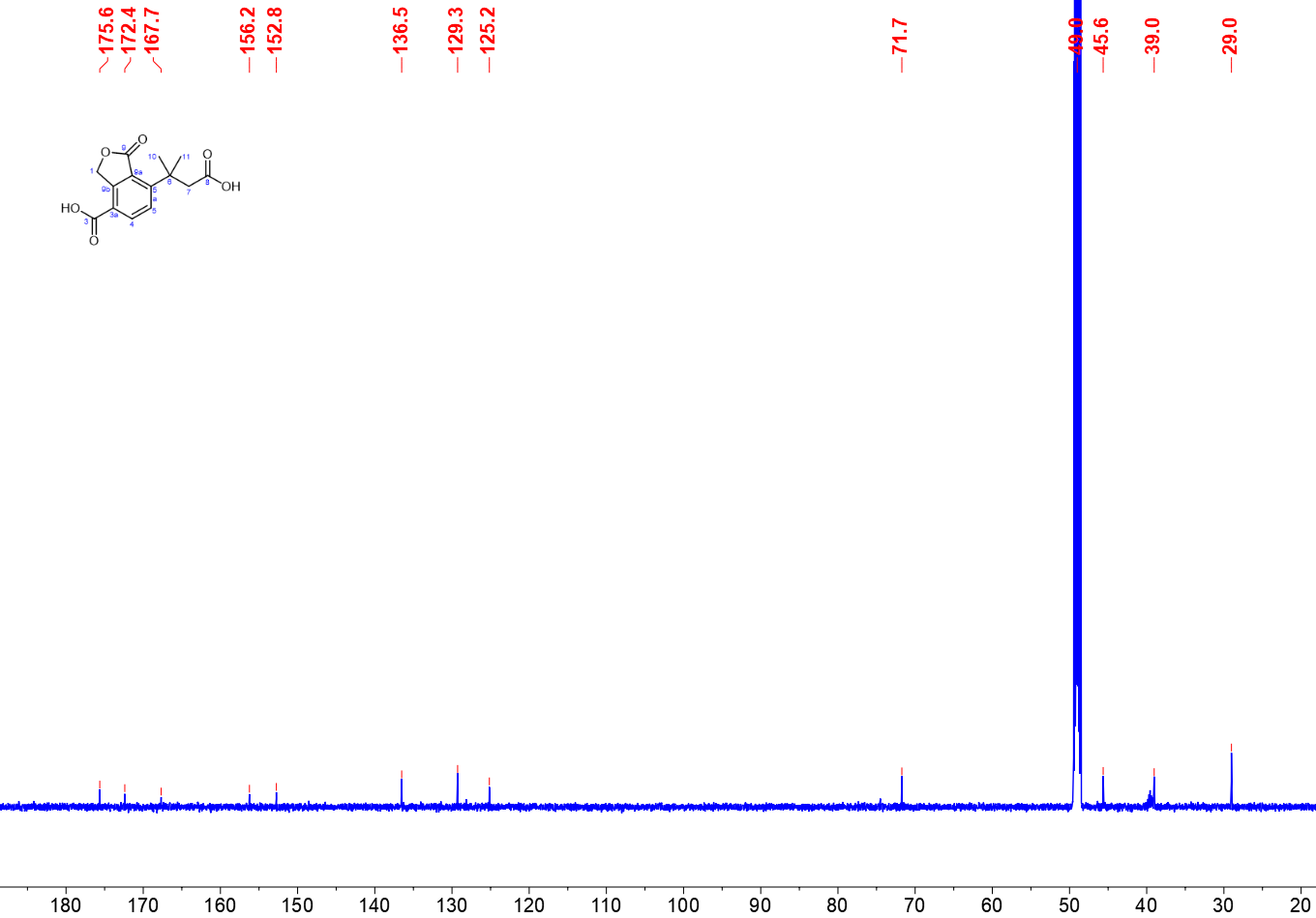
Figure S47. ^13^C NMR spectrum of **6.**


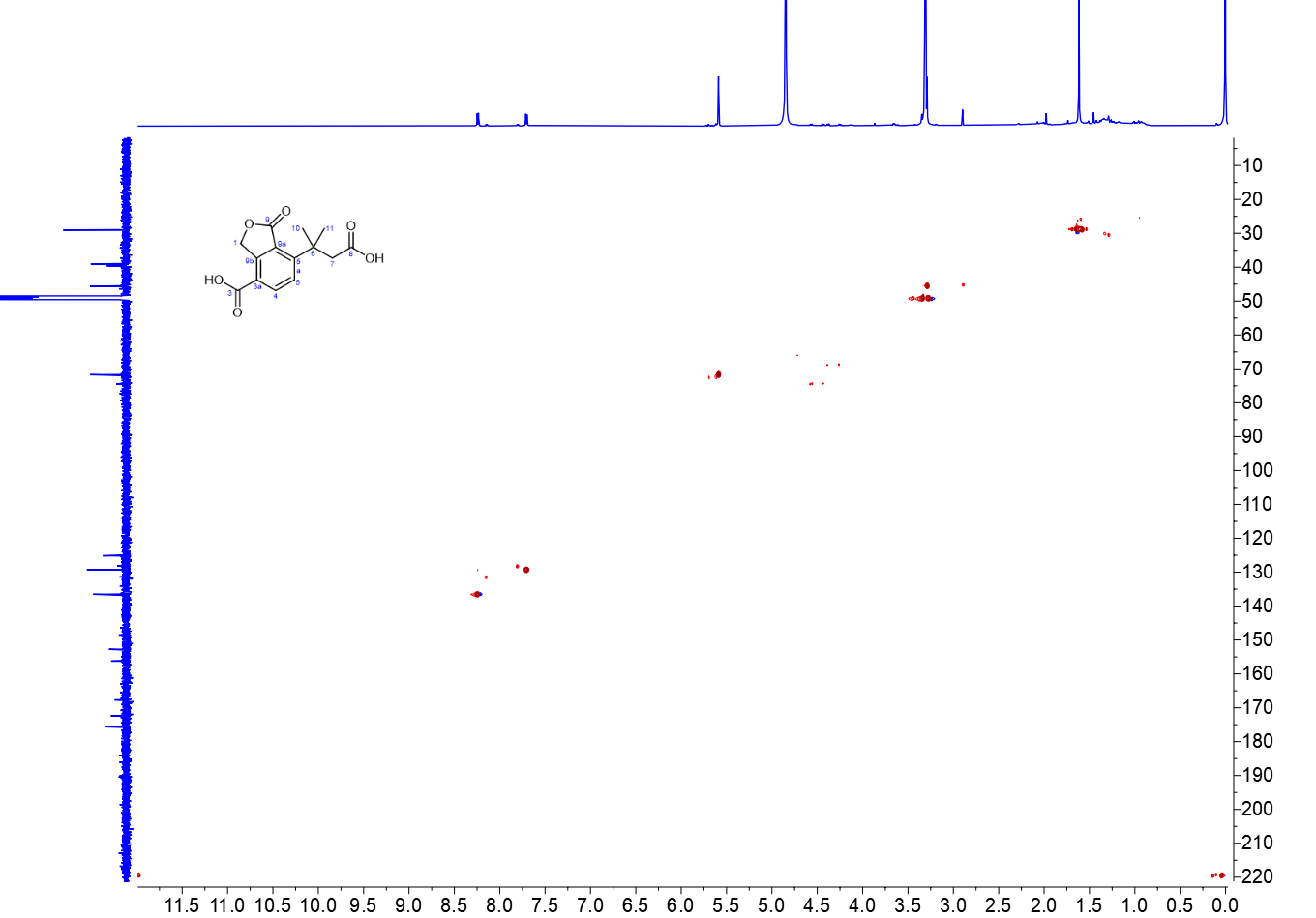
Figure S48. HSQC spectrum of **6.**


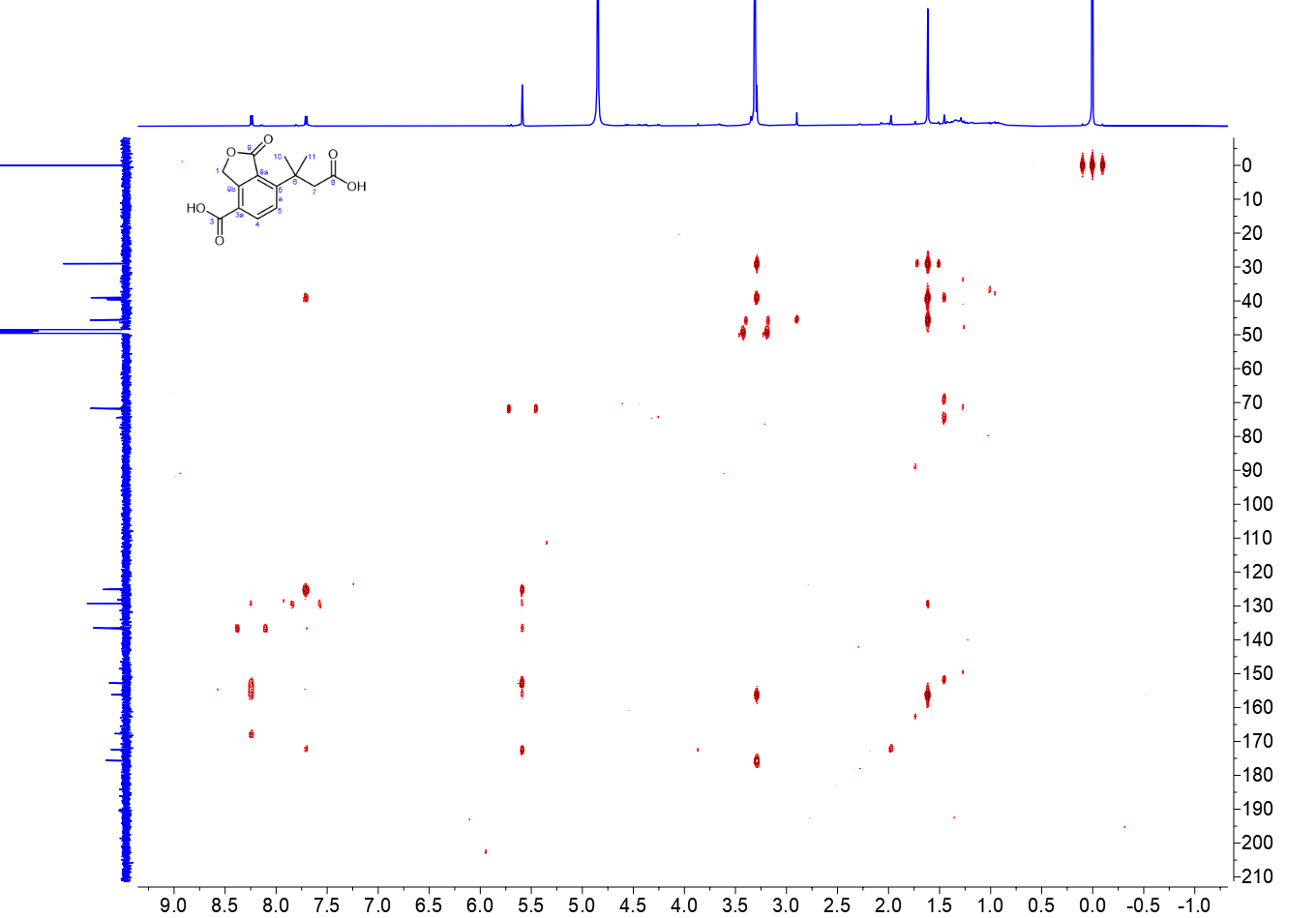
Figure S49. HMBC spectrum of **6.**


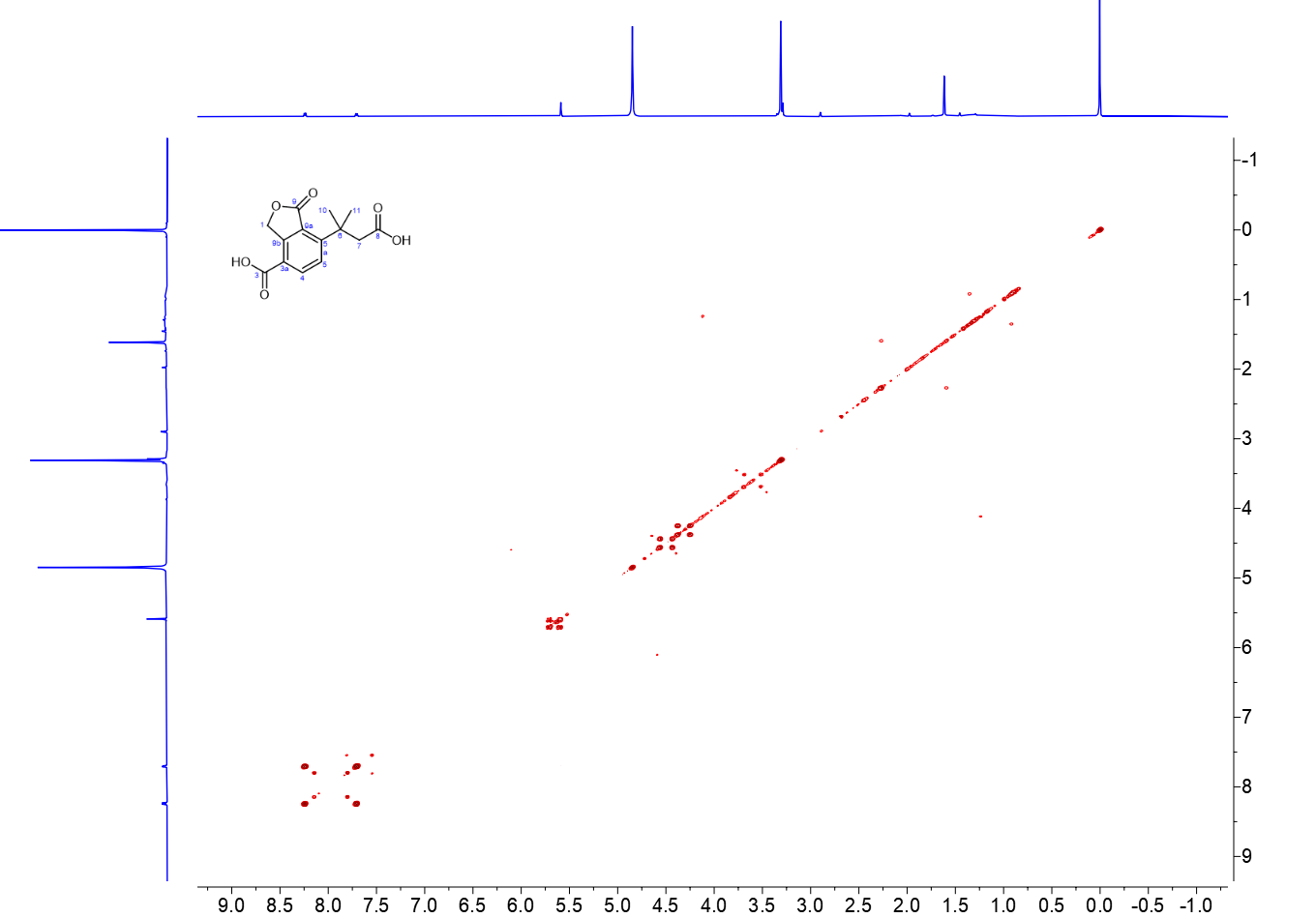
Figure S50. ^1^H-^1^H COSY spectrum of **6.**


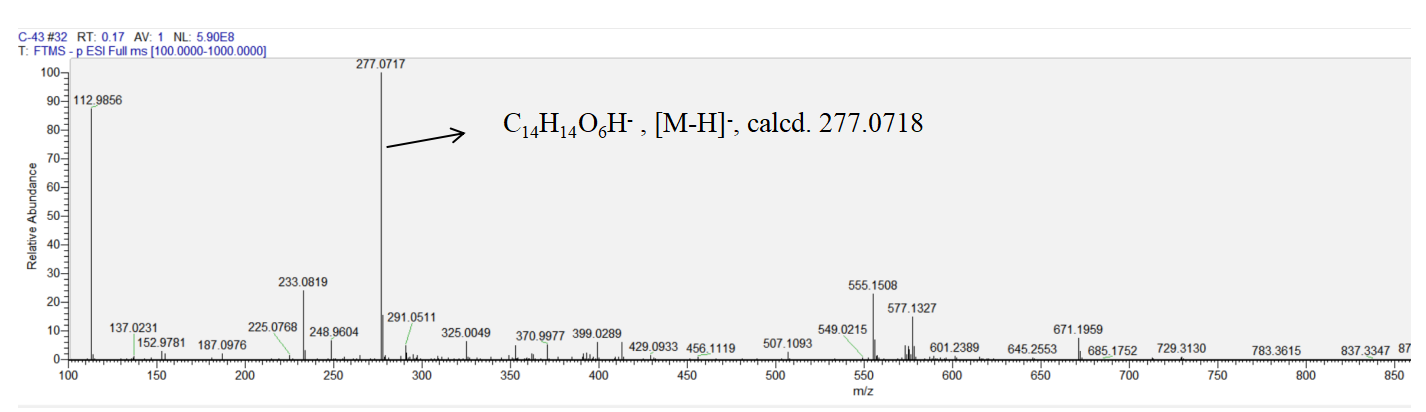
Figure S51. HRESIMS spectrum of **6.**





Figure S52. The UV spectrum of compound **6.**


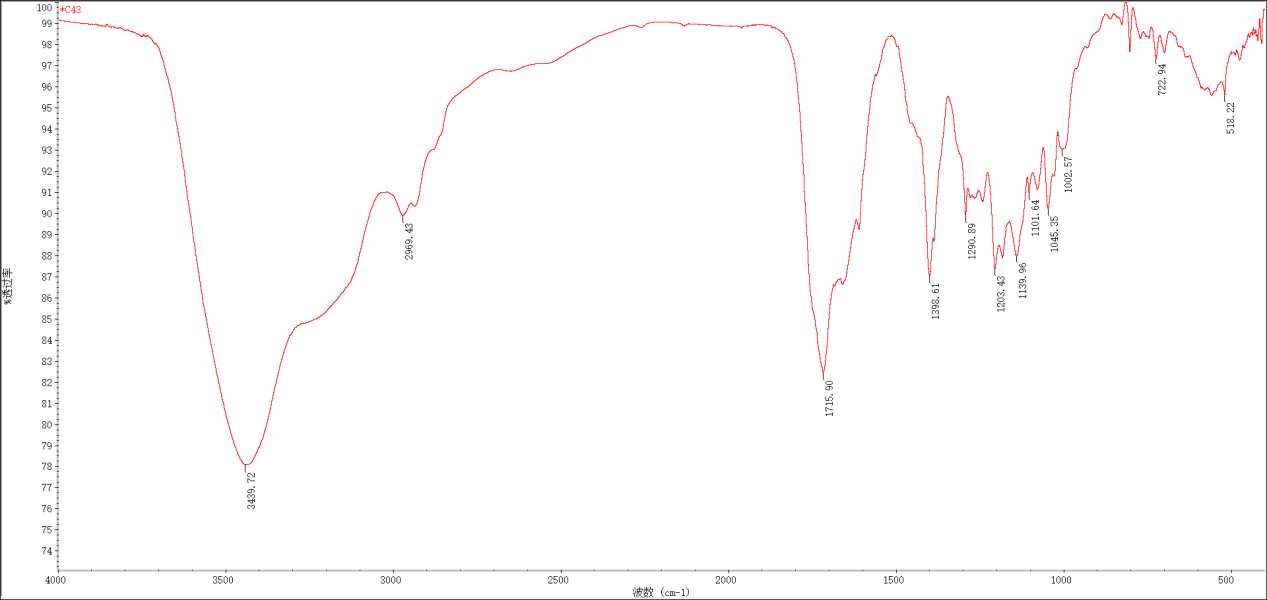
Figure S53. The IR spectrum of compound **6.**


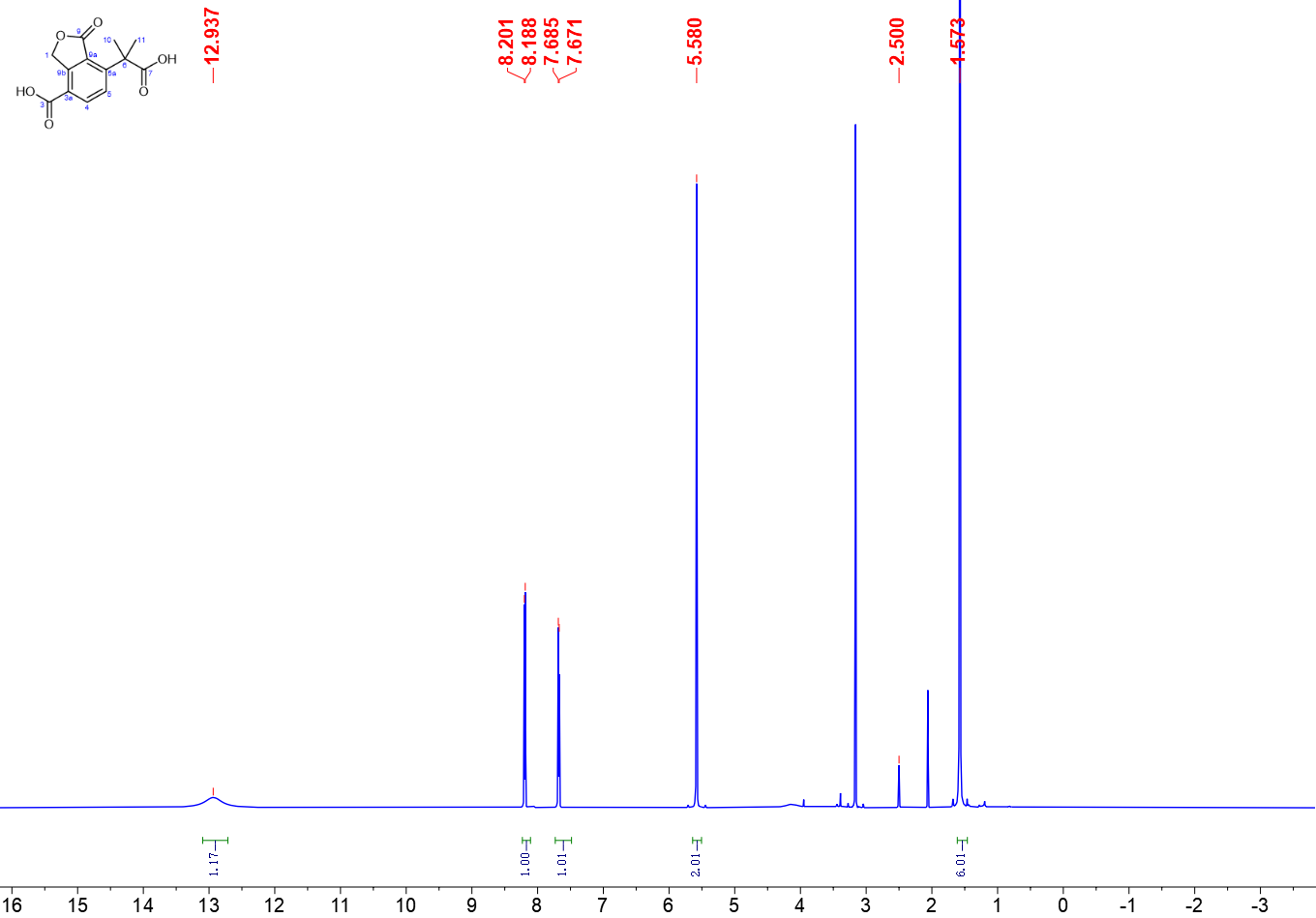
Figure S54. ^1^H NMR spectrum of **7.**


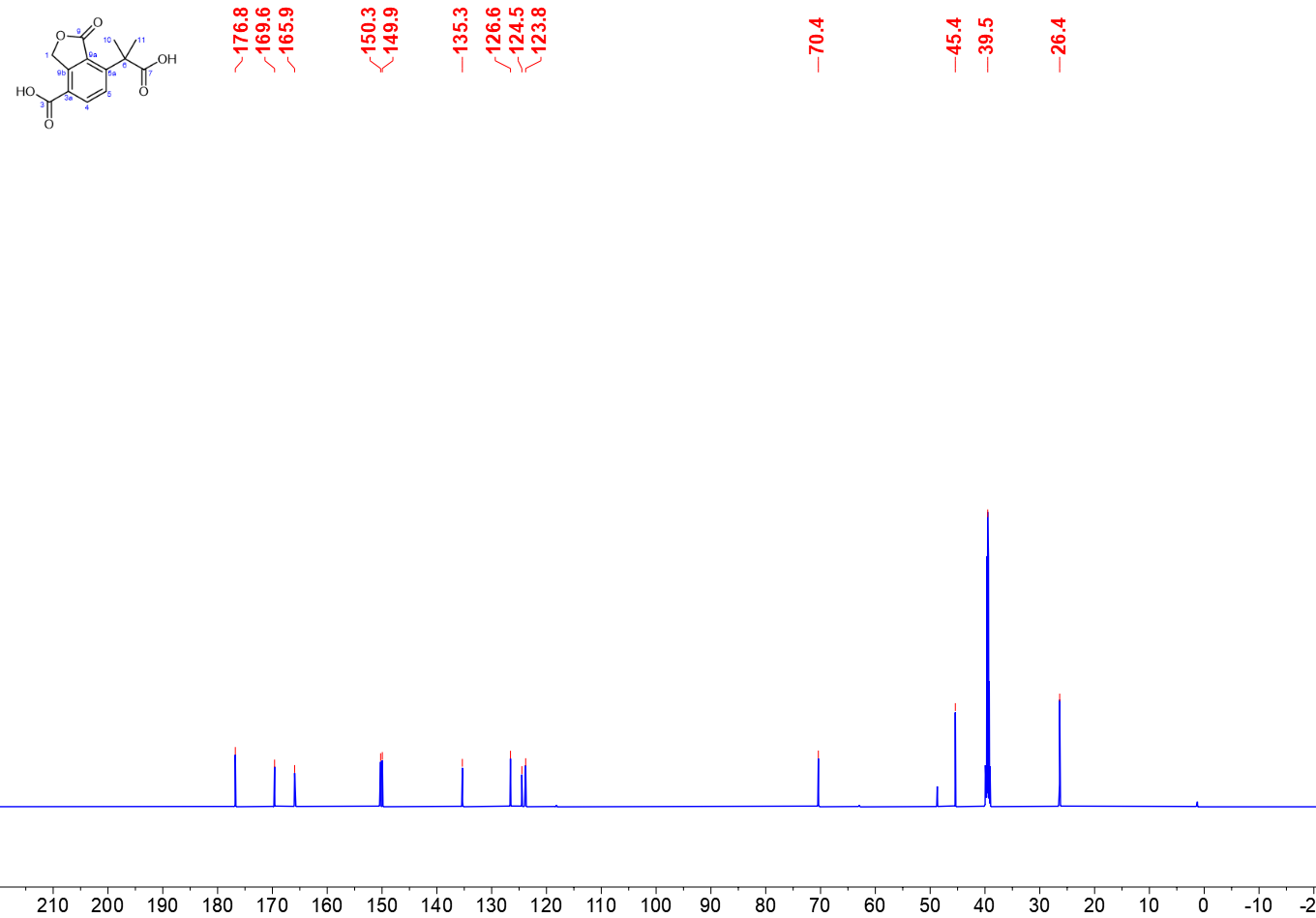
Figure S55. ^13^C NMR spectrum of **7.**


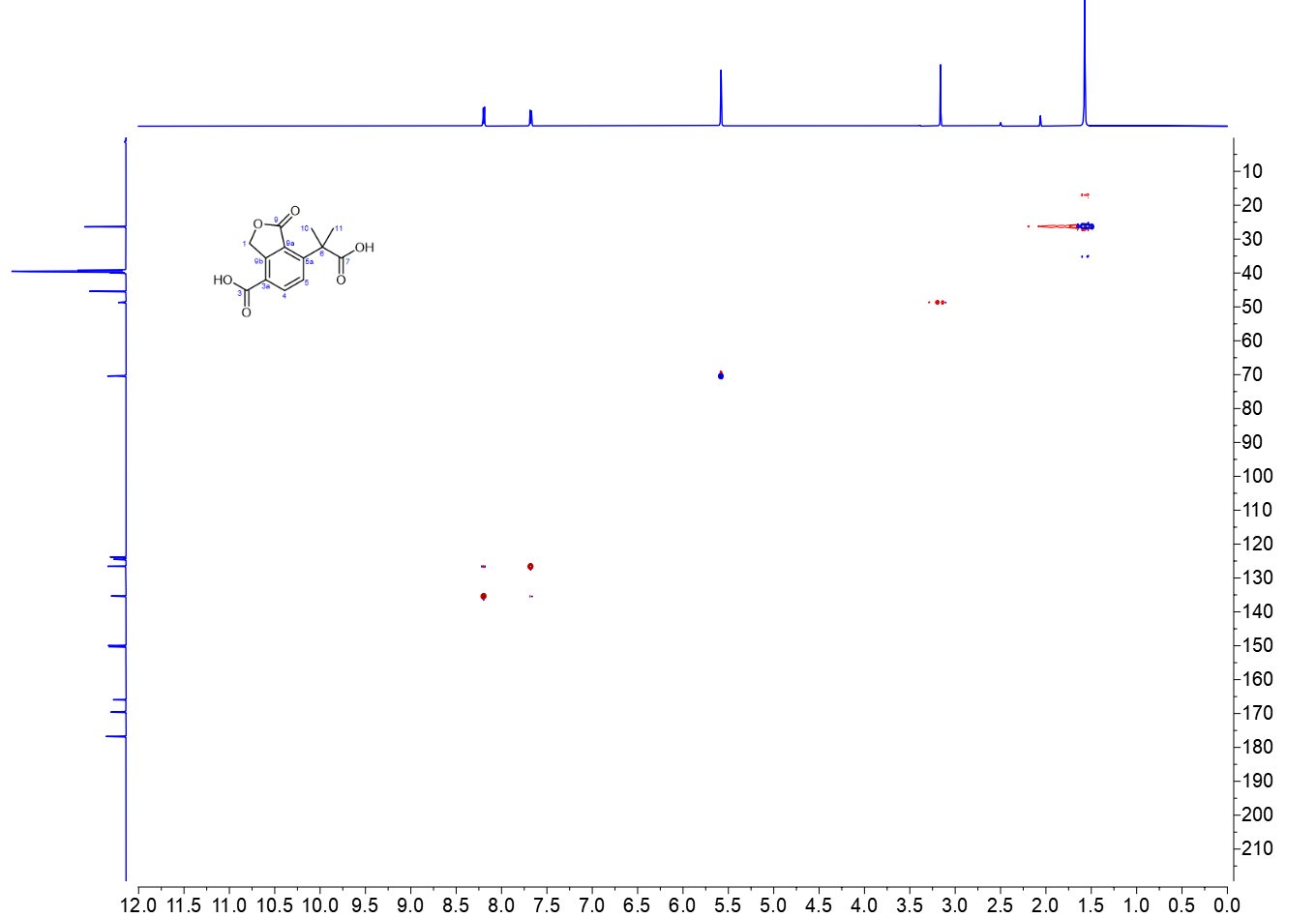
Figure S56. HSQC spectrum of **7.**


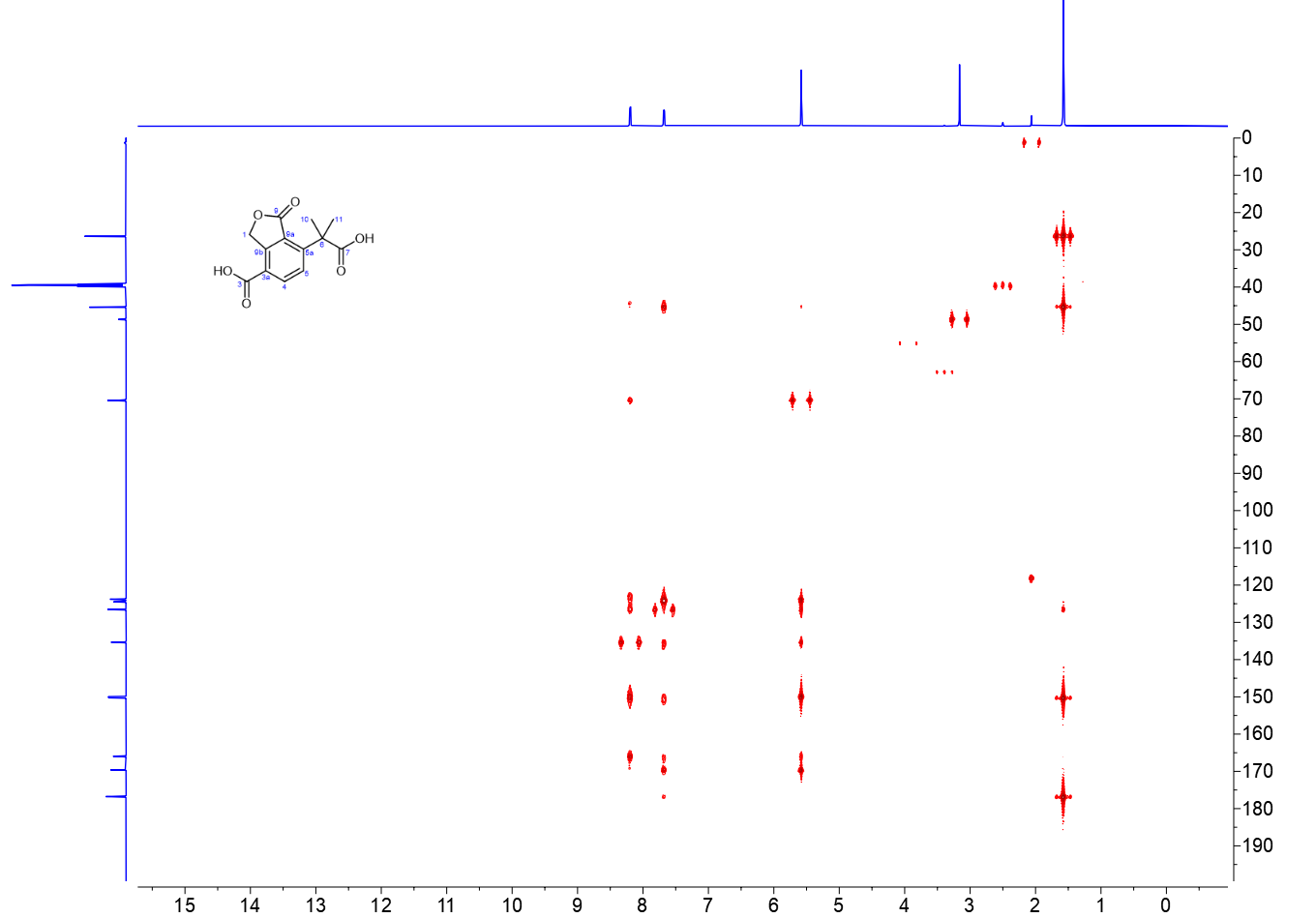
Figure S57. HMBC spectrum of **7.**


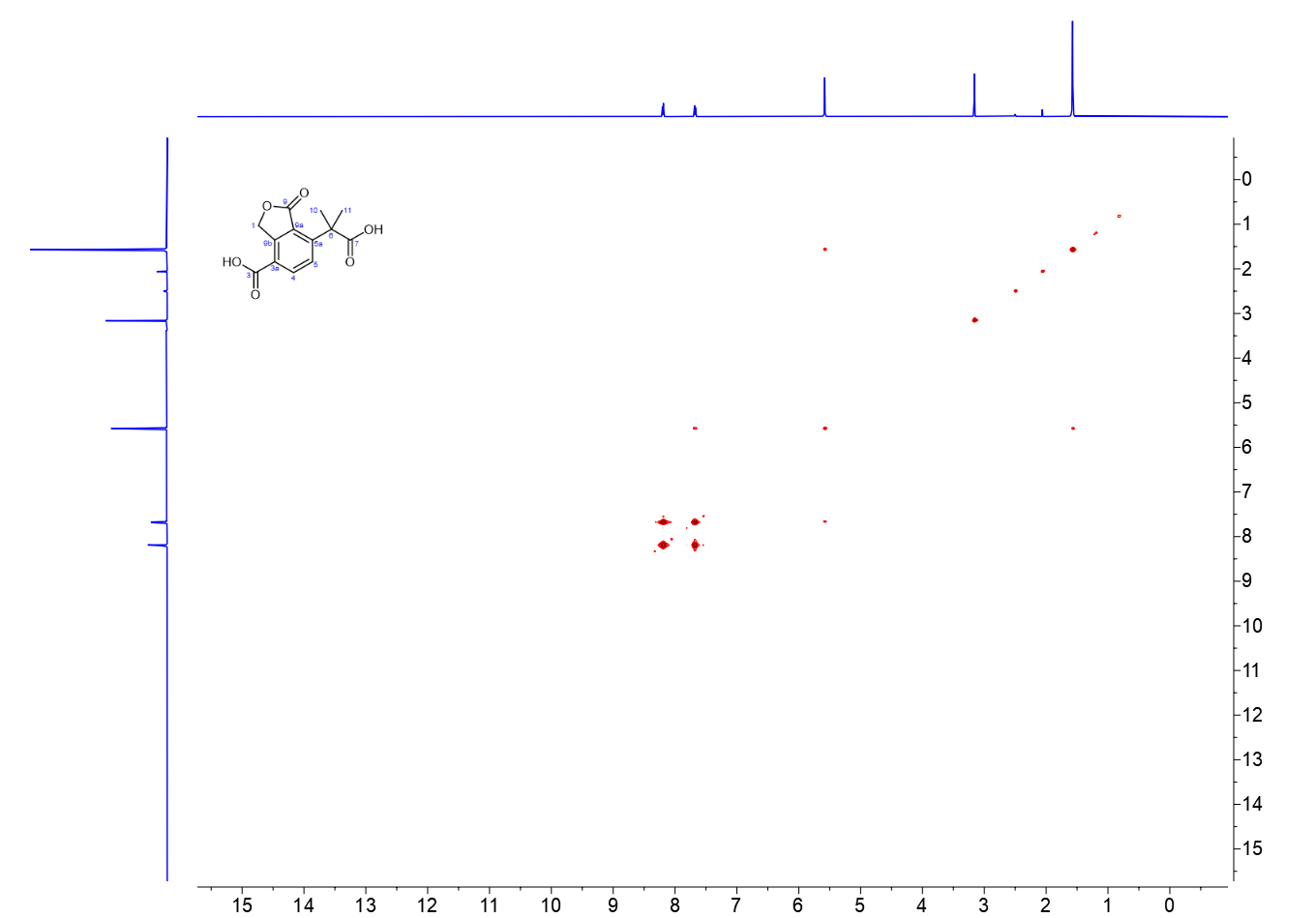
Figure S58. ^1^H-^1^H COSY spectrum of **7.**


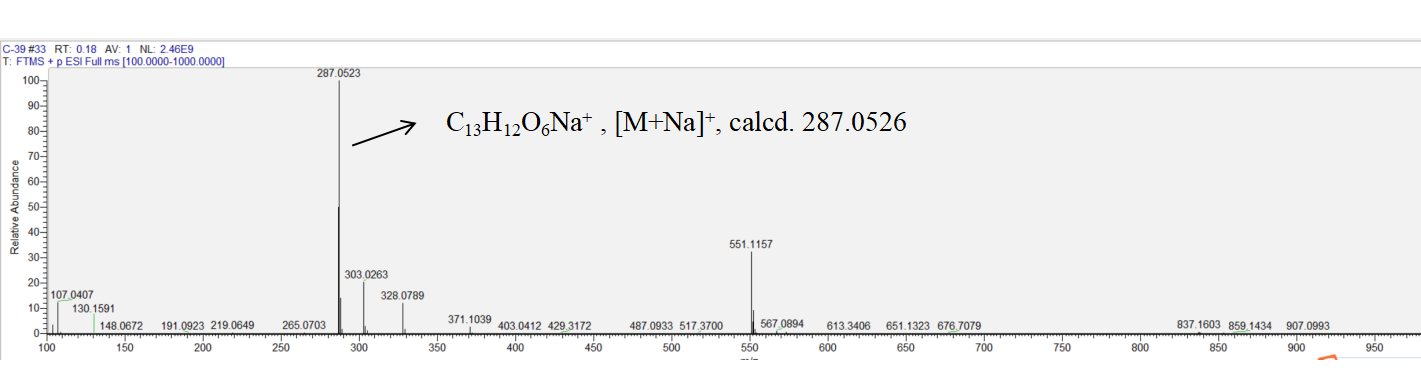
Figure S59. HRESIMS spectrum of **7.**





Figure S60. The UV spectrum of compound **7.**


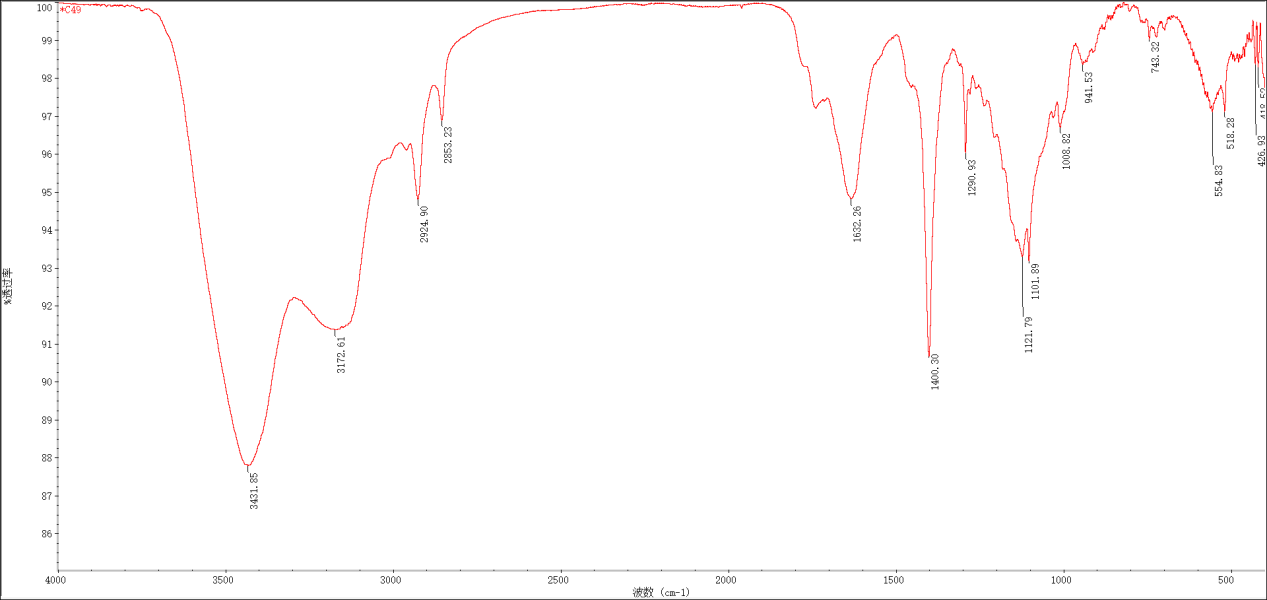
Figure S61. The IR spectrum of compound **7.**


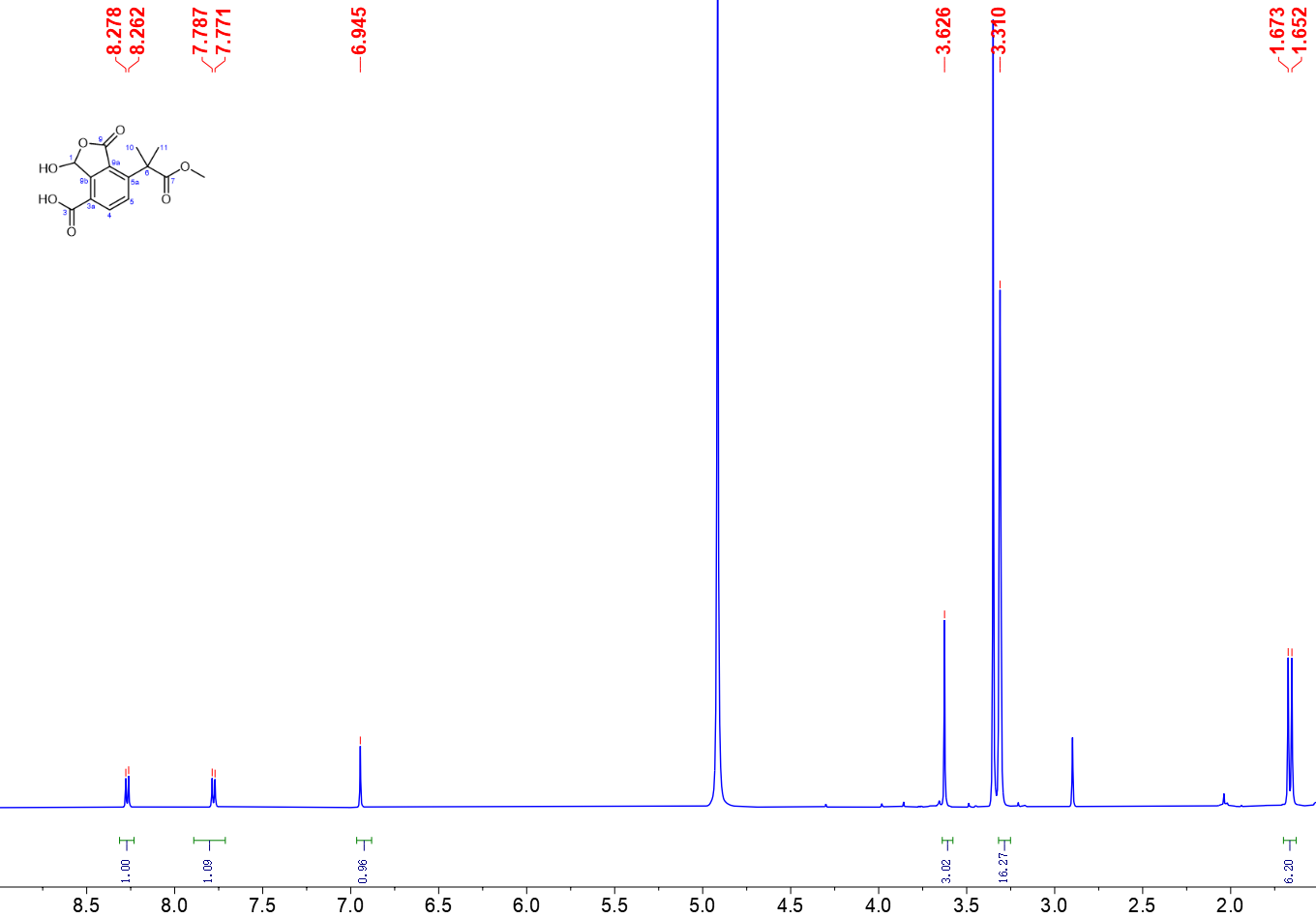
Figure S62. ^1^H NMR spectrum of **8.**


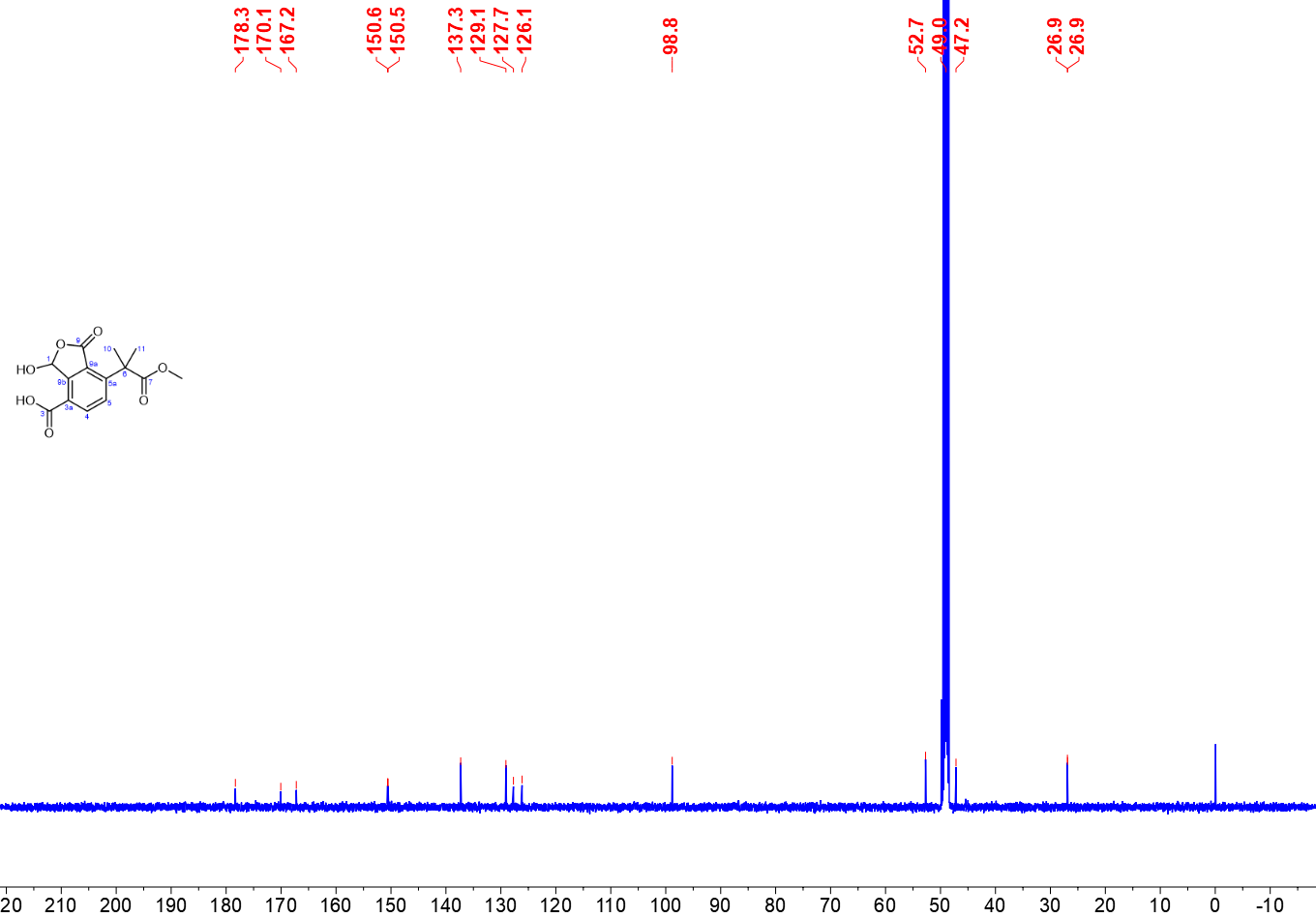
Figure S63. ^13^C NMR spectrum of **8.**


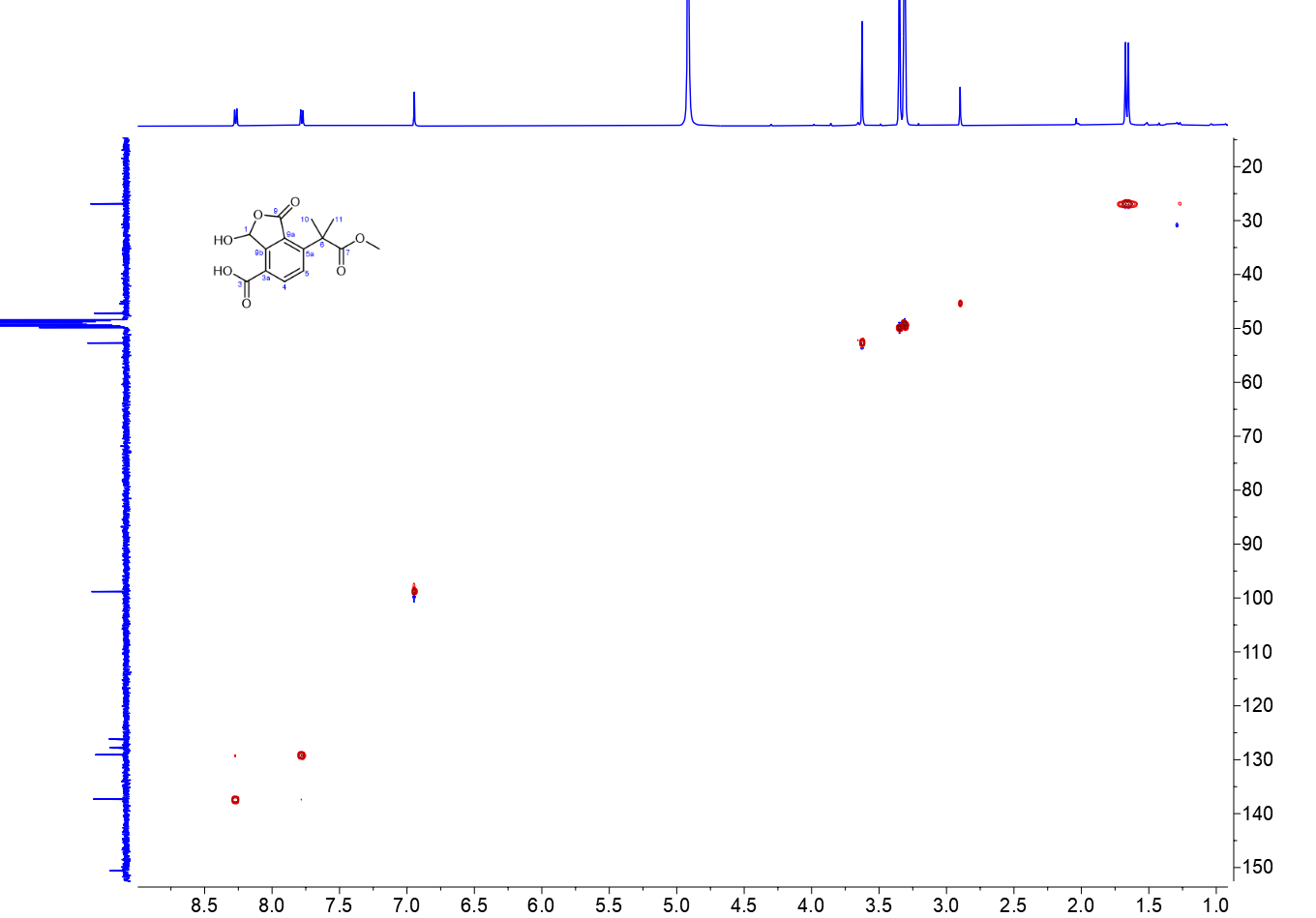
Figure S64. HSQC spectrum of **8.**


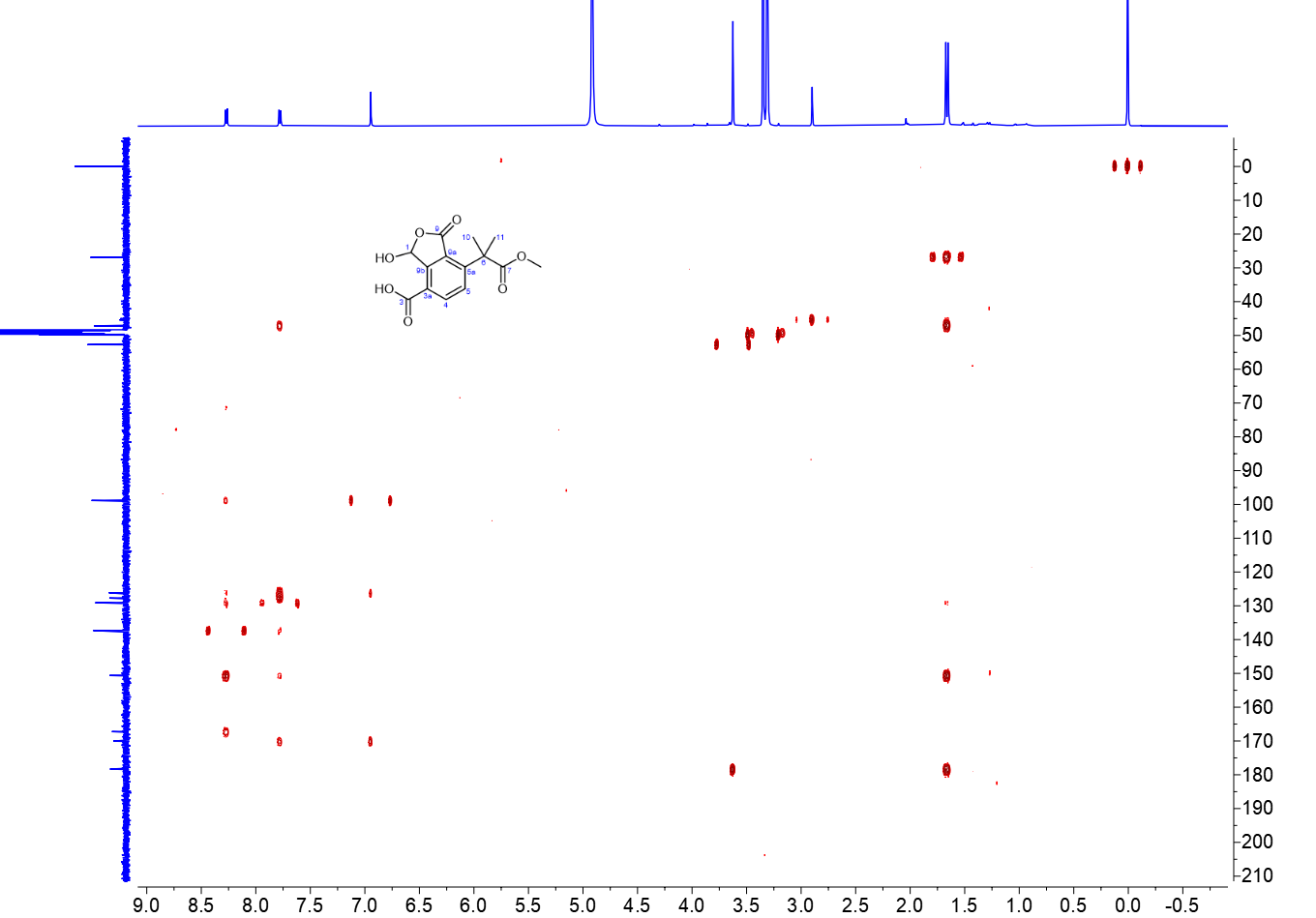
Figure S65. HMBC spectrum of **8.**


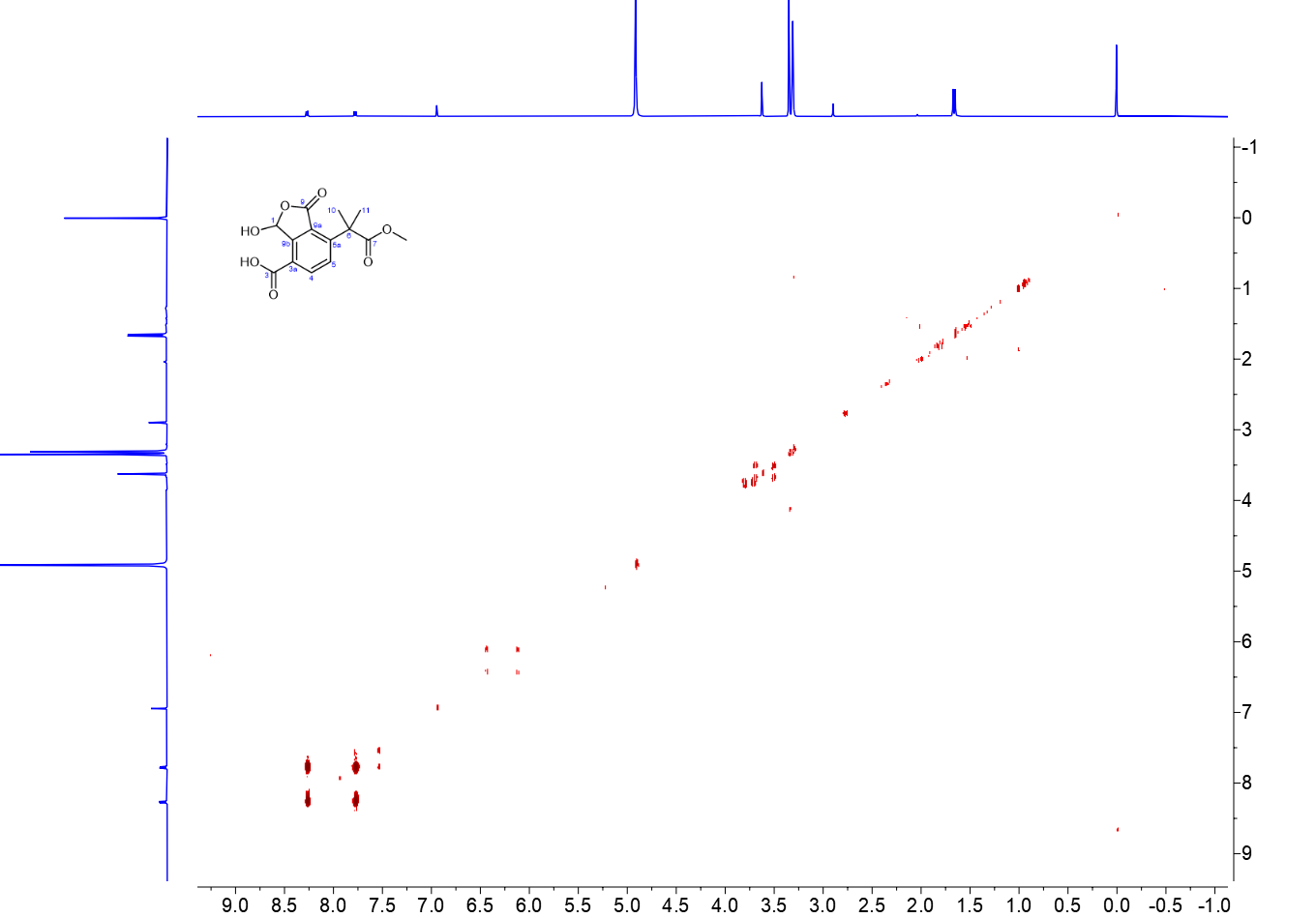
Figure S66. ^1^H-^1^H COSY spectrum of **8.**


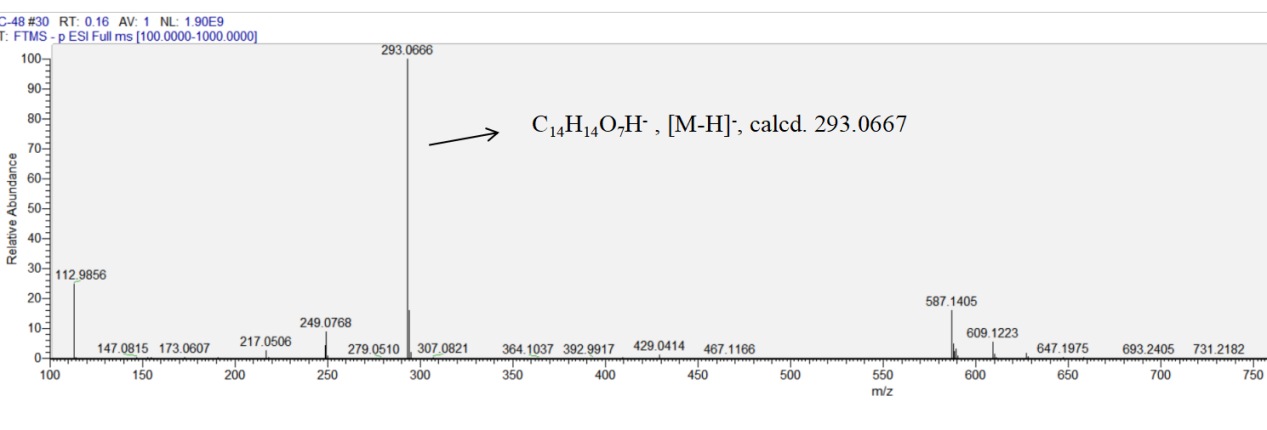
Figure S67. HRESIMS spectrum of **8.**





Figure S68. The UV spectrum of compound **8.**


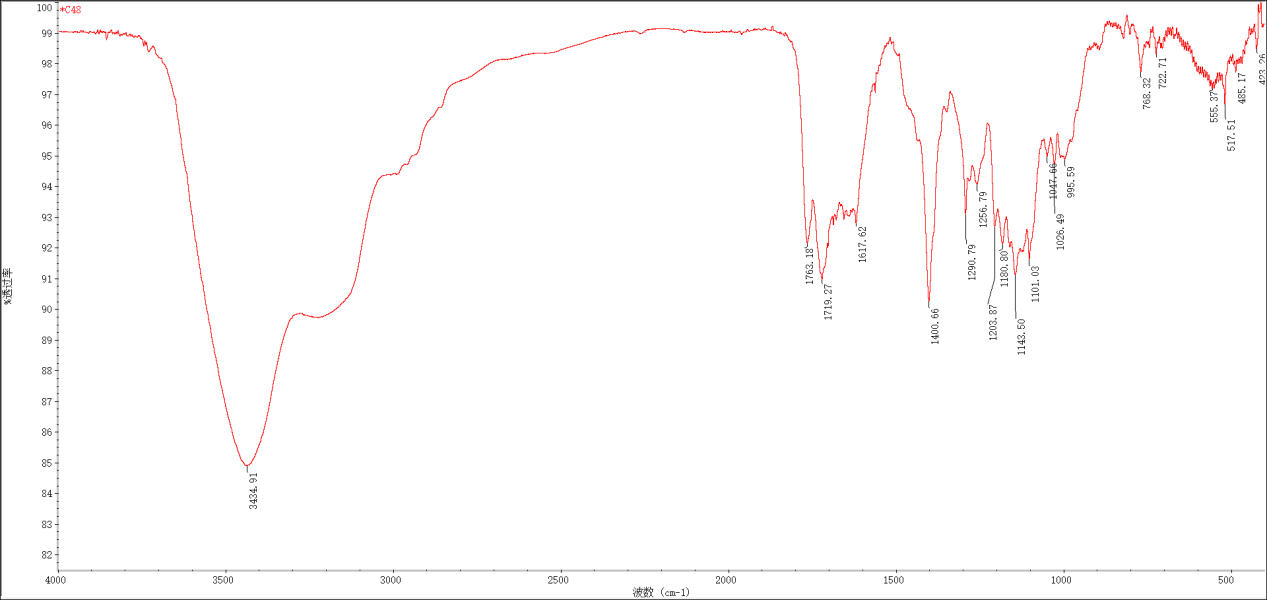
Figure S69. The IR spectrum of compound **8.**


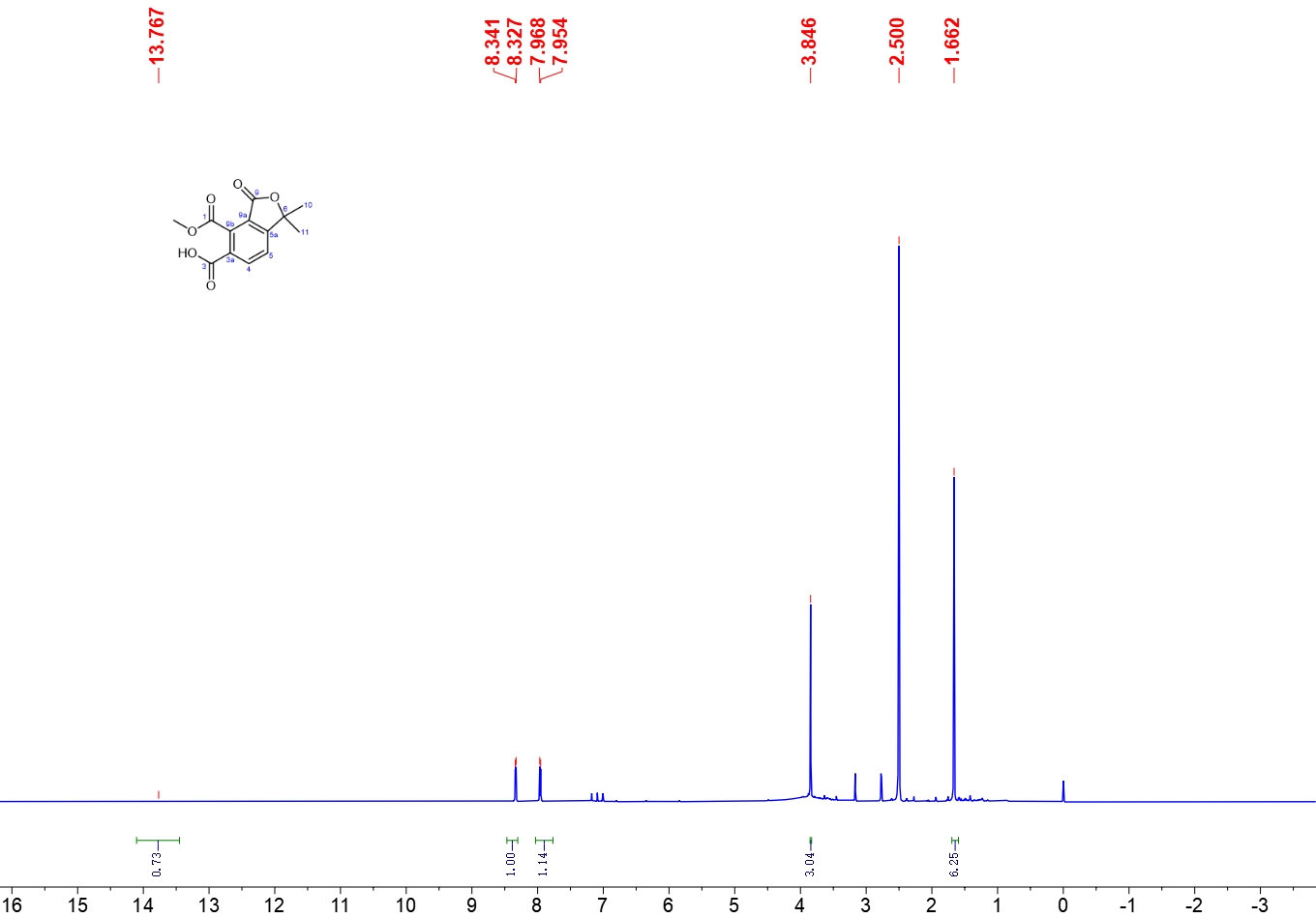
Figure S70. ^1^H NMR spectrum of **9.**


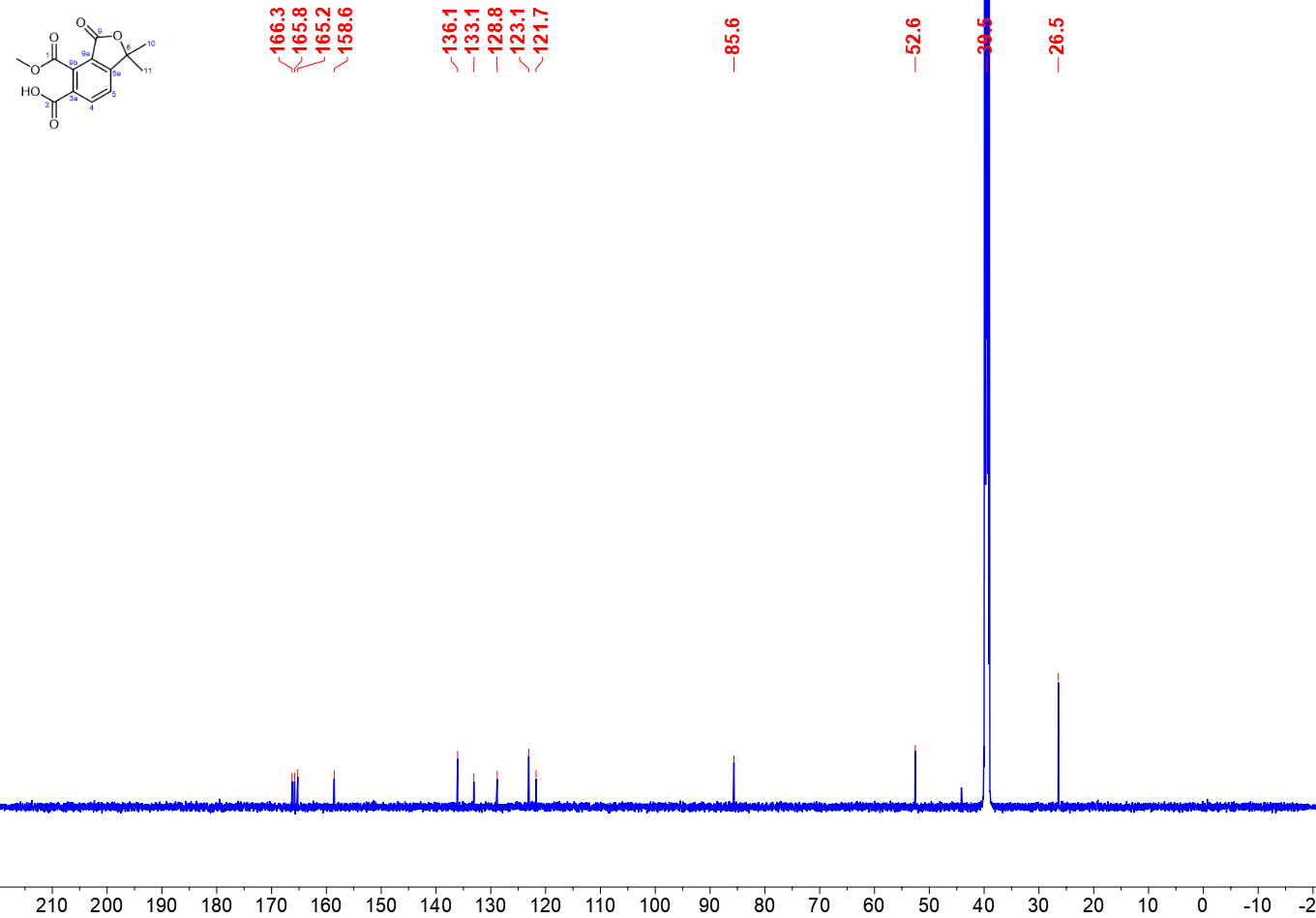
Figure S71. ^13^C NMR spectrum of **9.**


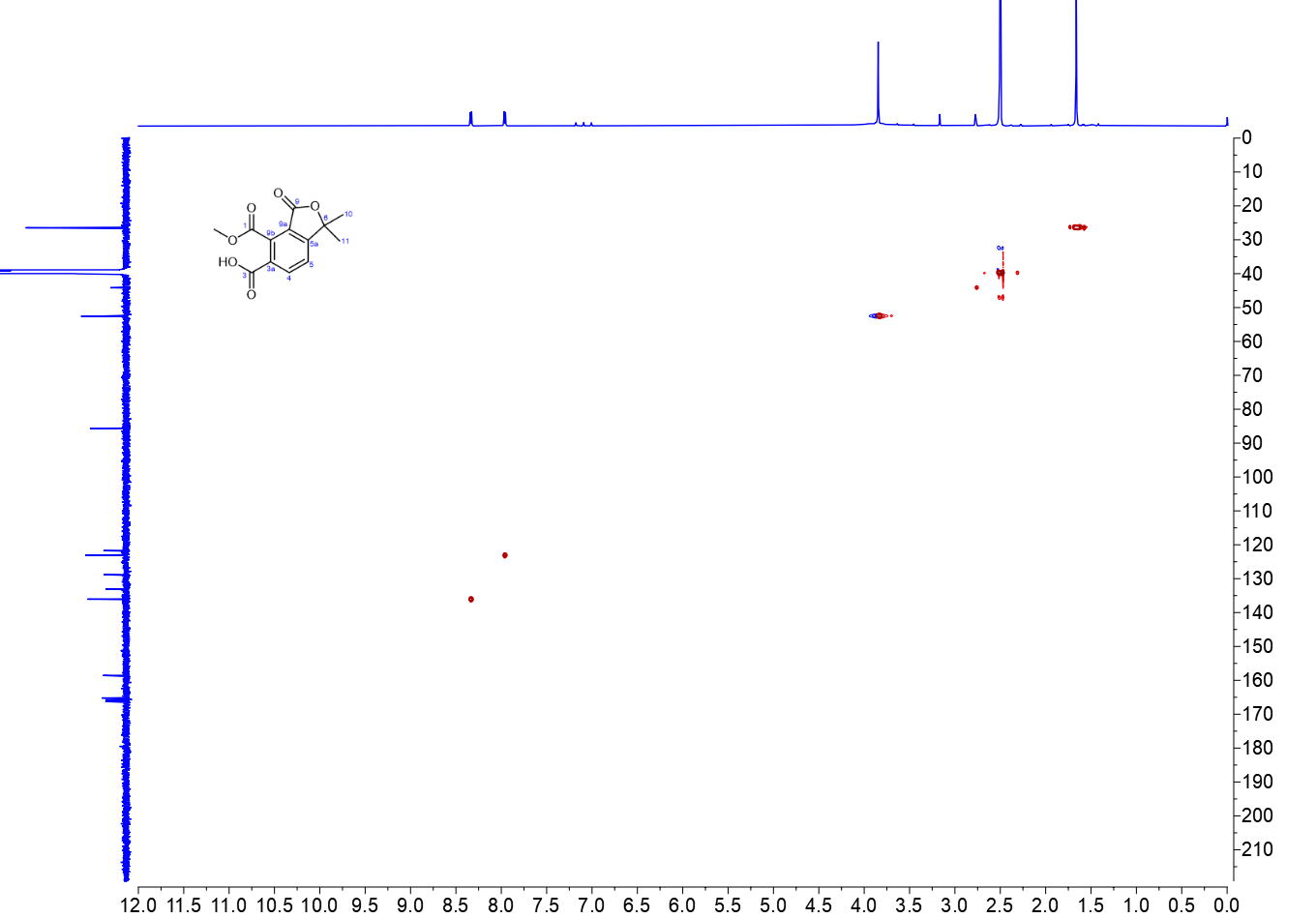
Figure S72. HSQC spectrum of **9.**


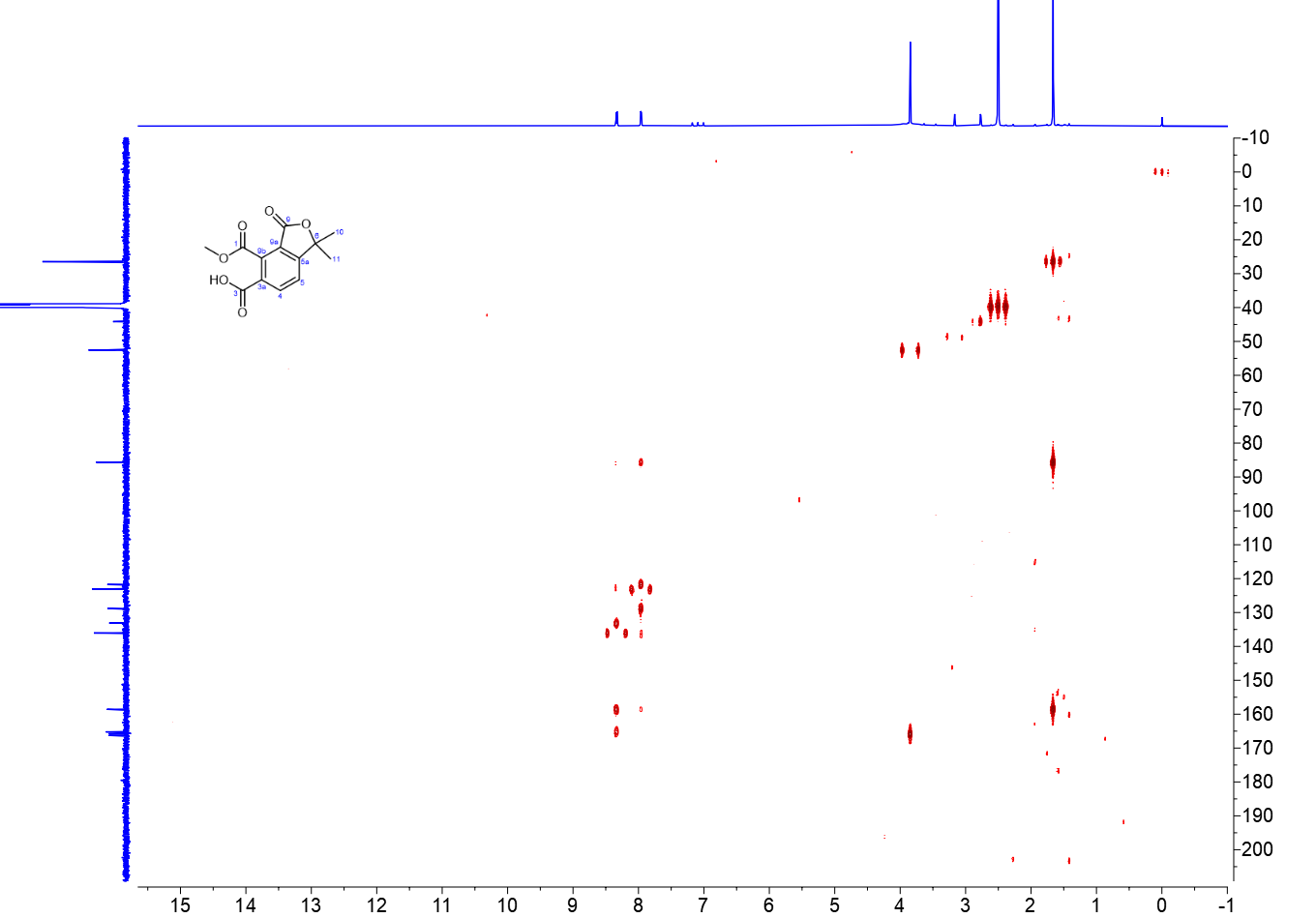
Figure S73. HMBC spectrum of **9.**


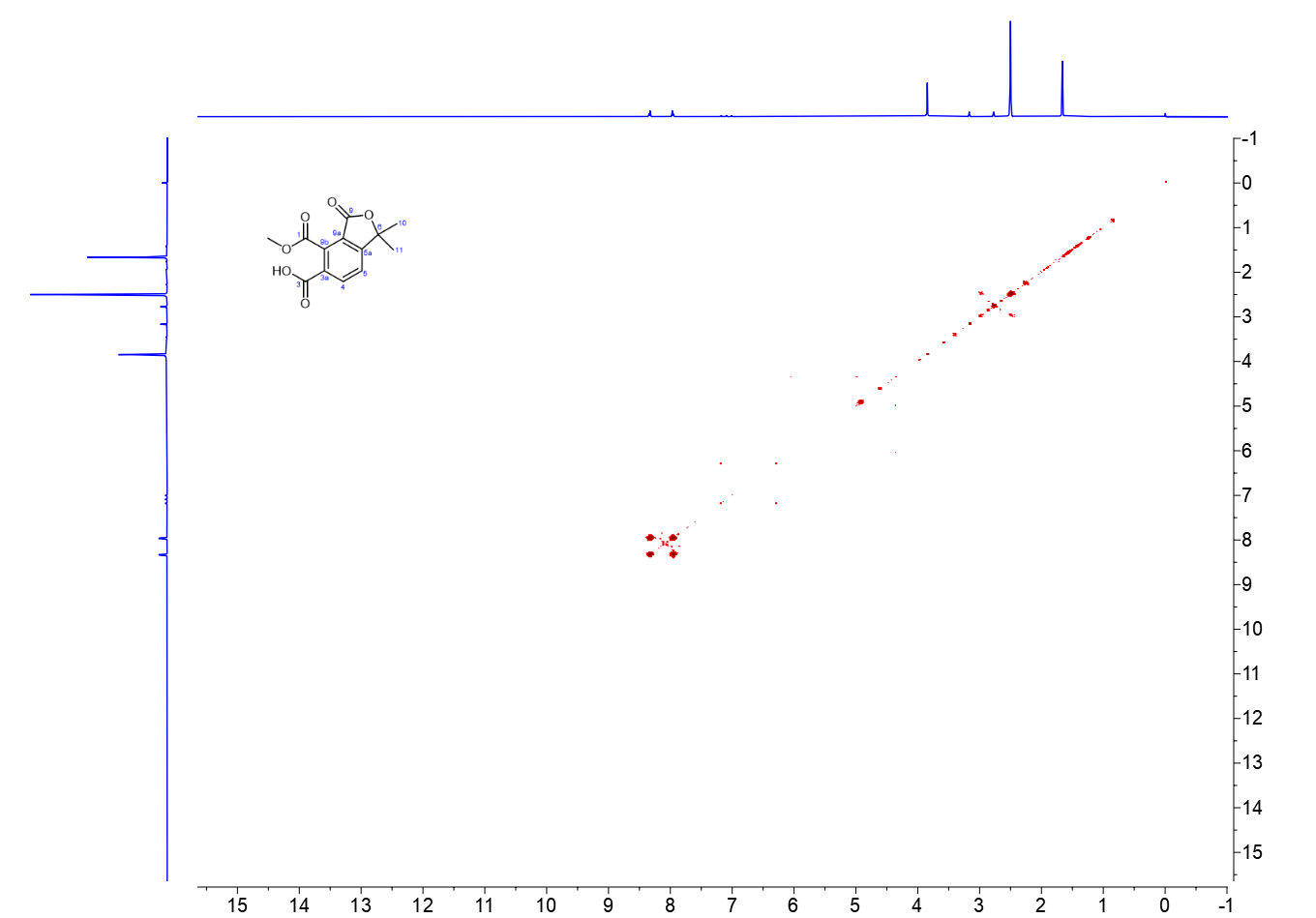
Figure S74. ^1^H-^1^H COSY spectrum of **9.**


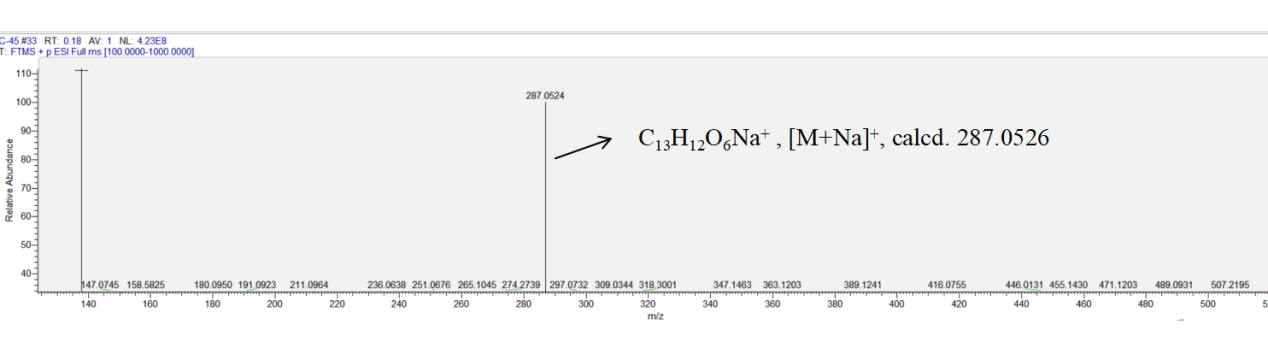
Figure S75. HRESIMS spectrum of **9.**





Figure S76. The UV spectrum of compound **9.**


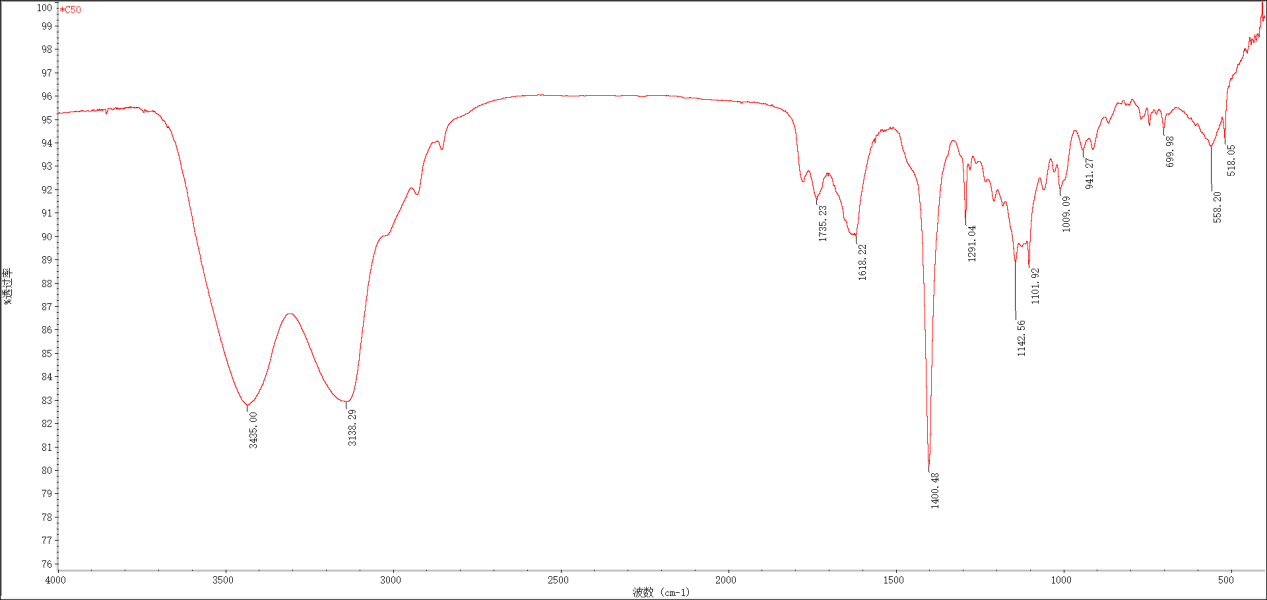
Figure S77. The IR spectrum of compound **9.**


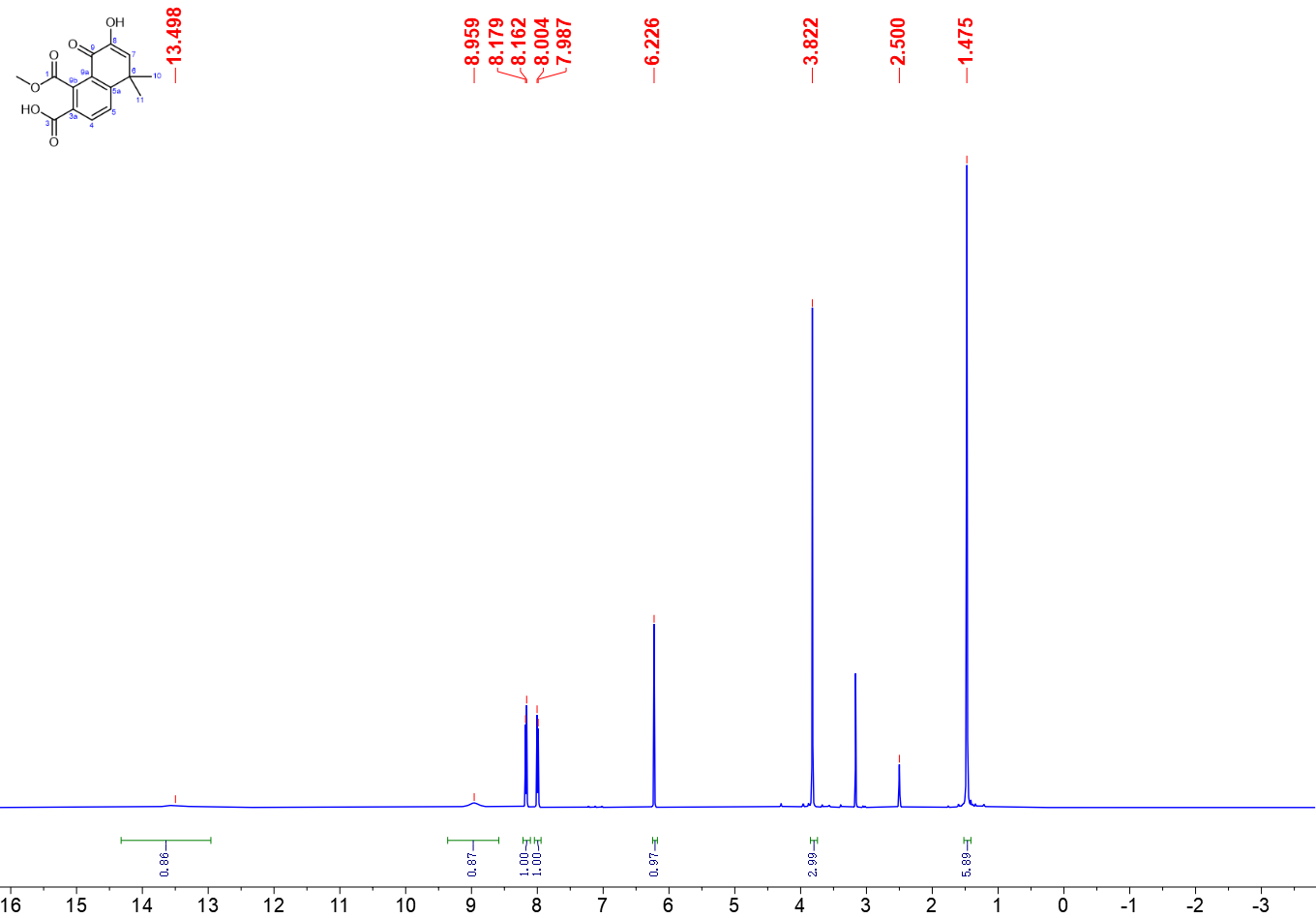
Figure S78. ^1^H NMR spectrum of **10.**


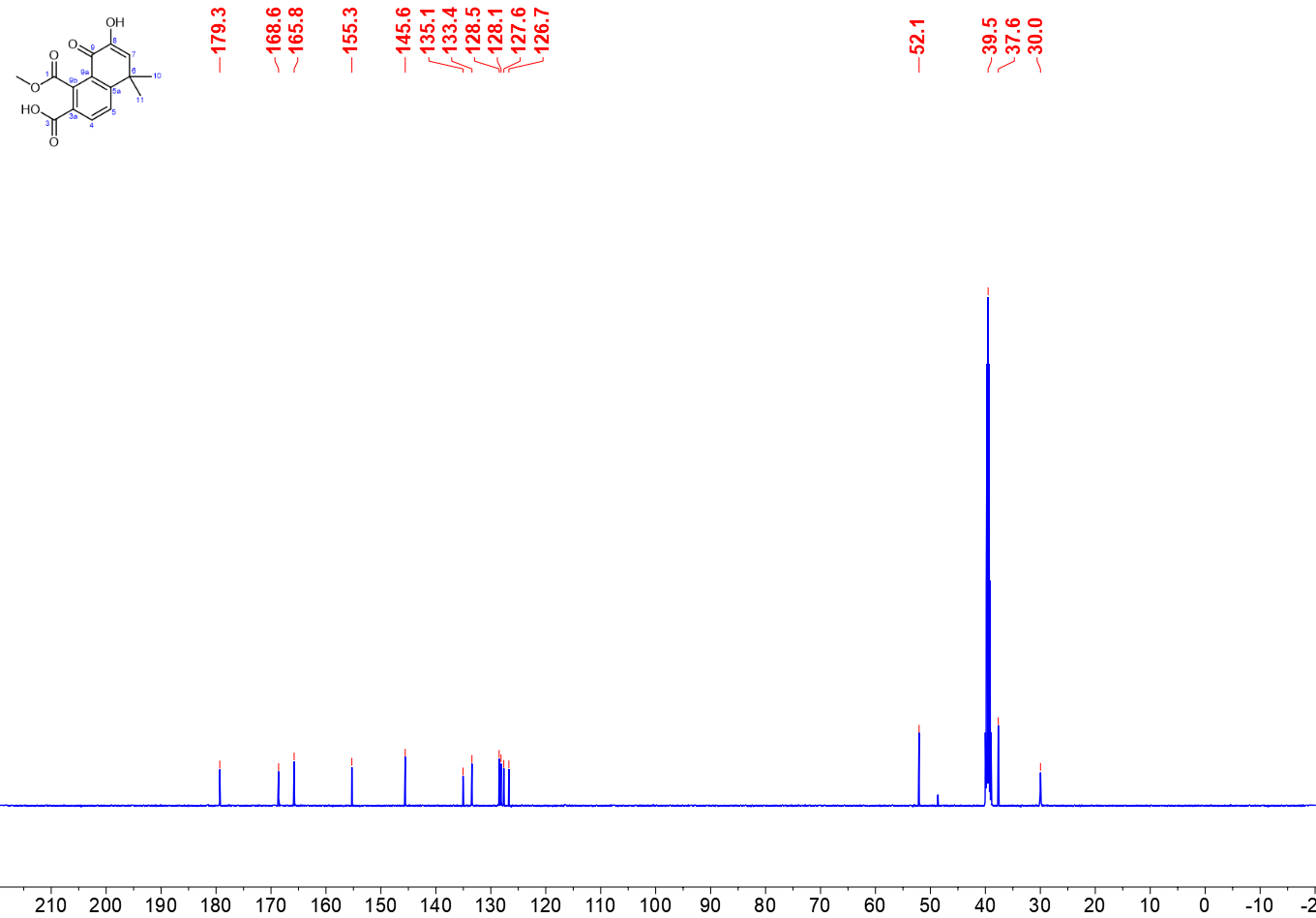
Figure S79. ^13^C NMR spectrum of **10.**


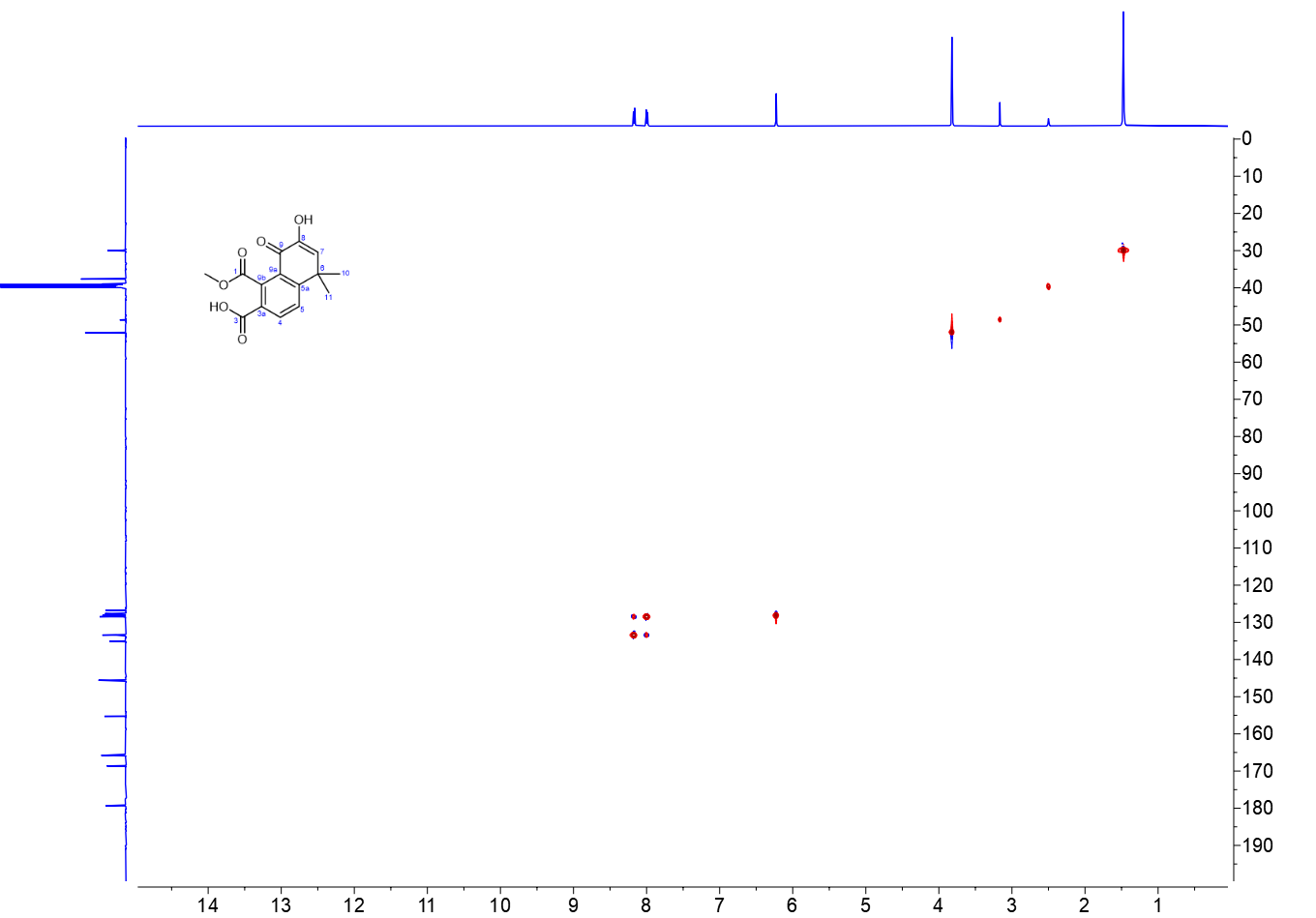
Figure S80. HSQC spectrum of **10.**


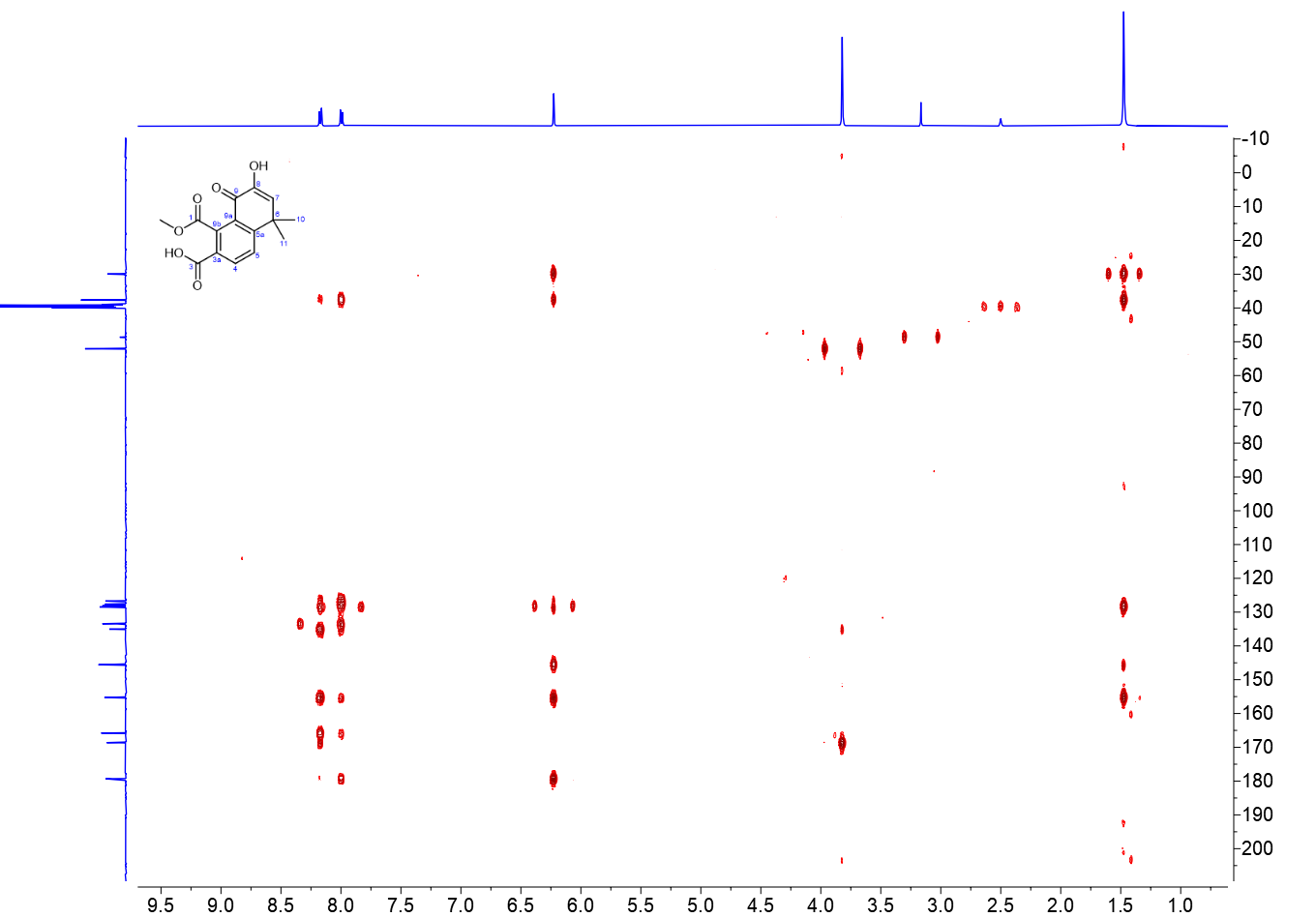
Figure S81. HMBC spectrum of **10.**


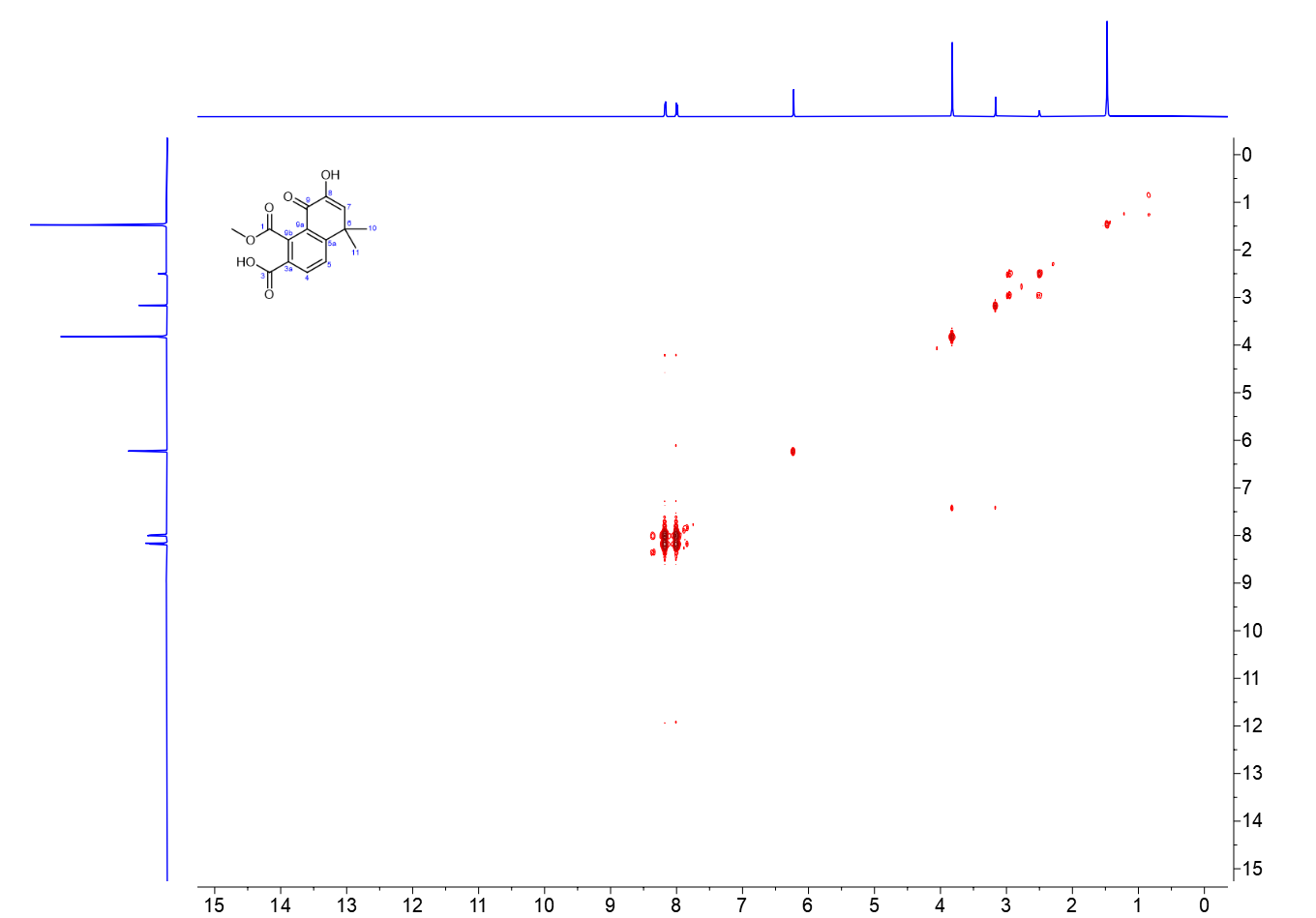
Figure S82. ^1^H-^1^H COSY spectrum of **10.**


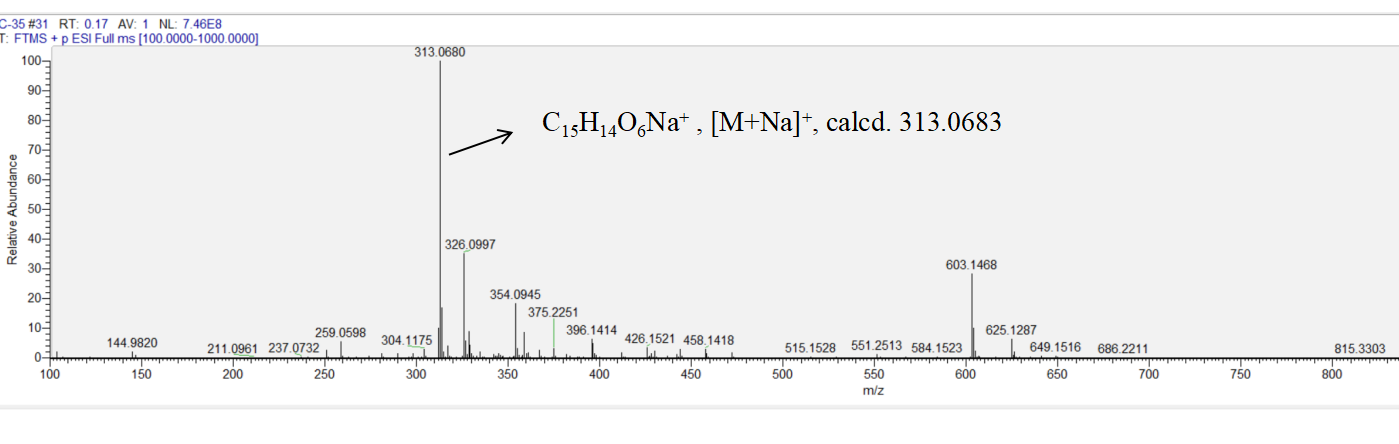
Figure S83. HRESIMS spectrum of **10.**





Figure S84. The UV spectrum of compound **10.**


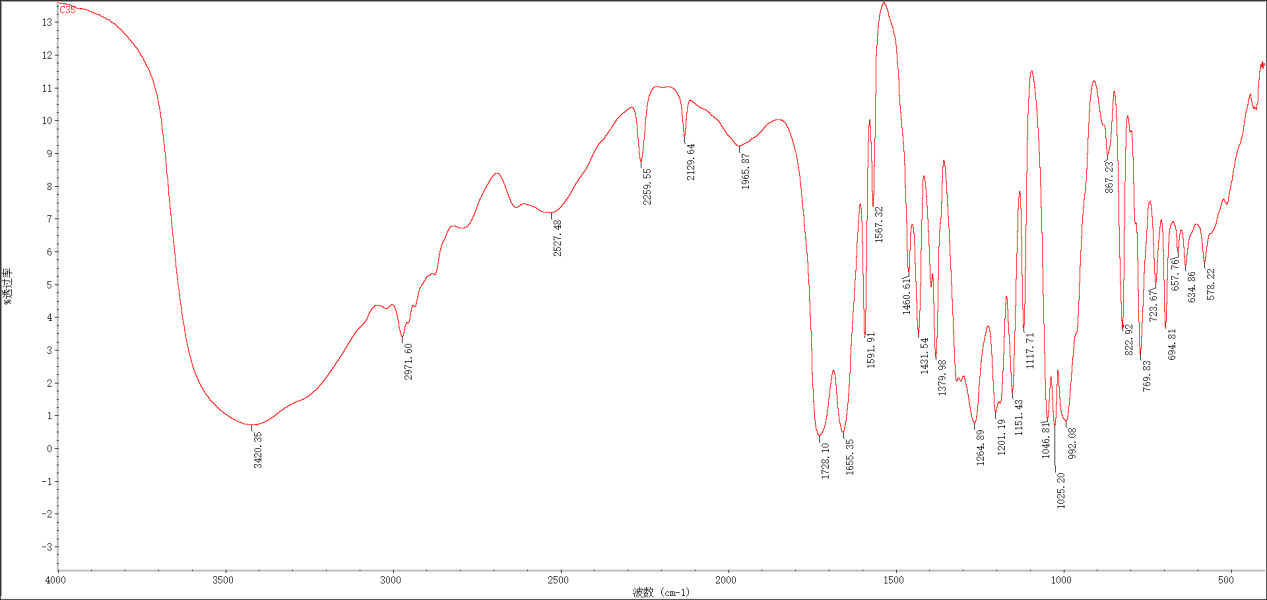
Figure S85. The IR spectrum of compound **10.**
